# Transcriptomic analysis for the retested positive COVID‐19 patients with long‐term persistent SARS‐CoV‐2 but without symptoms in Wuhan

**DOI:** 10.1002/ctm2.1172

**Published:** 2023-01-07

**Authors:** Cuidan Li, Liya Yue, Yingjiao Ju, Jie Wang, Hao Lu, Lin Li, Mengfan Chen, Chenyang Wang, Shuangshuang Li, Tao Liu, Sitong Liu, Tianyi Lu, Jing Wang, Xin Hu, Chunlai Jiang, Dongsheng Zhou, Fei Chen

**Affiliations:** ^1^ CAS Key Laboratory of Genome Sciences & Information, Beijing Institute of Genomics Chinese Academy of Sciences and China National Center for Bioinformation Beijing China; ^2^ University of Chinese Academy of Sciences Beijing China; ^3^ State Key Laboratory of Pathogen and Biosecurity Beijing Institute of Microbiology and Epidemiology Beijing China; ^4^ State Key Laboratory of Pathogenesis, Prevention Treatment of High Incidence Diseases in Central Asia Xinjiang China; ^5^ National Engineering Laboratory for AIDS Vaccine, School of Life Science Jilin University Changchun China; ^6^ Beijing Key Laboratory of Genome and Precision Medicine Technologies Chinese Academy of Sciences and China National Center for Bioinformation Beijing China

Dear Editor

A new type of COVID‐19 patients has been reported (referred as LTPPs) recently, who were retested with positive results after hospital discharge, with long‐term persistent SARS‐CoV‐2 in their bodies, although they were recovered from acute infection with no clinical symptom of COVID‐19.^1^, ^2^ They pose new challenges to the prevention and treatment of COVID‐19, but the underlying mechanism for such a contradictory phenomenon remains uncovered.

To address this issue, we performed transcriptomic analyses on the peripheral blood mononuclear cells (PBMCs) from 12 LTPPs at long‐term positive and second recovery stages, with the longest positive time of 132 days (Table S1). They were all diagnosed as moderate patients during the acute infection period (first admission time: January 14, 2020 to March 28, 2020), and then discharged due to two consecutive negative reverse transcription polymerase chain reaction (RT‐PCR) results in other hospitals, followed by a 14‐day‐quarantine‐period. However, in the 14‐day‐quarantine‐period, they were recharged to Wuhan Pulmonary Hospital owing to the recurrent positive RT‐PCR results, although they showed no clinical symptoms of COVID‐19 (Table S2). They were final discharged (May 4, 2020 to June 10, 2020) as recovery patients (RPs) according to Chinese discharge standards. Fifteen PBMC samples from healthy‐individuals were also included as controls (HCs).

Principal component analysis (PCA) showed differential expression of mRNAs, lncRNAs and miRNAs (adjusted *P*‐value < .05) among LTPPs, RPs, and HCs (Figure 1A, S1−S2, Tables S3−S5). Further analysis identified 1708, 962 and 1280 differentially expressed genes (DEGs), 1503, 623, and 1218 DElncRNAs, and 437, 341, and 312 DEmiRNAs in the LTPP/HC, RP/HC and RP/LTPP groups, respectively. Here, the majority of DEGs (68.44%) were significantly up‐regulated, while the majority of DElncRNAs (71.99%) were significantly down‐regulated in LTPPs (Figure 1B).

**FIGURE 1:**
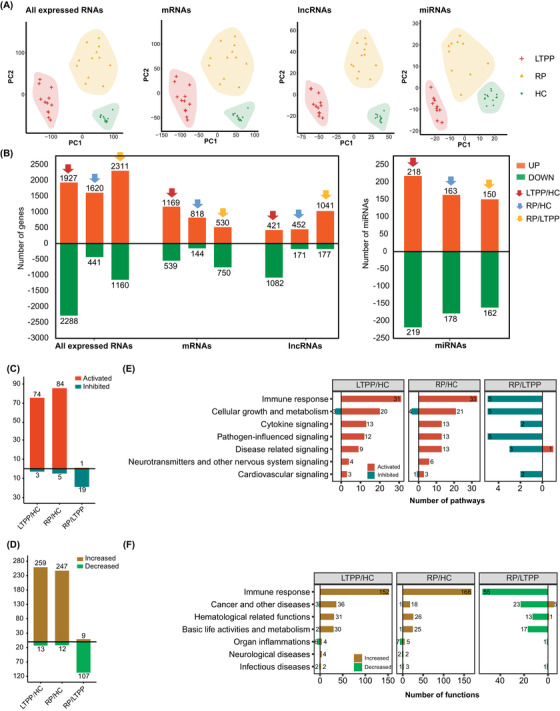
Significantly differentially expressed RNAs, significantly enriched pathways and biofunctions among the LTPP/HC, RP/HC and RP/LTPP groups. (A) PCA showing differential expressed profiles for all expressed RNAs, mRNAs, lncRNAs and miRNAs among the three groups. (B) Bar plots representing the number of significantly differentially expressed RNAs, mRNAs, lncRNAs and miRNAs among the three groups. Orange and green bars indicate significantly up‐regulated and down‐regulated RNAs, respectively. Bar plots showing the significantly enriched pathways (C) and biofunctions (D) among the LTPP/HC, RP/HC and RP/LTPP group; bar plots showing the significantly enriched pathways (E) and biofunctions (F) in seven aspects among the LTPP/HC, RP/HC and RP/LTPP groups. Red and yellow bars indicate the significantly up‐regulated/activated pathways/biofunctions (*Z*‐score ≥ 2, *P*‐value < .05), respectively; blue and green bars represent the significantly inhibited/down‐regulated pathways/biofunctions (*Z*‐score ≤ −2, *P*‐value < .05), respectively.

We then performed Ingenuity Pathway Analysis (IPA) to explore the enriched pathways/biofunctions responsible for LTPPs. The results revealed 77 enriched pathways and 272 enriched biofunctions in LTPPs, most of which (96.10%, 95.22%) were significantly up‐regulated (Figure 1C−F). Among them, the immune‐response related enriched pathways (31) and functions (152) ranked first in the LTPP/HC group (Figure 1E, F), all of which were significantly up‐regulated, including many innate‐immune related pathways (23) and biofunctions (35), and acquired immune related pathways (14) and biofunctions (11) (Figure 2A,B). This might explain the suppression of the emergence of clinical symptoms of COVID‐19 through fighting against the long‐term persistent SARS‐CoV‐2. Significant activation of acquired immune‐responses were also indicated by higher proportion of T lymphocyte, normal range of lymphocyte counts, and high neutralization abilities against SARS‐CoV‐2 (Figure 2C,D, Figure S3, Table S2).

**FIGURE 2:**
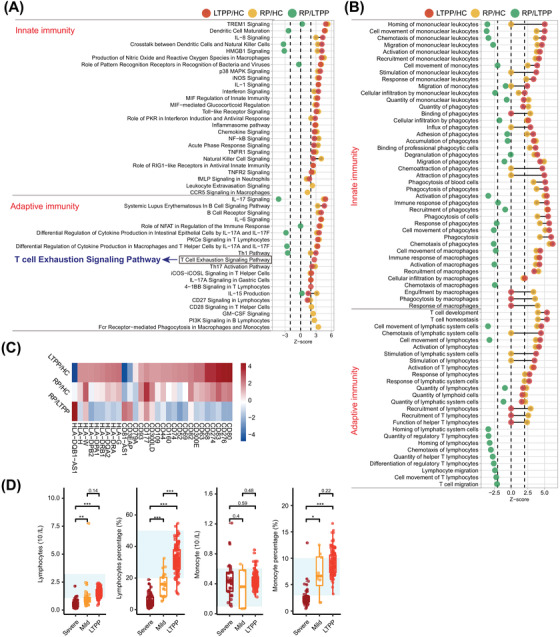
Significantly enriched pathways and functions associated with immune responses among the three groups. Dumbbell charts show the innate and adaptive immunity pathways (A) and functions (B) among the three groups. Pathway prediction is determined by *Z*‐score: positive values indicate up‐regulation; negative values indicate down‐regulation; |*Z*‐score| ≥ 2 represents the significant enriched pathways/functions. (C) Heatmap showing the significantly differentially expressed CD and HLA genes among the three groups. (D) Comparison of the lymphocytes and monocytes among the severe, mild and long‐term positive patients with COVID‐19. The normal ranges of lymphocytes and monocytes are shown as cyan box. Differences between groups are estimated using *t*‐test.

Why was not SARS‐CoV‐2 completely eliminated in LTPPs by highly activated immune‐responses? We found that all activated immune related pathways/functions in our study have been reported to facilitate the resistance/elimination of foreign pathogens, except “T‐Cell Exhaustion Signaling Pathway” (Figure 2A,B).^3^ Since T‐cell exhaustion can decrease the effector function and proliferative capacity of T‐cell,^3^, ^4^ it may be a key reason for the long‐term persistence of SARS‐CoV‐2 in LTPPs. Previous studies have also demonstrated that the persistent antigen stimulation from pathogens can result in T‐cell exhaustion.^3^ This was further supported by the fact that the pathway was recovered to normal in RPs (Figure 2A). Here, all 22 enriched genes were significantly up‐regulated in LTPPs, therefore, the inhibitors for these “T‐cell exhaustion” related genes (IL6, HLA‐G, etc.) provided some promising therapeutic drugs to treat long‐term infection of SARS‐CoV‐2 by reversing the progression of T cell exhaustion, while further studies are warranted.

We further determined key hub genes (hereinafter, KHGs) in the transition from LTPP to RP steady‐states based on the number of pathways/biofunctions they participated in (Figure 3A−D), since genes are more important in the contributions for clinical symptoms if they participate in more pathways/functions.^5^ We first listed the top‐10 KHGs for enriched pathways and biofunctions in the LTPP/RP group, and 21 KHGs were obtained, all of which showed significant upregulation (Figure 3A−D).

**FIGURE 3:**
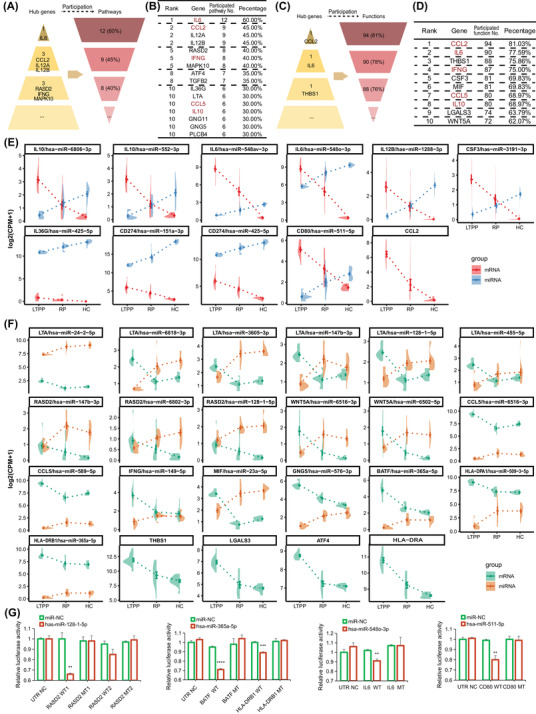
The 22 differentially expressed genes (DEGs) and 25 corresponding miRNAs played roles in the transition from LTPPs to RPs as potential therapeutic targets. (A) Schematic diagram showing the number and percentage of the pathways participated by each DEGs. (B) The top‐10 DEGs with more participated pathways. (C) Schematic diagram showing the number and percentage of the functions participated by each DEGs. (D) The top‐10 DEGs with more participated functions. (E) The eight KHGs with continuously down‐regulated expression trends and corresponding 10 miRNAs with continuously up‐regulated expression trends. (F) The 14 restored KHGs in the RPs and corresponding 15 recovered miRNAs in the RPs. (G) The dual‐luciferase reporter assay showing the validated five miRNA–mRNA pairs. HEK293T cells were co‐transfected with miRNA mimics/miR‐NC (negative control mimic) and 3′UTR‐WT (wild type)/MT (mutant) constructs. Empty vector was transfected as a negative control. Firefly luciferase activity was first normalised against Renilla luciferase activity, and relative luciferase activity of miRNA mimic transfected cells was then compared to those of miR‐NC transfected cells. The data were presented as the means ± SD (*n* = 3). Unpaired two‐tailed *t*‐test was used. **P < .01, ***P < .001, ****P < .0001.

Collectively, through exploring critical pathways and key hub genes in the transition from LTPP to RP steady‐states, we screened out 38 potential therapeutic targets (12 cytokines, 6 transcription regulator, 6 transmembrane receptors, etc.) for LTPPs (with shared five genes) in our study, including 22 enriched genes in the “T Cell Exhaustion Signaling Pathway” and 21 KHGs, all of which were significantly activated/up‐regulated in LTPPs. Importantly, 26 of 38 genes have been reported to be associated with COVID‐19, and six ones (IL6, CSF3, MIF, ATF4, HLA‐G and LGALS3) have been documented to be potential therapeutic targets for the treatment of COVID‐19 (Table 1), indicating the credibility of our screening strategy. More importantly, 8 of 38 genes showed a continuous trend of down‐regulated expression from LTPPs to RPs and then to HCs (IL6, IL10, IL12B, CSF3, IL36G, CCL2, CD274 and CD80), and 14 genes were restored to normal in the RPs (CCL5, LTA, RASD2, WNT5A, IFNG, MIF, ATF4, GNG5, THBS1, LGALS3, BATF, HLA‐DPA1, HLA‐DRA and HLA‐DRB1), which displayed consistent expression tendency with disease recovery, indicating their potential roles during the transitional process from LTPPs to RPs.

**TABLE 1:** The 38 potential therapeutic targets for the treatment of LTPPs

Gene name	Molecular types	KHG	T cell exhaustion	Reported COVID‐19 related genes	Reported potential COVID‐19 therapeutic target	Pattern*	LTPP/HC (Log2FC)	LTPP/RP	PMID
IL6	cytokine	√	√	√	√	HML	10.36919	4.055549	33850327
CCL2	cytokine	√		√		HML	9.940254	5.526238	32446778
CCL5	cytokine	√		√		HLL	1.984041	2.833436	32446778
CSF3	cytokine	√		√	√	HML	7.082919	2.537436	33551833
MIF	Cytokine	√		√	√	HLL	2.791618	3.811961	33671433
LTA	Cytokine	√		√		HLL	1.503052	1.987398	33939073
WNT5A	Cytokine	√		√		HLL	4.664088	3.277689	32859680
IL36G	Cytokine	√		√		HML	4.291546	2.072732	32228226
IL12B	Cytokine	√	√	√		HML	7.396001	3.120852	32446778
IFNG	Cytokine	√	√	√		HLL	2.415187	2.165656	33362782
IL10	Cytokine	√	√	√		HML	4.960022	3.029283	32446778
IL12A	Cytokine	√		√			–	1.726286	33780352
PLCB4	Enzyme	√		√			–	2.146549	33839760
RASD2	Enzyme	√	√	√		HLL	3.128655	2.062861	33847347
MRAS	Enzyme		√				2.418406	1.106525	
CD274	Enzyme		√	√		HML	3.438564	1.613298	34330510
PDCD1LG2	Enzyme		√				1.94056	0.518319	
TGFB2	Growth factor	√		√			–	1.599246	33174447
MAPK10	Kinase	√		√			–	1.698625	34360824
ACVR2A	Kinase		√				2.551106	1.243274	
LGALS3	Other	√		√	√	HLL	2.341823	2.051543	32496587, 34109210
GNG11	Other	√		√			1.874657	3.847569	32934806
THBS1	Other	√		√		HLL	3.617571	2.609973	34027422
GNG5	Other	√		√		HLL	2.32511	1.530594	32959892
HLA‐G	Other		√	√	√		2.264023	1.286491	33046268
HAVCR2	Other		√				1.509534	0.853113	
ATF4	transcription regulator	√		√	√	HLL	1.660442	1.524425	34360824, 35028850
FOS	Transcription regulator		√	√			1.596024	‐0.67849	33241658
BCL6	Transcription regulator		√	√			2.302922	‐0.45369	32877699
BATF	Transcription regulator		√			HLL	3.103866	2.408887	
PRDM1	Transcription regulator		√				1.541112	0.99707	
STAT1	Transcription regulator		√	√			1.666579	0.237303	34676541
HLA‐DPA1	Transmembrane receptor		√			HLL	1.847346	1.716263	
HLA‐DQA2	Transmembrane receptor		√				2.101604	1.162313	
HLA‐DRA	Transmembrane receptor		√			HLL	2.198501	1.554854	
HLA‐DRB1	Transmembrane receptor		√			HLL	1.897915	1.692554	
CD80	Transmembrane receptor		√			HML	4.225086	2.159492	
FCER1G	Transmembrane receptor		√				2.616964	1.086032	

^*^HML: the expressions in LTPPs, RPs and HCs show continuously downregulation from high to middle then to low; HLL: LTPPs, RPs and HCs show high, low and low expression levels, respectively.

We then identified 65 down‐regulated DEmiRNAs targeting the 38 up‐regulated targeted genes (Figure S4, Table S6), since previous studies indicated that human body could activate/up‐regulate some genes to fight against diseases through epigenetic downregulating corresponding miRNAs.^6^, ^7^ Importantly, 25 of 65 miRNAs showed reversed expressed trend with corresponding 17 targeted genes (Figure 3E,F), displaying consistent tendency with disease recovery duration, suggesting their important roles in the recovery of LTPPs. We further focused on seven miRNA–mRNAs pairs with high confidence prediction of targeting relationships, among which four miRNAs (hsa‐miR‐511‐5p, hsa‐miR‐128‐1‐5p, hsa‐miR‐365a‐5p and hsa‐miR‐548o‐3p) targeting five mRNAs (RASD2, IL6, BATF, HLA‐DRB1 and CD80) were validated using 3′UTR‐luciferase assay (Figure 3G, Figure S5). Further functional validations for these genes and miRNAs (especially for unreported ones) and their potential therapeutic values are worthy of further investigation.

Some limitations do exist in our study. Validation experiments cannot be performed since we do not have the required qualifications and license for handling SARS‐CoV‐2. Additionally, due to the serious conditions of COVID‐19 pandemic and the huge treatment burden of clinicians in Wuhan during Marchto June 2020, it was very difficult for us to pursue more analyses at that time (e.g., RT‐PCR, western‐blot, proteomic), which definitely will be helpful in this study. Additionally, the profiles of the COVID‐19 patients after vaccination are worthy of further investigation for comprehensively featuring COVID‐19.

In summary, our results uncovered the underlying immune mechanism for the contradictory phenomenon of LTPPs: while the significantly activated innate and acquired immune responses in the LTPPs could potentiate the body against long‐term persistent SARS‐CoV‐2 resulting in uncharacterized clinical COVID‐19 manifestations, the significantly activated T cell exhaustion pathways might prevent SARS‐CoV‐2 from complete elimination, leading to their long‐term survival in the LTPPs. Our findings also provide the important references and improved understanding of COVID‐19.

## CONFLICT OF INTEREST

The authors declare that there is no conflict of interest that could be perceived as prejudicing the impartiality of the research reported.

## Supporting information

Figure S1

Figure S2

Figure S3

Figure S4

Figure S5

Supporting Information
