# Supplementary material for: Transcriptomic analysis for the retested positive COVID‐19 patients with long‐term persistent SARS‐CoV‐2 but without symptoms in Wuhan
Source: Clin Transl Med. 2023 Jan 7;13(1):e1172. doi: 10.1002/ctm2.1172 (PMC9825106; doi:10.1002/ctm2.1172)
Supplement: Supplementary file 6 — Supporting Information [file CTM2-13-e1172-s003.docx]

**SUPPLEMENTARY DATA**

**Transcriptomic analyses for the retested positive COVID-19 patients with long-term persistent SARS-CoV-2 but without symptoms in Wuhan**

Cuidan Li^1#^, Liya Yue^1#^, Yingjiao Ju^1,2#^, Jie Wang^1,2#^, Hao Lu^1,2#^, Lin Li^3#^, Mengfan Chen^1,2^, Chenyang Wang^1,2^, Shuangshuang Li^1,2^, Tao Liu^1,2^, Sitong Liu^1,2^, Tianyi Lu^1,2^, Jing Wang^4^, Xin Hu^4^, Chunlai Jiang^5^, Dongsheng Zhou^3^*, Fei Chen^1,2,4,7^*

**This file includes:**

[Table S1](#_Table_S1._Clinical) Clinical characteristics for the 12 long-term positive patients with COVID-19

[Table S2](#_Table_S2._Clinical) Clinical laboratory findings for the 12 LTPPs

[Table S3](#_Table_S3._The_1) The lncRNA targets in the LTPP/HC group

[Table S4](#_Table_S4._The_1) The lncRNA targets in the RP/HC group

[Table S5](#_Table_S5._The_1) The lncRNA targets in the LTPP/RP group

[Table S6](#_Table_S6._The) The 65 miRNAs targeting to 38 DEGs

Supplementary Figure Legends

## Table S1. Clinical characteristics for the 12 long-term positive patients with COVID-19

| **Patient ID** | **LTPP ID** | **RP**  **ID** | **Age** | **Gender** | **Comorbidities** | **Positive time** | **Plasma treatment** | **MN100* in LTPP (Days)** | **MN100* in RP (Days)** |  |
| --- | --- | --- | --- | --- | --- | --- | --- | --- | --- | --- |
| Patient1 | LTPP01 | RP01 | 78 | F | hypertension, diabetes | 132 | **Yes** | 640 (86) | 640 (107) |  |
| Patient2 | LTPP02 | RP02 | 44 | M | hypertension | 127 | No | 80 (35) | 40 (57) |  |
| Patient3 | LTPP03 | RP03 | 68 | F | hypertension, diabetes, coronary | 123 | No | 80 (54) | 40 (116) |  |
| Patient4 | LTPP04 | RP04 | 46 | M | No | 109 | **Yes** | 80 (83) | 40 (111) |  |
| Patient5 | LTPP05 | RP05 | 79 | F | hypertension, chronic bronchitis | 109 | **Yes** | 160 (103) | 80 (168) |  |
| Patient6 | LTPP06 | RP06 | 63 | M | renal calculi | 107 | No | 80 (70) | 40 (109) |  |
| Patient7 | LTPP07 | RP07 | 65 | M | No | 94 | No | 640 (90) | - |  |
| Patient8 | LTPP08 | RP08 | 37 | M | No | 94 | No | 40 (94) | 80 (164) |  |
| Patient9 | LTPP09 | RP09 | 47 | M | hypertension | 88 | **Yes** | 160 (72) | 80 (90) |  |
| Patient10 | LTPP10 | RP10 | 69 | M | tuberculosis | 84 | No | 160 (98) | 80 (132) |  |
| Patient11 | LTPP11 | RP11 | 62 | F | No | 55 | No | 10 (73) | 10 (93) |  |
| Patient12 | LTPP12 | RP12 | 16 | M | No | 28 | No | 40 (109) | 80 (122) |  |
| **Summary** |  |  | 63  (46 – 68) | 8 males  (66.67%)  4 female  (33.33%) | 7 comorbidities (58.33%) | 101  (87 – 113) | 4 plasma treatment (33.33%) | 7 (58.33%)  2 (16.67%)  2 (16.67%) | |  |

***MN100: the neutralizing titre of SARS-CoV-2 measured using serum from LTPPs and RPs. Neutralizing titer was represented with the reciprocal of the highest serum dilution to achieve the 100% protection from virus infection.**

## Table S2. Clinical laboratory findings for the 12 LTPPs

| **Laboratory Findings** | **Normal Range** | **All patients (n = 12)** |
| --- | --- | --- |
| **Blood routine** |  |  |
| Leucocytes count, × 10^9^/L | 3.50 - 9.50 | 5.40 (4.19 - 6.04) |
| Erythrocyte count, × 10 ^12^/L | 3.80 - 5.10 | 4.24 (3.79 - 4.86) |
| Hemoglobin, g/L | 115.00 - 150.00 | 133.00 (117.75 - 148.00) |
| Hematocrit, % | 35.00 - 45.00 | 41.10 (35.55 - 43.73) |
| Platelets count, × 10 ^9^/L | 125.00 - 350.00 | 183.50 (162.75 - 217.75) |
| Platelet hematocrit, % | 0.10 - 0.50 | 0.17 (0.15 - 0.20) |
| MCV, fL | 82.00 - 100.00 | 93.45 (90.47 - 98.18) |
| MCH, pg | 27.00 - 34.00 | 30.80 (30.08 - 32.38) |
| MCHC, g/L | 316.00 - 354.00 | 331.76 (8.07) |
| MPV, fL | 9.00 - 13.00 | 9.40 (8.80 - 9.80) |
| PDW, fL | 9.00 - 17.00 | 16.09 (0.39) |
| P-LCR, % | 13.00 - 43.00 | 22.80 (18.50 - 25.30) |
| Lymphocytes count, × 10 ^9^/L | 1.10 - 3.20 | 1.55 (0.40) |
| Lymphocytes percentage, % | 20.00 - 50.00 | 31.15 (9.02) |
| Neutrophils count, × 10 ^9^/L | 1.80 - 6.30 | 3.14 (2.14 - 3.75) |
| Neutrophils percentage, % | 40.00 - 75.00 | 56.71 (10.04) |
| Monocyte, × 10 ^9^/ L | 0.10 - 0.60 | 0.42 (0.37 - 0.49) |
| Monocyte percentage, % | 3.00 - 10.00 | 8.30 (7.27 - 10.60) |
| Eosinophils, × 10 ^9^/L | 0.02 - 0.52 | 0.16 (0.08 - 0.21) |
| Eosinophil percentage, % | 0.40 - 8.00 | 2.75 (1.80 - 3.80) |
| Basophils, × 10 ^9^/ L | 0.00 - 0.06 | 0.02 (0.01 - 0.02) |
| Basophil percentage, % | 0.00 - 1.00 | 0.40 (0.30 - 0.50) |
| RDW-SD, fl | 37.00 - 50.00 | 43.05 (41.68 - 45.90) |
| RDW-CV, % | 11.00 - 15.00 | 13.20 (12.80 - 13.72) |
|  |  |  |
| **Blood biochemistry** |  |  |
| Alanine aminotransferase, U/L | 7.00 - 40.00 | 17.00 (12.00 - 31.00) |
| Aspartate aminotransferase, U/L | 13.00 - 35.00 | 20.00 (17.00 - 31.00) |
| Alkaline phosphatase, IU/L | 35.00 - 100.00 | 72.51 (18.76) |
| γ-glutamyl transpeptidase, IU/L | 7.00 - 45.00 | 24.00 (16.00 - 55.00) |
| Total protein, g/L | 60.00 - 83.00 | 65.80 (61.00 - 71.60) |
| Albumin, g/L | 35.00 - 55.00 | 42.00 (39.20 - 44.80) |
| Globulin, g/L | 20.00 - 46.00 | 23.92 (5.62) |
| A/G | 1.10 - 2.50 | 1.75 (1.47 - 2.18) |
| Total bilirubin, μmol/L | 0.00 - 21.00 | 8.58 (5.58 - 12.62) |
| Direct bilirubin, μmol/L | 0.00 - 6.80 | 2.03 (1.30 - 2.81) |
| Urea, mmol/L | 1.70 - 8.30 | 4.59 (4.02 - 5.57) |
| Serum creatinine, μmol/L | 44.00 - 115.00 | 60.00 (49.00 - 78.00) |
| Uric acid, μmol/L | 150.00 - 490.00 | 355.00 (246.00 - 466.00) |
| Glucose, mmol/L | 3.50 - 6.10 | 5.26 (4.80 - 6.15) |
| Total cholesterol, mmol/L | 2.30 - 5.20 | 4.13 (2.83 - 5.06) |
| Triglyceride, mmol/L | 0.50 - 1.90 | 1.72 (0.49) |
| Adenosine deaminase, U/L | 0.00 - 25.00 | 10.90 (9.30 - 13.40) |
| Lactate dehydrogenase, U/L | 106.00 - 245.00 | 170.00 (152.00 - 208.00) |
| K, mmol/L | 3.50 - 5.30 | 3.87 (0.33) |
| Na, mmol/L | 136.00 - 145.00 | 137.00 (136.00 - 138.70) |
| Cl, mmol/L | 96.00 - 108.00 | 103.50 (101.30 - 105.80) |
| Ga, mmol/L | 2.10 - 2.70 | 2.43 (2.37 - 2.50) |
|  |  |  |
| **Coagulation function** |  |  |
| D-dimer, μg/L | 0.00 - 0.55 | 0.22 (0.11 - 0.35) |
| Activated partial thromboplastin time, s | 27.00 - 45.00 | 36.00 (34.05 - 37.85) |
| Prothrombin time, s | 11.00 - 16.00 | 12.80 (12.50 - 13.22) |
| INR | 0.80 - 1.03 | 0.98 (0.95 - 1.02) |
| Fibrinogen, g/L | 2.00 - 4.00 | 3.07 (2.62 - 3.39) |
| Phrombin time, s | 12.00 - 20.00 | 15.70 (15.30 - 16.40) |
|  |  |  |
| **Inflammatory-related biomarkers** |  |  |
| C-reactive protein, mg/L | 0.00 -5.00 | 1.48 (0.70 - 3.81) |
| Interleukin-6, pg/mL | 0.00 - 7.00 | 1.50 (1.50 - 3.78) |
| Procalcitonin, ng/mL | 0.00 -0.046 | 0.03 (0.02 - 0.05) |
| Erythrocyte sedimentation rate, mm/h | 0.00 - 15.00 | 24.00 (10.00 - 50.00) |
|  |  |  |
| **T cells Subsets** |  |  |
| Th cells (CD3 ABS) /ul | 625 - 2460 | 992.66 (812.40 - 1245.11) |
| Th cells ( %ly） | 60 - 90 | 71.29 (9.02) |
| Th cells (CD3+CD4+) /ul | 550.00 - 1440.00 | 626.18 (207.94) |
| Th cells (CD3+CD4+) % | 27.00 - 51.00 | 43.35 (38.61 - 46.70) |
| Ts cells (CD3+CD8+) /ul | 320.00 -1250.00 | 348.93 (253.03 - 520.84) |
| Ts cells (CD3+CD8+) % | 15.00 - 44.00 | 24.43 (17.98 - 31.57) |
| Th/Ts | 0.71 - 2.78 | 1.69 (1.29 - 2.55) |
| Th cells (CD45 ABS) |  | 1436.63 (1177.54 - 1703.85) |
|  |  |  |
| **Immunoglobulins** |  |  |
| Immunoglobulin A | 0.82 - 4.53 | 2.66 (1.06) |
| Immunoglobulin G | 7.51 - 15.60 | 15.80 (4.03) |
| Immunoglobulin M | 0.46 - 3.04 | 0.86 (0.62 - 1.40) |
|  |  |  |
| **Complement proteins** |  |  |
| C3 | 0.65 - 1.39 | 1.11 (1.00 - 1.18) |
| C4 | 0.16 - 0.38 | 0.23 (0.18 - 0.27) |
|  |  |  |
| **Cardiac biomarkers** |  |  |
| Creatine kinase, U/L | 18.00 - 198.00 | 60.30 (37.95 - 109.55) |
| Creatine kinase isoenzymes, ng/ml | 0.00 - 5.86 | 1.00 (0.50 - 1.35) |
| Creatine kinase isoenzyme activity, U/L | 0.00 - 18.00 | 14.59 (3.42) |
| Cardiac troponin I | 0.00 - 0.10 | 0.01 (0.01 - 0.01) |
| NT-proBNP | 0.00 - 1800.00 | 27.14 (7.31 - 59.21) |
| Myoglobin, ng/ml | 0.00 - 100.02 | 17.79 (7.52) |

Note: Data are shown as mean (SD) or median (IQR). MCV: mean corpuscular volume; MCH: mean corpuscular hemoglobin; MCHC: mean corpuscular hemoglobin concentration; MPV: mean platelet volume; PDW: platelet distribution width; P-LCR: platelet large cell ratio; RDW-SD: red cell distribution width; RDW-CV: coefficient of variation of red cell distribution width; A/G: albumin/globulin; INR: international normalized ratio; Th cells: helper T cells; Ts cells: suppressor T cells.

## Table S3. The lncRNA targets in the LTPP/HC group

| **Lnc_Name** | **Lnc_log2FC(LTPP/HC)** | **Gene_Name** | **Gene_log2FC(LTPP/HC)** |
| --- | --- | --- | --- |
| RP5-1142A6.2 | -4.912363938 | PKD1 | -1.5128 |
| AC005329.7 | -3.477566523 | PKD1 | -1.5128 |
| RP11-849H4.4 | -5.856196443 | PKD1 | -1.5128 |
| LA16c-358B7.3 | -7.46151727 | PKD1 | -1.5128 |
| SPACA6 | 2.335156664 | SEMA3B | 1.675214 |
| STK24-AS1 | 1.778604657 | CD74 | 1.760435 |
| RP11-106M3.3 | -6.043017396 | FAM184B | -4.71238 |
| RP5-1142A6.2 | -4.912363938 | NRIP2 | -6.13631 |
| RP11-269F19.2 | -4.315098612 | NRIP2 | -6.13631 |
| KB-1208A12.3 | -8.38024941 | NRIP2 | -6.13631 |
| RP11-849H4.4 | -5.856196443 | NRIP2 | -6.13631 |
| CTB-31O20.4 | -7.245046692 | NRIP2 | -6.13631 |
| AC005944.2 | -9.087897143 | NRIP2 | -6.13631 |
| LLNLR-284B4.1 | -8.114263599 | NRIP2 | -6.13631 |
| CTBP1-AS | -7.633444677 | NRIP2 | -6.13631 |
| RP11-568N6.1 | 3.79276003 | TRAF3IP2 | 2.681703 |
| RP11-153M7.5 | 3.357032402 | MXD1 | 2.194075 |
| SPACA6 | 2.335156664 | DGAT2 | 2.641326 |
| CTD-2530H12.2 | 1.914535634 | DGAT2 | 2.641326 |
| CTD-2020K17.3 | -5.184076698 | GLTSCR1 | -2.33939 |
| RP11-849H4.4 | -5.856196443 | GLTSCR1 | -2.33939 |
| AP006621.8 | -7.320584436 | GLTSCR1 | -2.33939 |
| RP5-1142A6.5 | -5.73662872 | GLTSCR1 | -2.33939 |
| LA16c-358B7.3 | -7.46151727 | GLTSCR1 | -2.33939 |
| RP11-498C9.3 | -6.24175772 | GLTSCR1 | -2.33939 |
| CTD-2231E14.8 | -6.052319332 | GLTSCR1 | -2.33939 |
| SNHG6 | 2.264283447 | YBX1 | 2.083285 |
| CTA-29F11.1 | 4.39801455 | YBX1 | 2.083285 |
| SNHG15 | 1.921718711 | DNTTIP2 | 1.850712 |
| CTA-29F11.1 | 4.39801455 | DNTTIP2 | 1.850712 |
| ZEB2-AS1 | 2.447879212 | SRI | 1.504267 |
| LINC00936 | 4.057972374 | SRI | 1.504267 |
| RP11-342M3.5 | 2.07499391 | CD82 | 1.976601 |
| RP11-439L18.2 | 2.563149119 | DNAJA1 | 2.285795 |
| RP11-73E17.2 | 1.606414101 | PPP1R15A | 2.816271 |
| CTB-41I6.1 | 2.454428789 | PPP1R15A | 2.816271 |
| SNHG6 | 2.264283447 | RPL6 | 1.81698 |
| TRIM52-AS1 | 1.766004015 | RPL6 | 1.81698 |
| SNHG9 | 2.552371517 | RPL6 | 1.81698 |
| RP4-742C19.13 | -7.615473666 | ANGPT2 | -3.94141 |
| CTC-250I14.6 | -6.559146028 | HOOK2 | -2.9154 |
| RP11-568N6.1 | 3.79276003 | GADD45B | 3.461452 |
| AP000355.2 | 4.367811973 | UPB1 | 4.165011 |
| RP11-73E17.2 | 1.606414101 | NFKBIA | 3.312721 |
| CTB-41I6.1 | 2.454428789 | NFKBIA | 3.312721 |
| SNHG6 | 2.264283447 | PFDN4 | 1.534825 |
| AC093673.5 | 2.234154931 | SMS | 1.581957 |
| RP11-408H1.3 | 4.076872264 | IRG1 | 7.459408 |
| AF127936.5 | 1.968152446 | IRG1 | 7.459408 |
| SMG7-AS1 | 2.208511128 | IRG1 | 7.459408 |
| RP11-439A17.10 | 3.620850282 | IRG1 | 7.459408 |
| RP11-434D11.4 | 4.059155405 | IRG1 | 7.459408 |
| RP11-44K6.2 | 4.690661249 | IRG1 | 7.459408 |
| RP11-480C16.1 | 3.979218342 | IRG1 | 7.459408 |
| MIR222HG | 4.018234246 | EHD4 | 2.254281 |
| CTD-2382E5.6 | 2.222385218 | EHD4 | 2.254281 |
| RP5-827C21.4 | 2.037414441 | C19orf53 | 2.496654 |
| SNHG6 | 2.264283447 | C19orf53 | 2.496654 |
| RP11-598F7.3 | 3.20072637 | TNNT1 | 2.347389 |
| CTA-29F11.1 | 4.39801455 | CDC37 | 1.76717 |
| RP11-467L13.7 | 3.761557831 | SIGLEC5 | 2.302591 |
| RP11-849H4.4 | -5.856196443 | CRTC1 | -1.88865 |
| AC093673.5 | 2.234154931 | AHR | 3.328529 |
| RP11-568N6.1 | 3.79276003 | TNFSF8 | 2.239707 |
| RP5-827C21.4 | 2.037414441 | EDF1 | 1.749313 |
| SNHG6 | 2.264283447 | EDF1 | 1.749313 |
| TRIM52-AS1 | 1.766004015 | EDF1 | 1.749313 |
| SNHG22 | 2.431449521 | EDF1 | 1.749313 |
| SNHG6 | 2.264283447 | RPL19 | 1.577028 |
| RP5-1142A6.2 | -4.912363938 | ENO3 | -2.5234 |
| BIRC6-AS1 | -6.422835412 | ENO3 | -2.5234 |
| AC004906.3 | -6.728177094 | ENO3 | -2.5234 |
| AC009133.12 | -2.710668063 | ENO3 | -2.5234 |
| SMARCA5-AS1 | -3.980253857 | ENO3 | -2.5234 |
| AC005329.7 | -3.477566523 | ENO3 | -2.5234 |
| RP11-849H4.4 | -5.856196443 | ENO3 | -2.5234 |
| AP006621.8 | -7.320584436 | ENO3 | -2.5234 |
| RP11-983P16.4 | -3.328470182 | ENO3 | -2.5234 |
| RP11-317G6.1 | -6.851917437 | ENO3 | -2.5234 |
| AC009133.15 | -6.077182664 | ENO3 | -2.5234 |
| AC027601.1 | -4.133297108 | ENO3 | -2.5234 |
| LA16c-358B7.3 | -7.46151727 | ENO3 | -2.5234 |
| LA16c-390E6.5 | -6.923175378 | ENO3 | -2.5234 |
| RP11-498C9.3 | -6.24175772 | ENO3 | -2.5234 |
| AC010761.8 | -8.159342974 | ENO3 | -2.5234 |
| RP11-452I5.2 | -5.672899155 | ENO3 | -2.5234 |
| CTB-31O20.4 | -7.245046692 | ENO3 | -2.5234 |
| AC005944.2 | -9.087897143 | ENO3 | -2.5234 |
| CTD-3193O13.13 | -6.219459245 | ENO3 | -2.5234 |
| LLNLR-284B4.1 | -8.114263599 | ENO3 | -2.5234 |
| CTB-58E17.9 | -7.157637637 | ENO3 | -2.5234 |
| LLNLR-307A6.1 | -6.307681579 | ENO3 | -2.5234 |
| CTBP1-AS | -7.633444677 | ENO3 | -2.5234 |
| AC133644.2 | 1.609914516 | CCL7 | 10.90523 |
| RP11-408H1.3 | 4.076872264 | CCL8 | 6.956092 |
| RP11-434D11.4 | 4.059155405 | CCL8 | 6.956092 |
| RP11-249C24.10 | 6.821082791 | CCL8 | 6.956092 |
| RP11-480C16.1 | 3.979218342 | CCL8 | 6.956092 |
| SNHG15 | 1.921718711 | ZNF330 | 1.547377 |
| RP11-334J6.7 | -5.19554326 | SH3D19 | -6.21829 |
| RP5-1142A6.2 | -4.912363938 | SH3D19 | -6.21829 |
| BIRC6-AS1 | -6.422835412 | SH3D19 | -6.21829 |
| SEMA3F-AS1 | -7.46960582 | SH3D19 | -6.21829 |
| AC009133.12 | -2.710668063 | SH3D19 | -6.21829 |
| KB-1208A12.3 | -8.38024941 | SH3D19 | -6.21829 |
| RP11-849H4.4 | -5.856196443 | SH3D19 | -6.21829 |
| AP006621.8 | -7.320584436 | SH3D19 | -6.21829 |
| RP11-983P16.4 | -3.328470182 | SH3D19 | -6.21829 |
| LA16c-358B7.3 | -7.46151727 | SH3D19 | -6.21829 |
| AC010761.8 | -8.159342974 | SH3D19 | -6.21829 |
| AC005944.2 | -9.087897143 | SH3D19 | -6.21829 |
| RP11-290D2.6 | -7.232193062 | SH3D19 | -6.21829 |
| RP4-742C19.13 | -7.615473666 | SH3D19 | -6.21829 |
| XX-FW83563B9.5 | -3.490321579 | SH3D19 | -6.21829 |
| CTBP1-AS | -7.633444677 | SH3D19 | -6.21829 |
| AC083884.8 | -6.310856242 | UPK2 | -3.63388 |
| RP11-455F5.3 | 2.064181782 | MANSC1 | 2.203197 |
| RP11-290D2.6 | -7.232193062 | ATN1 | -1.8952 |
| SNHG22 | 2.431449521 | COX6A1 | 1.5758 |
| RP11-321E2.4 | 3.793370281 | MAK | 1.570427 |
| RP11-256L6.3 | 2.392656028 | MAK | 1.570427 |
| LINC01270 | 3.497374777 | SLC26A8 | 1.574804 |
| RP11-321E2.4 | 3.793370281 | SLC26A8 | 1.574804 |
| LINC00936 | 4.057972374 | CD83 | 3.82006 |
| RP11-321E2.4 | 3.793370281 | VNN2 | 1.55146 |
| RP11-414H23.3 | 2.563956761 | VNN2 | 1.55146 |
| RP11-256L6.3 | 2.392656028 | VNN2 | 1.55146 |
| RP11-321E2.4 | 3.793370281 | BTNL8 | 4.877516 |
| RP11-455F5.3 | 2.064181782 | BTNL8 | 4.877516 |
| SNHG6 | 2.264283447 | RPL24 | 1.693667 |
| TRIM52-AS1 | 1.766004015 | RPL24 | 1.693667 |
| SNHG9 | 2.552371517 | RPL24 | 1.693667 |
| SNHG6 | 2.264283447 | IFT57 | 1.508511 |
| SMG7-AS1 | 2.208511128 | IFIH1 | 2.407889 |
| RP13-314C10.5 | 4.024098168 | IFIH1 | 2.407889 |
| RP11-434D11.4 | 4.059155405 | IFIH1 | 2.407889 |
| AP000640.2 | 2.814628078 | IFIH1 | 2.407889 |
| ARHGAP31-AS1 | 2.853994357 | ID2 | 2.181384 |
| SNHG6 | 2.264283447 | RPL22 | 1.970604 |
| RP11-439L18.1 | 2.967498008 | RNF19B | 2.712806 |
| SNHG6 | 2.264283447 | GADD45A | 1.948914 |
| RP5-827C21.4 | 2.037414441 | RPF1 | 1.725863 |
| SNHG6 | 2.264283447 | RPF1 | 1.725863 |
| TRIM52-AS1 | 1.766004015 | RPF1 | 1.725863 |
| SNHG22 | 2.431449521 | RPF1 | 1.725863 |
| RP11-408H1.3 | 4.076872264 | GBP1 | 3.738102 |
| AF127936.5 | 1.968152446 | GBP1 | 3.738102 |
| SMG7-AS1 | 2.208511128 | GBP1 | 3.738102 |
| RP13-314C10.5 | 4.024098168 | GBP1 | 3.738102 |
| RP11-439A17.10 | 3.620850282 | GBP1 | 3.738102 |
| RP11-434D11.4 | 4.059155405 | GBP1 | 3.738102 |
| RP11-44K6.2 | 4.690661249 | GBP1 | 3.738102 |
| RP11-480C16.1 | 3.979218342 | GBP1 | 3.738102 |
| SNHG6 | 2.264283447 | SYF2 | 1.916052 |
| SCARNA9 | 2.710268548 | SYF2 | 1.916052 |
| RP11-408H1.3 | 4.076872264 | IFIT3 | 5.079023 |
| SMG7-AS1 | 2.208511128 | IFIT3 | 5.079023 |
| RP11-439A17.10 | 3.620850282 | IFIT3 | 5.079023 |
| RP11-434D11.4 | 4.059155405 | IFIT3 | 5.079023 |
| RP11-44K6.2 | 4.690661249 | IFIT3 | 5.079023 |
| RP11-480C16.1 | 3.979218342 | IFIT3 | 5.079023 |
| RP11-408H1.3 | 4.076872264 | IFIT2 | 5.004913 |
| AF127936.5 | 1.968152446 | IFIT2 | 5.004913 |
| SMG7-AS1 | 2.208511128 | IFIT2 | 5.004913 |
| RP11-439A17.10 | 3.620850282 | IFIT2 | 5.004913 |
| RP11-434D11.4 | 4.059155405 | IFIT2 | 5.004913 |
| RP11-44K6.2 | 4.690661249 | IFIT2 | 5.004913 |
| RP11-480C16.1 | 3.979218342 | IFIT2 | 5.004913 |
| RP11-434D11.4 | 4.059155405 | CD274 | 3.438564 |
| RP11-249C24.10 | 6.821082791 | CD274 | 3.438564 |
| RP11-34F20.7 | 1.864892121 | TEX14 | 2.79945 |
| RP11-408H1.3 | 4.076872264 | TNFSF10 | 1.534629 |
| RP11-439A17.10 | 3.620850282 | TNFSF10 | 1.534629 |
| RP11-434D11.4 | 4.059155405 | TNFSF10 | 1.534629 |
| RP11-44K6.2 | 4.690661249 | TNFSF10 | 1.534629 |
| RP11-480C16.1 | 3.979218342 | TNFSF10 | 1.534629 |
| SNHG6 | 2.264283447 | RPL21 | 1.511982 |
| SNHG6 | 2.264283447 | PFDN5 | 1.63209 |
| CTA-29F11.1 | 4.39801455 | PFDN5 | 1.63209 |
| RP11-439L18.1 | 2.967498008 | BATF3 | 3.952432 |
| RP11-338C15.3 | 3.764806207 | BATF3 | 3.952432 |
| CFAP58-AS1 | 4.836828317 | ZNFX1 | 1.916322 |
| AC093673.5 | 2.234154931 | PTGER2 | 2.059663 |
| AP000355.2 | 4.367811973 | IL1B | 3.748871 |
| SNHG6 | 2.264283447 | RPL23 | 1.961514 |
| SERPINB9P1 | 1.994995743 | TRIP10 | 3.961495 |
| SCARNA9 | 2.710268548 | COX6B1 | 2.492219 |
| SNHG22 | 2.431449521 | COX6B1 | 2.492219 |
| SNHG22 | 2.431449521 | PRDX5 | 2.016889 |
| AC004906.3 | -6.728177094 | PRR12 | -2.50286 |
| AC009133.12 | -2.710668063 | PRR12 | -2.50286 |
| AC005329.7 | -3.477566523 | PRR12 | -2.50286 |
| RP11-849H4.4 | -5.856196443 | PRR12 | -2.50286 |
| AC009133.15 | -6.077182664 | PRR12 | -2.50286 |
| LA16c-358B7.3 | -7.46151727 | PRR12 | -2.50286 |
| AC005944.2 | -9.087897143 | PRR12 | -2.50286 |
| CTD-3193O13.13 | -6.219459245 | PRR12 | -2.50286 |
| AC005785.2 | -3.612329917 | PRR12 | -2.50286 |
| LLNLR-284B4.1 | -8.114263599 | PRR12 | -2.50286 |
| CTB-58E17.9 | -7.157637637 | PRR12 | -2.50286 |
| CTBP1-AS | -7.633444677 | PRR12 | -2.50286 |
| SNHG6 | 2.264283447 | SBDS | 1.6972 |
| SNHG9 | 2.552371517 | SBDS | 1.6972 |
| RP1-292L20.3 | 3.477115478 | HNRNPH2 | 2.017449 |
| SNHG15 | 1.921718711 | ATF4 | 1.660442 |
| CTA-29F11.1 | 4.39801455 | ATF4 | 1.660442 |
| DHRSX-IT1 | -2.343412267 | CBFA2T3 | -1.91264 |
| SNHG6 | 2.264283447 | RPL36 | 2.017594 |
| RP11-408H1.3 | 4.076872264 | IDO1 | 3.615498 |
| AF127936.5 | 1.968152446 | IDO1 | 3.615498 |
| SMG7-AS1 | 2.208511128 | IDO1 | 3.615498 |
| RP11-439A17.10 | 3.620850282 | IDO1 | 3.615498 |
| RP11-434D11.4 | 4.059155405 | IDO1 | 3.615498 |
| RP11-44K6.2 | 4.690661249 | IDO1 | 3.615498 |
| RP11-480C16.1 | 3.979218342 | IDO1 | 3.615498 |
| SNHG6 | 2.264283447 | RPL27 | 3.117986 |
| TRIM52-AS1 | 1.766004015 | RPL27 | 3.117986 |
| SNHG9 | 2.552371517 | RPL27 | 3.117986 |
| RP11-439L18.1 | 2.967498008 | GCH1 | 2.066712 |
| RP11-568N6.1 | 3.79276003 | RIN2 | 4.102778 |
| RP5-827C21.4 | 2.037414441 | CCDC59 | 1.694115 |
| SNHG6 | 2.264283447 | CCDC59 | 1.694115 |
| TRIM52-AS1 | 1.766004015 | CCDC59 | 1.694115 |
| LINC01506 | 2.456792493 | LYVE1 | 1.700941 |
| RP11-321E2.4 | 3.793370281 | LYVE1 | 1.700941 |
| RP11-455F5.3 | 2.064181782 | LYVE1 | 1.700941 |
| RP11-519G16.3 | 3.630476003 | IRAK2 | 3.869901 |
| AC012363.4 | 1.770431816 | RSAD2 | 3.478933 |
| RP11-326C3.13 | 3.42478901 | RSAD2 | 3.478933 |
| SNHG6 | 2.264283447 | RPS15A | 3.194872 |
| SNHG9 | 2.552371517 | RPS15A | 3.194872 |
| AP000355.2 | 4.367811973 | DRAM1 | 3.422132 |
| KCNJ2-AS1 | 4.898890152 | DRAM1 | 3.422132 |
| AC002480.2 | 1.660993022 | IL6 | 10.36919 |
| SNHG6 | 2.264283447 | ATP6V1G1 | 1.692979 |
| TRIM52-AS1 | 1.766004015 | ATP6V1G1 | 1.692979 |
| CTA-29F11.1 | 4.39801455 | ATP6V1G1 | 1.692979 |
| SNHG6 | 2.264283447 | RPL35 | 1.516157 |
| SNHG15 | 1.921718711 | PIM1 | 1.921265 |
| AC091878.1 | 2.191714958 | KIAA0319 | 1.543611 |
| RP11-624L4.1 | 3.51265333 | THBS1 | 3.617571 |
| CTD-2033D15.2 | 3.213885297 | THBS1 | 3.617571 |
| RP11-568N6.1 | 3.79276003 | DUSP5 | 2.50873 |
| SNHG6 | 2.264283447 | RPS24 | 2.676298 |
| TRIM52-AS1 | 1.766004015 | RPS24 | 2.676298 |
| RP11-408H1.3 | 4.076872264 | HERC5 | 2.244222 |
| RP11-439A17.10 | 3.620850282 | HERC5 | 2.244222 |
| RP11-434D11.4 | 4.059155405 | HERC5 | 2.244222 |
| RP11-44K6.2 | 4.690661249 | HERC5 | 2.244222 |
| RP11-480C16.1 | 3.979218342 | HERC5 | 2.244222 |
| RP11-408H1.3 | 4.076872264 | CXCL9 | 2.749794 |
| AF127936.5 | 1.968152446 | CXCL9 | 2.749794 |
| RP11-439A17.10 | 3.620850282 | CXCL9 | 2.749794 |
| RP11-434D11.4 | 4.059155405 | CXCL9 | 2.749794 |
| RP11-44K6.2 | 4.690661249 | CXCL9 | 2.749794 |
| RP11-480C16.1 | 3.979218342 | CXCL9 | 2.749794 |
| LINC01010 | 5.371338609 | PHLDA1 | 3.79879 |
| LINC01010 | 5.371338609 | GAS2L3 | 2.58775 |
| MIR222HG | 4.018234246 | GPR84 | 3.876621 |
| CTD-2020K17.3 | -5.184076698 | SETD1B | -1.88801 |
| RP11-434D11.4 | 4.059155405 | WARS | 2.209676 |
| RP5-827C21.4 | 2.037414441 | SRP14 | 1.808428 |
| SNHG6 | 2.264283447 | SRP14 | 1.808428 |
| TRIM52-AS1 | 1.766004015 | SRP14 | 1.808428 |
| CFAP58-AS1 | 4.836828317 | BCL2A1 | 5.342016 |
| AC004906.3 | -6.728177094 | TPGS1 | -1.51552 |
| CD81-AS1 | -4.434622559 | FAM71E1 | -5.04396 |
| RP5-827C21.4 | 2.037414441 | RPS11 | 2.453259 |
| SNHG6 | 2.264283447 | RPS11 | 2.453259 |
| TRIM52-AS1 | 1.766004015 | RPS11 | 2.453259 |
| SNHG9 | 2.552371517 | RPS11 | 2.453259 |
| SNHG6 | 2.264283447 | RPL11 | 2.733463 |
| SNHG9 | 2.552371517 | RPL11 | 2.733463 |
| RAP2C-AS1 | 1.66407898 | TP53BP2 | 1.840565 |
| SNHG6 | 2.264283447 | RPL32 | 1.592464 |
| RP11-73E17.2 | 1.606414101 | NFKBIZ | 2.394568 |
| CTB-41I6.1 | 2.454428789 | NFKBIZ | 2.394568 |
| CTA-293F17.1 | 5.144647305 | PLA1A | 4.406905 |
| LINC00936 | 4.057972374 | ADPRH | 1.890398 |
| RP11-849H4.4 | -5.856196443 | SLC26A1 | -4.13551 |
| SNHG6 | 2.264283447 | RPL37 | 2.535515 |
| TRIM52-AS1 | 1.766004015 | RPL37 | 2.535515 |
| MIR222HG | 4.018234246 | FEM1C | 1.596985 |
| RP5-827C21.4 | 2.037414441 | POLR2K | 1.50152 |
| TRIM52-AS1 | 1.766004015 | POLR2K | 1.50152 |
| CTA-293F17.1 | 5.144647305 | CDKN2B | 2.518635 |
| SNHG6 | 2.264283447 | FAU | 2.018714 |
| SCARNA9 | 2.710268548 | FAU | 2.018714 |
| SNHG9 | 2.552371517 | FAU | 2.018714 |
| CTA-29F11.1 | 4.39801455 | FAU | 2.018714 |
| RP5-827C21.4 | 2.037414441 | SAP18 | 1.554477 |
| SCARNA9 | 2.710268548 | SAP18 | 1.554477 |
| SNHG22 | 2.431449521 | SAP18 | 1.554477 |
| RAP2C-AS1 | 1.66407898 | NOCT | 2.604515 |
| ZEB2-AS1 | 2.447879212 | NOCT | 2.604515 |
| RP1-239B22.5 | 2.122078941 | NOCT | 2.604515 |
| RP11-351I24.1 | 3.420472032 | ACSL1 | 3.949107 |
| RP11-334J6.7 | -5.19554326 | ATG10 | -1.82472 |
| RP5-1142A6.2 | -4.912363938 | ATG10 | -1.82472 |
| AC005540.3 | -3.373016714 | ATG10 | -1.82472 |
| AC004906.3 | -6.728177094 | ATG10 | -1.82472 |
| AC009133.12 | -2.710668063 | ATG10 | -1.82472 |
| SMARCA5-AS1 | -3.980253857 | ATG10 | -1.82472 |
| KB-1208A12.3 | -8.38024941 | ATG10 | -1.82472 |
| RP11-849H4.4 | -5.856196443 | ATG10 | -1.82472 |
| RP11-106M3.3 | -6.043017396 | ATG10 | -1.82472 |
| LA16c-358B7.3 | -7.46151727 | ATG10 | -1.82472 |
| AC010761.8 | -8.159342974 | ATG10 | -1.82472 |
| RP11-138I1.4 | -9.392962887 | ATG10 | -1.82472 |
| RP11-258F1.1 | -2.502340081 | ATG10 | -1.82472 |
| AC005944.2 | -9.087897143 | ATG10 | -1.82472 |
| CTD-3193O13.13 | -6.219459245 | ATG10 | -1.82472 |
| LLNLR-284B4.1 | -8.114263599 | ATG10 | -1.82472 |
| XX-FW83563B9.5 | -3.490321579 | ATG10 | -1.82472 |
| CTBP1-AS | -7.633444677 | ATG10 | -1.82472 |
| MYCBP2-AS1 | -4.695295094 | FARP1 | -4.90716 |
| AC012363.4 | 1.770431816 | GBP5 | 2.584043 |
| RP13-314C10.5 | 4.024098168 | GBP5 | 2.584043 |
| RP11-434D11.4 | 4.059155405 | GBP5 | 2.584043 |
| RP11-380G5.2 | 1.788256123 | EPHB1 | 2.769538 |
| LINC01506 | 2.456792493 | EPHB1 | 2.769538 |
| AF124730.4 | 4.91345098 | SLC16A1 | 1.648087 |
| SNHG6 | 2.264283447 | UQCRB | 2.364326 |
| TRIM52-AS1 | 1.766004015 | UQCRB | 2.364326 |
| SNHG6 | 2.264283447 | RPL30 | 1.754512 |
| SNHG9 | 2.552371517 | RPL30 | 1.754512 |
| RP11-380G5.2 | 1.788256123 | KCNJ15 | 4.627149 |
| RP11-63N8.3 | 4.208438062 | KCNJ15 | 4.627149 |
| RP11-321E2.4 | 3.793370281 | KCNJ15 | 4.627149 |
| AC091878.1 | 2.191714958 | GALNT14 | 1.762898 |
| AC005329.7 | -3.477566523 | C5orf45 | -1.71292 |
| RP4-751H13.7 | -3.08936898 | C5orf45 | -1.71292 |
| SMARCA5-AS1 | -3.980253857 | GRIN2C | -6.27101 |
| AC005944.2 | -9.087897143 | GRIN2C | -6.27101 |
| SERPINB9P1 | 1.994995743 | AK4 | 5.684791 |
| KLF3-AS1 | -2.743200165 | MXRA8 | -3.34916 |
| CFAP58-AS1 | 4.836828317 | GBP2 | 3.197203 |
| RP11-380G5.2 | 1.788256123 | FCGR3B | 5.644662 |
| LINC01506 | 2.456792493 | FCGR3B | 5.644662 |
| RP11-249C24.10 | 6.821082791 | FAM71A | 5.163664 |
| RP11-321E2.4 | 3.793370281 | CXCR1 | 2.095963 |
| SNHG22 | 2.431449521 | HMGB2 | 2.117585 |
| SMG7-AS1 | 2.208511128 | RANBP3L | 4.024992 |
| RP11-21C4.1 | 4.096140986 | RANBP3L | 4.024992 |
| SNHG6 | 2.264283447 | NSA2 | 1.790586 |
| TRIM52-AS1 | 1.766004015 | NSA2 | 1.790586 |
| SNHG6 | 2.264283447 | RPL36AL | 3.28701 |
| SCARNA9 | 2.710268548 | RPL36AL | 3.28701 |
| SNHG22 | 2.431449521 | RPL36AL | 3.28701 |
| RP11-408H1.3 | 4.076872264 | ZNF503 | 1.539314 |
| RP11-249C24.10 | 6.821082791 | ZNF503 | 1.539314 |
| SNHG15 | 1.921718711 | DDX21 | 2.100276 |
| SNHG6 | 2.264283447 | RPL27A | 2.053535 |
| TRIM52-AS1 | 1.766004015 | RPL27A | 2.053535 |
| RP11-568N6.1 | 3.79276003 | C15orf48 | 5.783125 |
| SNHG6 | 2.264283447 | ATP5L | 1.964712 |
| TRIM52-AS1 | 1.766004015 | ATP5L | 1.964712 |
| CTD-2013N24.2 | -4.095315846 | JSRP1 | -4.84747 |
| PXN-AS1 | -2.634689192 | JSRP1 | -4.84747 |
| RP5-1142A6.5 | -5.73662872 | JSRP1 | -4.84747 |
| CTD-2231E14.8 | -6.052319332 | JSRP1 | -4.84747 |
| RP11-290D2.6 | -7.232193062 | JSRP1 | -4.84747 |
| SPACA6 | 2.335156664 | PLIN4 | 2.54801 |
| RP5-827C21.4 | 2.037414441 | HIST1H1E | 2.944715 |
| TRIM52-AS1 | 1.766004015 | HIST1H1E | 2.944715 |
| SNHG22 | 2.431449521 | HIST1H1E | 2.944715 |
| SNHG6 | 2.264283447 | NDUFS5 | 2.522283 |
| SNHG22 | 2.431449521 | ATP5I | 1.737203 |
| RP11-408H1.3 | 4.076872264 | CXCL10 | 6.157102 |
| AF127936.5 | 1.968152446 | CXCL10 | 6.157102 |
| SMG7-AS1 | 2.208511128 | CXCL10 | 6.157102 |
| RP11-439A17.10 | 3.620850282 | CXCL10 | 6.157102 |
| RP11-434D11.4 | 4.059155405 | CXCL10 | 6.157102 |
| RP11-44K6.2 | 4.690661249 | CXCL10 | 6.157102 |
| RP11-249C24.10 | 6.821082791 | CXCL10 | 6.157102 |
| RP11-480C16.1 | 3.979218342 | CXCL10 | 6.157102 |
| RP11-408H1.3 | 4.076872264 | CXCL11 | 6.07574 |
| AF127936.5 | 1.968152446 | CXCL11 | 6.07574 |
| SMG7-AS1 | 2.208511128 | CXCL11 | 6.07574 |
| RP11-439A17.10 | 3.620850282 | CXCL11 | 6.07574 |
| RP11-434D11.4 | 4.059155405 | CXCL11 | 6.07574 |
| RP11-44K6.2 | 4.690661249 | CXCL11 | 6.07574 |
| RP11-249C24.10 | 6.821082791 | CXCL11 | 6.07574 |
| RP11-480C16.1 | 3.979218342 | CXCL11 | 6.07574 |
| RP5-827C21.4 | 2.037414441 | FAM103A1 | 1.554535 |
| TRIM52-AS1 | 1.766004015 | FAM103A1 | 1.554535 |
| SNHG22 | 2.431449521 | FAM103A1 | 1.554535 |
| RP11-849H4.4 | -5.856196443 | SLFNL1 | -3.30917 |
| AC009133.15 | -6.077182664 | SLFNL1 | -3.30917 |
| AC027601.1 | -4.133297108 | SLFNL1 | -3.30917 |
| CTB-31O20.4 | -7.245046692 | SLFNL1 | -3.30917 |
| AC005944.2 | -9.087897143 | SLFNL1 | -3.30917 |
| LLNLR-284B4.1 | -8.114263599 | SLFNL1 | -3.30917 |
| CTBP1-AS | -7.633444677 | SLFNL1 | -3.30917 |
| RP11-408H1.3 | 4.076872264 | TMEM217 | 1.838095 |
| AF127936.5 | 1.968152446 | TMEM217 | 1.838095 |
| SMG7-AS1 | 2.208511128 | TMEM217 | 1.838095 |
| RP11-439A17.10 | 3.620850282 | TMEM217 | 1.838095 |
| RP11-434D11.4 | 4.059155405 | TMEM217 | 1.838095 |
| RP11-44K6.2 | 4.690661249 | TMEM217 | 1.838095 |
| RP11-249C24.10 | 6.821082791 | TMEM217 | 1.838095 |
| RP11-480C16.1 | 3.979218342 | TMEM217 | 1.838095 |
| C5orf66 | -6.657273334 | SNX32 | -5.63856 |
| CTD-2020K17.3 | -5.184076698 | SNX32 | -5.63856 |
| AC002401.1 | -5.171605194 | SNX32 | -5.63856 |
| PXN-AS1 | -2.634689192 | SNX32 | -5.63856 |
| RP5-940J5.9 | -6.467710862 | SNX32 | -5.63856 |
| SNHG6 | 2.264283447 | RPL38 | 2.152269 |
| RP5-827C21.4 | 2.037414441 | TRMT112 | 1.584272 |
| SNHG6 | 2.264283447 | TRMT112 | 1.584272 |
| SCARNA9 | 2.710268548 | TRMT112 | 1.584272 |
| SNHG22 | 2.431449521 | TRMT112 | 1.584272 |
| MIR22HG | 2.585920544 | TRIB1 | 3.497344 |
| RP11-561B11.3 | 4.66325723 | THAP2 | 2.594195 |
| RP11-380G5.2 | 1.788256123 | TNFRSF10C | 2.672254 |
| RP11-321E2.4 | 3.793370281 | TNFRSF10C | 2.672254 |
| RP11-414H23.3 | 2.563956761 | TNFRSF10C | 2.672254 |
| RP11-256L6.3 | 2.392656028 | TNFRSF10C | 2.672254 |
| SNHG6 | 2.264283447 | LINC00116 | 2.077376 |
| TRIM52-AS1 | 1.766004015 | LINC00116 | 2.077376 |
| LINC00612 | -6.843729127 | A2M | -3.65166 |
| RP11-290L1.2 | 3.639085712 | TUBB6 | 2.471962 |
| SCARNA9 | 2.710268548 | COX8A | 2.840229 |
| CTA-29F11.1 | 4.39801455 | SVBP | 1.794387 |
| SNHG6 | 2.264283447 | RPS27 | 3.878032 |
| TRIM52-AS1 | 1.766004015 | RPS27 | 3.878032 |
| CTA-293F17.1 | 5.144647305 | MSC | 3.327811 |
| RP11-408H1.3 | 4.076872264 | ERICH3 | 1.825814 |
| AF127936.5 | 1.968152446 | ERICH3 | 1.825814 |
| RP11-439A17.10 | 3.620850282 | ERICH3 | 1.825814 |
| RP11-434D11.4 | 4.059155405 | ERICH3 | 1.825814 |
| RP11-44K6.2 | 4.690661249 | ERICH3 | 1.825814 |
| RP11-480C16.1 | 3.979218342 | ERICH3 | 1.825814 |
| LINC01506 | 2.456792493 | CXCR2 | 2.933845 |
| RP11-321E2.4 | 3.793370281 | CXCR2 | 2.933845 |
| RP11-455F5.3 | 2.064181782 | CXCR2 | 2.933845 |
| RP11-256L6.3 | 2.392656028 | CXCR2 | 2.933845 |
| AC008984.2 | 2.828915738 | SIAH2 | 1.503148 |
| STK24-AS1 | 1.778604657 | ANXA2 | 1.745293 |
| CTD-2530H12.2 | 1.914535634 | ADGRG3 | 2.476651 |
| SNHG6 | 2.264283447 | RPL35A | 1.654798 |
| TRIM52-AS1 | 1.766004015 | RPL35A | 1.654798 |
| RP5-827C21.4 | 2.037414441 | HIST2H2AC | 2.228327 |
| SNHG22 | 2.431449521 | HIST2H2AC | 2.228327 |
| RP5-827C21.4 | 2.037414441 | HIST2H2AB | 2.26405 |
| SNHG22 | 2.431449521 | HIST2H2AB | 2.26405 |
| SRGAP2-AS1 | 4.009973092 | PRR16 | 4.118179 |
| RP11-338C15.3 | 3.764806207 | PRR16 | 4.118179 |
| RP11-568N6.1 | 3.79276003 | PRR16 | 4.118179 |
| CTA-29F11.1 | 4.39801455 | NDUFA6 | 1.871131 |
| AC004906.3 | -6.728177094 | BCL9L | -1.94826 |
| RP11-849H4.4 | -5.856196443 | BCL9L | -1.94826 |
| AC009133.15 | -6.077182664 | BCL9L | -1.94826 |
| AC010761.8 | -8.159342974 | BCL9L | -1.94826 |
| AC005944.2 | -9.087897143 | BCL9L | -1.94826 |
| AC005785.2 | -3.612329917 | BCL9L | -1.94826 |
| LLNLR-284B4.1 | -8.114263599 | BCL9L | -1.94826 |
| CTB-58E17.9 | -7.157637637 | BCL9L | -1.94826 |
| CTB-61M7.2 | 3.116476162 | FCAR | 3.113203 |
| RP5-1142A6.2 | -4.912363938 | ZACN | -5.31079 |
| BIRC6-AS1 | -6.422835412 | ZACN | -5.31079 |
| AC009133.12 | -2.710668063 | ZACN | -5.31079 |
| RP11-849H4.4 | -5.856196443 | ZACN | -5.31079 |
| AP006621.8 | -7.320584436 | ZACN | -5.31079 |
| RP11-317G6.1 | -6.851917437 | ZACN | -5.31079 |
| AC009133.15 | -6.077182664 | ZACN | -5.31079 |
| AC027601.1 | -4.133297108 | ZACN | -5.31079 |
| LA16c-358B7.3 | -7.46151727 | ZACN | -5.31079 |
| LA16c-390E6.5 | -6.923175378 | ZACN | -5.31079 |
| RP11-498C9.3 | -6.24175772 | ZACN | -5.31079 |
| RP11-452I5.2 | -5.672899155 | ZACN | -5.31079 |
| CTB-31O20.4 | -7.245046692 | ZACN | -5.31079 |
| AC005944.2 | -9.087897143 | ZACN | -5.31079 |
| CTD-3193O13.13 | -6.219459245 | ZACN | -5.31079 |
| CTD-2231E14.8 | -6.052319332 | ZACN | -5.31079 |
| RP11-290D2.6 | -7.232193062 | ZACN | -5.31079 |
| LLNLR-284B4.1 | -8.114263599 | ZACN | -5.31079 |
| CTB-58E17.9 | -7.157637637 | ZACN | -5.31079 |
| RP11-12J10.4 | -3.108590725 | ZACN | -5.31079 |
| LLNLR-307A6.1 | -6.307681579 | ZACN | -5.31079 |
| CTBP1-AS | -7.633444677 | ZACN | -5.31079 |
| SNHG6 | 2.264283447 | PTMA | 1.685316 |
| TRIM52-AS1 | 1.766004015 | PTMA | 1.685316 |
| RP5-827C21.4 | 2.037414441 | HIST1H1C | 2.576142 |
| TRIM52-AS1 | 1.766004015 | HIST1H1C | 2.576142 |
| RP1-239B22.5 | 2.122078941 | NCR3LG1 | 2.040948 |
| RP11-249C24.10 | 6.821082791 | FAM72B | 4.524136 |
| RP5-1142A6.2 | -4.912363938 | TMEM120B | -1.8523 |
| RP11-849H4.4 | -5.856196443 | TMEM120B | -1.8523 |
| RP11-983P16.4 | -3.328470182 | TMEM120B | -1.8523 |
| RP11-24N18.1 | -6.09320084 | TMEM120B | -1.8523 |
| LA16c-358B7.3 | -7.46151727 | TMEM120B | -1.8523 |
| LINC01270 | 3.497374777 | IL1RAP | 1.686412 |
| RP5-968J1.1 | 1.798558211 | MME | 4.017327 |
| SRGAP2-AS1 | 4.009973092 | FAM72A | 3.574209 |
| RP11-249C24.10 | 6.821082791 | FAM72A | 3.574209 |
| RP5-827C21.4 | 2.037414441 | HIST1H4C | 2.285206 |
| SNHG22 | 2.431449521 | HIST1H4C | 2.285206 |
| RP11-408H1.3 | 4.076872264 | IL27 | 2.019376 |
| AF127936.5 | 1.968152446 | IL27 | 2.019376 |
| RP11-434D11.4 | 4.059155405 | IL27 | 2.019376 |
| RP11-44K6.2 | 4.690661249 | IL27 | 2.019376 |
| RP11-480C16.1 | 3.979218342 | IL27 | 2.019376 |
| RP11-258F1.1 | -2.502340081 | ZNF628 | -2.06633 |
| SNHG6 | 2.264283447 | RPL37A | 2.399874 |
| TRIM52-AS1 | 1.766004015 | RPL37A | 2.399874 |
| SNHG22 | 2.431449521 | HIST1H2BK | 1.908856 |
| SNHG6 | 2.264283447 | RPL12 | 2.172223 |
| RP11-568N6.1 | 3.79276003 | C1orf122 | 1.792995 |
| SNHG6 | 2.264283447 | RPL23A | 2.089988 |
| TRIM52-AS1 | 1.766004015 | RPL23A | 2.089988 |
| SNHG9 | 2.552371517 | RPL23A | 2.089988 |
| RP11-242C19.2 | 1.725262211 | GK | 1.818477 |
| RP11-153M7.5 | 3.357032402 | GK | 1.818477 |
| SNHG6 | 2.264283447 | MT-CO3 | 2.312927 |
| CTA-29F11.1 | 4.39801455 | MT-CO3 | 2.312927 |
| RP11-568N6.1 | 3.79276003 | CARD16 | 2.978538 |
| CTD-2020K17.3 | -5.184076698 | CCDC85C | -2.18752 |
| RP11-408H1.3 | 4.076872264 | FAM72D | 2.944562 |
| RP11-249C24.10 | 6.821082791 | FAM72D | 2.944562 |
| AP006621.8 | -7.320584436 | C1orf167 | -3.98877 |
| LA16c-358B7.3 | -7.46151727 | C1orf167 | -3.98877 |
| RP11-498C9.3 | -6.24175772 | C1orf167 | -3.98877 |
| AP006621.8 | -7.320584436 | LINGO3 | -5.03971 |
| RP5-827C21.4 | 2.037414441 | UBA52 | 2.189223 |
| SNHG6 | 2.264283447 | UBA52 | 2.189223 |
| TRIM52-AS1 | 1.766004015 | UBA52 | 2.189223 |
| SCARNA9 | 2.710268548 | UBA52 | 2.189223 |
| SNHG9 | 2.552371517 | UBA52 | 2.189223 |
| SNHG6 | 2.264283447 | RPL41 | 2.600045 |
| SNHG9 | 2.552371517 | RPL41 | 2.600045 |
| SNHG6 | 2.264283447 | RPS18 | 1.668983 |
| RP5-827C21.4 | 2.037414441 | TMA7 | 1.575074 |
| SNHG6 | 2.264283447 | TMA7 | 1.575074 |
| TRIM52-AS1 | 1.766004015 | TMA7 | 1.575074 |
| SNHG22 | 2.431449521 | TMA7 | 1.575074 |
| RP11-34F20.7 | 1.864892121 | TNF | 2.142718 |
| RP5-827C21.4 | 2.037414441 | HIST1H2BN | 2.263513 |
| SNHG22 | 2.431449521 | HIST1H2BN | 2.263513 |
| RP5-827C21.4 | 2.037414441 | MIF | 2.791618 |
| SNHG6 | 2.264283447 | MIF | 2.791618 |
| TRIM52-AS1 | 1.766004015 | MIF | 2.791618 |
| LLNLR-470E3.1 | 4.171299973 | SIGLEC14 | 5.080217 |
| SMG7-AS1 | 2.208511128 | CARD17 | 2.968618 |
| RP13-314C10.5 | 4.024098168 | CARD17 | 2.968618 |
| AP000640.2 | 2.814628078 | CARD17 | 2.968618 |
| RP5-968J1.1 | 1.798558211 | MGAM | 3.743434 |
| RP11-529H2.2 | -2.513680798 | C6orf229 | -2.05402 |
| DGCR12 | -1.57820432 | C6orf229 | -2.05402 |
| AP006621.8 | -7.320584436 | ZNF865 | -2.07769 |
| RP11-498C9.3 | -6.24175772 | ZNF865 | -2.07769 |
| SNHG6 | 2.264283447 | RPL17 | 1.540446 |
| AC093673.5 | 2.234154931 | C17orf96 | 3.040768 |
| RP5-827C21.4 | 2.037414441 | HIST1H2AK | 2.511415 |
| SNHG22 | 2.431449521 | HIST1H2AK | 2.511415 |
| LINC01093 | 6.129847371 | CCL4 | 4.037131 |
| KCNJ2-AS1 | 4.898890152 | CCL4 | 4.037131 |
| RP5-827C21.4 | 2.037414441 | HIST1H3A | 1.97248 |
| RP5-827C21.4 | 2.037414441 | HIST1H4E | 3.385708 |
| SNHG6 | 2.264283447 | HIST1H4E | 3.385708 |
| TRIM52-AS1 | 1.766004015 | HIST1H4E | 3.385708 |
| SNHG22 | 2.431449521 | HIST1H4E | 3.385708 |
| RP5-827C21.4 | 2.037414441 | HIST1H2AE | 2.772373 |
| SNHG22 | 2.431449521 | HIST1H2AE | 2.772373 |
| SNHG22 | 2.431449521 | HIST1H2BF | 1.983845 |
| RP4-742C19.13 | -7.615473666 | FP325331.1 | -5.10095 |
| RP11-849H4.4 | -5.856196443 | AC009065.4 | -6.83008 |
| RP11-867G23.4 | -6.243158252 | AC009065.4 | -6.83008 |
| RP11-106M3.3 | -6.043017396 | AC009065.4 | -6.83008 |
| CTD-2033A16.3 | -4.031081673 | AC009065.4 | -6.83008 |
| RP11-498C9.3 | -6.24175772 | AC009065.4 | -6.83008 |
| CTB-31O20.4 | -7.245046692 | AC009065.4 | -6.83008 |
| AC005944.2 | -9.087897143 | AC009065.4 | -6.83008 |
| CTBP1-AS | -7.633444677 | AC009065.4 | -6.83008 |
| CTD-2020K17.3 | -5.184076698 | AP000769.1 | -11.1524 |
| AC002401.1 | -5.171605194 | AP000769.1 | -11.1524 |
| PXN-AS1 | -2.634689192 | AP000769.1 | -11.1524 |
| RP1-56K13.3 | -7.033194947 | AP000769.1 | -11.1524 |

## Table S4. The lncRNA targets in the RP/HC group

| **Lnc_Name** | **Lnc_log2FC(RP/HC)** | **Gene_Name** | **Gene_log2FC(RP/HC)** |
| --- | --- | --- | --- |
| LINC01272 | -1.638723871 | MMP25 | -2.438293915 |
| LINC01001 | -2.273765643 | MMP25 | -2.438293915 |
| RP5-968J1.1 | -1.609094214 | MMP25 | -2.438293915 |
| RP11-34P13.13 | -1.776488582 | MMP25 | -2.438293915 |
| RP11-875O11.3 | -3.045134873 | MMP25 | -2.438293915 |
| RP11-25K21.6 | -2.749251115 | MMP25 | -2.438293915 |
| LINC01002 | -1.967031255 | MMP25 | -2.438293915 |
| RP11-482G13.1 | -2.287077989 | MMP25 | -2.438293915 |
| RP11-619I22.1 | -3.476162195 | EXTL3 | -1.525915908 |
| LINC01002 | -1.967031255 | SLC45A4 | -1.917010048 |
| RP11-482G13.1 | -2.287077989 | SLC45A4 | -1.917010048 |
| RP5-968J1.1 | -1.609094214 | DAPK2 | -2.088673474 |
| RP11-44F14.2 | -2.253372035 | DAPK2 | -2.088673474 |
| RP11-44F14.8 | -2.84244213 | DAPK2 | -2.088673474 |
| RP11-619I22.1 | -3.476162195 | DAPK2 | -2.088673474 |
| RP11-25K21.6 | -2.749251115 | DAPK2 | -2.088673474 |
| RP11-106M3.3 | -4.791958665 | FAM184B | -3.537721579 |
| RP5-1142A6.2 | -3.776570831 | NRIP2 | -4.419651801 |
| RP11-269F19.2 | -2.916843938 | NRIP2 | -4.419651801 |
| KB-1208A12.3 | -6.747493135 | NRIP2 | -4.419651801 |
| RP11-849H4.4 | -4.799792287 | NRIP2 | -4.419651801 |
| CTB-31O20.4 | -5.912842662 | NRIP2 | -4.419651801 |
| AC005944.2 | -7.516561983 | NRIP2 | -4.419651801 |
| LLNLR-284B4.1 | -6.420632191 | NRIP2 | -4.419651801 |
| CTBP1-AS | -6.439383626 | NRIP2 | -4.419651801 |
| RP11-568N6.1 | 1.793391075 | TRAF3IP2 | 2.287209797 |
| CTD-2020K17.3 | -4.744792149 | GLTSCR1 | -1.773256559 |
| RP11-849H4.4 | -4.799792287 | GLTSCR1 | -1.773256559 |
| AP006621.8 | -6.292200789 | GLTSCR1 | -1.773256559 |
| RP5-1142A6.5 | -5.121273913 | GLTSCR1 | -1.773256559 |
| LA16c-358B7.3 | -6.399909599 | GLTSCR1 | -1.773256559 |
| RP11-498C9.3 | -5.3947311 | GLTSCR1 | -1.773256559 |
| CTD-2231E14.8 | -5.249237907 | GLTSCR1 | -1.773256559 |
| SNHG6 | 2.864593326 | YBX1 | 2.488370975 |
| CTA-29F11.1 | 3.969693116 | YBX1 | 2.488370975 |
| RP11-529H2.2 | -3.977727445 | DGCR2 | -1.727218979 |
| CTB-152G17.6 | -1.969668991 | DGCR2 | -1.727218979 |
| DGCR11 | -1.972880324 | DGCR2 | -1.727218979 |
| DGCR12 | -2.230457715 | DGCR2 | -1.727218979 |
| LINC01002 | -1.967031255 | ST6GALNAC2 | -1.754045803 |
| RP11-482G13.1 | -2.287077989 | ST6GALNAC2 | -1.754045803 |
| EPB41L4A-AS1 | 1.764574685 | RPL31 | 2.185527925 |
| SNHG6 | 2.864593326 | RPL31 | 2.185527925 |
| RP11-290F5.2 | -2.567649204 | MRVI1 | -1.819000285 |
| LINC01359 | -2.371602714 | MRVI1 | -1.819000285 |
| RP11-321E2.4 | -2.040412365 | MRVI1 | -1.819000285 |
| RP11-414H23.3 | -2.418471674 | MRVI1 | -1.819000285 |
| RP11-619I22.1 | -3.476162195 | MRVI1 | -1.819000285 |
| LINC01359 | -2.371602714 | PPP1R12B | -1.564851448 |
| LINC01513 | -3.711658406 | PPP1R12B | -1.564851448 |
| RP11-290F5.1 | -2.675904236 | PPP1R12B | -1.564851448 |
| RP11-529H2.2 | -3.977727445 | PPP1R12B | -1.564851448 |
| RP11-44F14.2 | -2.253372035 | PPP1R12B | -1.564851448 |
| RP11-68I3.11 | -2.095759421 | PPP1R12B | -1.564851448 |
| RP11-619I22.1 | -3.476162195 | PPP1R12B | -1.564851448 |
| RP11-25K21.6 | -2.749251115 | PPP1R12B | -1.564851448 |
| RP11-321E2.4 | -2.040412365 | HAL | -1.588015222 |
| RP11-875O11.3 | -3.045134873 | HAL | -1.588015222 |
| RP11-256L6.3 | -1.696848309 | HAL | -1.588015222 |
| RP11-619I22.1 | -3.476162195 | HAL | -1.588015222 |
| EPB41L4A-AS1 | 1.764574685 | RPL6 | 2.249536454 |
| SNHG6 | 2.864593326 | RPL6 | 2.249536454 |
| TRIM52-AS1 | 2.337811404 | RPL6 | 2.249536454 |
| SNHG9 | 2.582897194 | RPL6 | 2.249536454 |
| EPB41L4A-AS1 | 1.764574685 | ESF1 | 1.819285808 |
| SNHG6 | 2.864593326 | ESF1 | 1.819285808 |
| LA16c-380H5.5 | -3.446980855 | CMTM1 | -2.069469327 |
| RP4-742C19.13 | -6.346685252 | ANGPT2 | -3.002835786 |
| CTC-250I14.6 | -6.710662207 | HOOK2 | -3.004583115 |
| RP11-274B21.10 | -2.34955842 | MAST3 | -2.155884435 |
| CTB-152G17.6 | -1.969668991 | MAST3 | -2.155884435 |
| SNHG22 | 2.908531389 | NDUFB7 | 1.621035192 |
| RP11-568N6.1 | 1.793391075 | GADD45B | 1.934232949 |
| SNHG6 | 2.864593326 | TOMM22 | 1.521720463 |
| EPB41L4A-AS1 | 1.764574685 | PNN | 1.690908165 |
| SNHG6 | 2.864593326 | PNN | 1.690908165 |
| SNHG6 | 2.864593326 | PFDN4 | 1.883143355 |
| RP11-290F5.2 | -2.567649204 | RNF24 | -2.327553897 |
| LINC01359 | -2.371602714 | RNF24 | -2.327553897 |
| AC093495.4 | -2.061520107 | RNF24 | -2.327553897 |
| RP11-59D5__B.2 | -1.66686715 | RNF24 | -2.327553897 |
| LINC01513 | -3.711658406 | RNF24 | -2.327553897 |
| RP11-321E2.4 | -2.040412365 | RNF24 | -2.327553897 |
| RP11-414H23.3 | -2.418471674 | RNF24 | -2.327553897 |
| RP11-875O11.3 | -3.045134873 | RNF24 | -2.327553897 |
| RP11-68I3.11 | -2.095759421 | RNF24 | -2.327553897 |
| RP11-619I22.1 | -3.476162195 | RNF24 | -2.327553897 |
| LA16c-380H5.5 | -3.446980855 | RNF24 | -2.327553897 |
| RP11-25K21.6 | -2.749251115 | RNF24 | -2.327553897 |
| RP11-455F5.6 | -3.416511699 | RNF24 | -2.327553897 |
| LA16c-380H5.5 | -3.446980855 | ZNF516 | -1.588486394 |
| SNHG6 | 2.864593326 | HTATSF1 | 1.548896328 |
| TRIM52-AS1 | 2.337811404 | HTATSF1 | 1.548896328 |
| RP11-408H1.3 | 1.944314838 | IRG1 | 2.69011798 |
| RP11-439A17.10 | 1.60291371 | IRG1 | 2.69011798 |
| RP11-480C16.1 | 1.873803983 | IRG1 | 2.69011798 |
| DGCR11 | -1.972880324 | GDPD3 | -2.284783754 |
| RP5-827C21.4 | 2.18767431 | C19orf53 | 3.485299734 |
| SNHG6 | 2.864593326 | C19orf53 | 3.485299734 |
| RP11-598F7.3 | 1.812838335 | TNNT1 | 2.085745316 |
| CTA-29F11.1 | 3.969693116 | CDC37 | 1.666174517 |
| EPB41L4A-AS1 | 1.764574685 | RPL18A | 1.761202041 |
| SNHG6 | 2.864593326 | RPL18A | 1.761202041 |
| AC093495.4 | -2.061520107 | CNTNAP3 | -2.618716105 |
| RP11-290F5.2 | -2.567649204 | MEGF9 | -2.240591859 |
| AC093495.4 | -2.061520107 | MEGF9 | -2.240591859 |
| RP1-55C23.7 | -4.934589001 | MEGF9 | -2.240591859 |
| RP11-59D5__B.2 | -1.66686715 | MEGF9 | -2.240591859 |
| RP11-290F5.1 | -2.675904236 | MEGF9 | -2.240591859 |
| RP11-321E2.4 | -2.040412365 | MEGF9 | -2.240591859 |
| RP11-875O11.3 | -3.045134873 | MEGF9 | -2.240591859 |
| RP11-68I3.11 | -2.095759421 | MEGF9 | -2.240591859 |
| RP11-619I22.1 | -3.476162195 | MEGF9 | -2.240591859 |
| RP11-25K21.6 | -2.749251115 | MEGF9 | -2.240591859 |
| RP11-455F5.6 | -3.416511699 | MEGF9 | -2.240591859 |
| RP11-568N6.1 | 1.793391075 | TNFSF8 | 1.994395171 |
| RP5-827C21.4 | 2.18767431 | EDF1 | 2.424742488 |
| SNHG6 | 2.864593326 | EDF1 | 2.424742488 |
| TRIM52-AS1 | 2.337811404 | EDF1 | 2.424742488 |
| SNHG22 | 2.908531389 | EDF1 | 2.424742488 |
| EPB41L4A-AS1 | 1.764574685 | RPL19 | 2.497573792 |
| SNHG6 | 2.864593326 | RPL19 | 2.497573792 |
| RP5-1142A6.2 | -3.776570831 | ENO3 | -1.610154914 |
| BIRC6-AS1 | -5.288097288 | ENO3 | -1.610154914 |
| AC004906.3 | -4.944081627 | ENO3 | -1.610154914 |
| AC009133.12 | -1.723329048 | ENO3 | -1.610154914 |
| SMARCA5-AS1 | -2.541712513 | ENO3 | -1.610154914 |
| AC005329.7 | -2.144322006 | ENO3 | -1.610154914 |
| RP11-849H4.4 | -4.799792287 | ENO3 | -1.610154914 |
| AP006621.8 | -6.292200789 | ENO3 | -1.610154914 |
| RP11-983P16.4 | -2.44829018 | ENO3 | -1.610154914 |
| RP11-317G6.1 | -5.892834112 | ENO3 | -1.610154914 |
| AC009133.15 | -4.621261441 | ENO3 | -1.610154914 |
| AC027601.1 | -3.205018383 | ENO3 | -1.610154914 |
| LA16c-358B7.3 | -6.399909599 | ENO3 | -1.610154914 |
| LA16c-390E6.5 | -5.626883218 | ENO3 | -1.610154914 |
| RP11-498C9.3 | -5.3947311 | ENO3 | -1.610154914 |
| AC010761.8 | -6.333382131 | ENO3 | -1.610154914 |
| RP11-452I5.2 | -4.527015005 | ENO3 | -1.610154914 |
| CTB-31O20.4 | -5.912842662 | ENO3 | -1.610154914 |
| AC005944.2 | -7.516561983 | ENO3 | -1.610154914 |
| CTD-3193O13.13 | -4.852551595 | ENO3 | -1.610154914 |
| LLNLR-284B4.1 | -6.420632191 | ENO3 | -1.610154914 |
| CTB-58E17.9 | -5.809887496 | ENO3 | -1.610154914 |
| LLNLR-307A6.1 | -4.622395882 | ENO3 | -1.610154914 |
| CTBP1-AS | -6.439383626 | ENO3 | -1.610154914 |
| AC133644.2 | 1.840685259 | CCL7 | 7.217188802 |
| RP11-408H1.3 | 1.944314838 | CCL8 | 4.337578901 |
| RP11-249C24.10 | 2.991734053 | CCL8 | 4.337578901 |
| RP11-480C16.1 | 1.873803983 | CCL8 | 4.337578901 |
| RP11-334J6.7 | -3.600624293 | SH3D19 | -5.109432042 |
| RP5-1142A6.2 | -3.776570831 | SH3D19 | -5.109432042 |
| BIRC6-AS1 | -5.288097288 | SH3D19 | -5.109432042 |
| SEMA3F-AS1 | -6.327723453 | SH3D19 | -5.109432042 |
| AC009133.12 | -1.723329048 | SH3D19 | -5.109432042 |
| KB-1208A12.3 | -6.747493135 | SH3D19 | -5.109432042 |
| RP11-849H4.4 | -4.799792287 | SH3D19 | -5.109432042 |
| AP006621.8 | -6.292200789 | SH3D19 | -5.109432042 |
| RP11-983P16.4 | -2.44829018 | SH3D19 | -5.109432042 |
| LA16c-358B7.3 | -6.399909599 | SH3D19 | -5.109432042 |
| AC010761.8 | -6.333382131 | SH3D19 | -5.109432042 |
| AC005944.2 | -7.516561983 | SH3D19 | -5.109432042 |
| RP11-290D2.6 | -6.532102297 | SH3D19 | -5.109432042 |
| RP4-742C19.13 | -6.346685252 | SH3D19 | -5.109432042 |
| XX-FW83563B9.5 | -2.486769511 | SH3D19 | -5.109432042 |
| CTBP1-AS | -6.439383626 | SH3D19 | -5.109432042 |
| CTA-29F11.1 | 3.969693116 | ANAPC15 | 1.626545118 |
| AC083884.8 | -4.699792638 | UPK2 | -2.294801967 |
| RP11-455F5.3 | -1.653267555 | MANSC1 | -2.487293905 |
| RP11-44F14.2 | -2.253372035 | MANSC1 | -2.487293905 |
| RP11-25K21.6 | -2.749251115 | MANSC1 | -2.487293905 |
| SNHG22 | 2.908531389 | COX6A1 | 2.038944816 |
| LINC01270 | -2.029860137 | SLC26A8 | -2.462078252 |
| RP11-321E2.4 | -2.040412365 | SLC26A8 | -2.462078252 |
| RP11-63N8.3 | -3.294590971 | TREML2 | -1.899968642 |
| EPB41L4A-AS1 | 1.764574685 | RPS12 | 1.628413985 |
| SNHG6 | 2.864593326 | IK | 1.672149458 |
| AC093495.4 | -2.061520107 | BTNL8 | -2.178401643 |
| RP11-59D5__B.2 | -1.66686715 | BTNL8 | -2.178401643 |
| RP11-321E2.4 | -2.040412365 | BTNL8 | -2.178401643 |
| RP11-455F5.3 | -1.653267555 | BTNL8 | -2.178401643 |
| EPB41L4A-AS1 | 1.764574685 | BRIX1 | 1.508439219 |
| RP11-290F5.2 | -2.567649204 | PFKFB4 | -2.255331663 |
| LINC01359 | -2.371602714 | PFKFB4 | -2.255331663 |
| RP11-59D5__B.2 | -1.66686715 | PFKFB4 | -2.255331663 |
| RP11-290F5.1 | -2.675904236 | PFKFB4 | -2.255331663 |
| RP11-875O11.3 | -3.045134873 | PFKFB4 | -2.255331663 |
| RP11-529H2.2 | -3.977727445 | PFKFB4 | -2.255331663 |
| RP11-68I3.11 | -2.095759421 | PFKFB4 | -2.255331663 |
| RP11-619I22.1 | -3.476162195 | PFKFB4 | -2.255331663 |
| LA16c-380H5.5 | -3.446980855 | PFKFB4 | -2.255331663 |
| RP11-25K21.6 | -2.749251115 | PFKFB4 | -2.255331663 |
| RP11-455F5.6 | -3.416511699 | PFKFB4 | -2.255331663 |
| EPB41L4A-AS1 | 1.764574685 | RPL24 | 2.141968739 |
| SNHG6 | 2.864593326 | RPL24 | 2.141968739 |
| TRIM52-AS1 | 2.337811404 | RPL24 | 2.141968739 |
| SNHG9 | 2.582897194 | RPL24 | 2.141968739 |
| EPB41L4A-AS1 | 1.764574685 | IFT57 | 1.903209925 |
| SNHG6 | 2.864593326 | IFT57 | 1.903209925 |
| RP11-25K21.6 | -2.749251115 | ABTB1 | -1.904679966 |
| RP11-482G13.1 | -2.287077989 | ABTB1 | -1.904679966 |
| LINC01001 | -2.273765643 | KIAA1257 | -2.588828249 |
| EPB41L4A-AS1 | 1.764574685 | EEF1B2 | 1.563774445 |
| SNHG6 | 2.864593326 | NCL | 1.549052774 |
| TRIM52-AS1 | 2.337811404 | NCL | 1.549052774 |
| EPB41L4A-AS1 | 1.764574685 | RPS15 | 2.232257145 |
| SNHG6 | 2.864593326 | RPS15 | 2.232257145 |
| TRIM52-AS1 | 2.337811404 | RPS15 | 2.232257145 |
| RP5-968J1.1 | -1.609094214 | GCA | -1.65958031 |
| RP11-875O11.3 | -3.045134873 | GCA | -1.65958031 |
| RP11-25K21.6 | -2.749251115 | GCA | -1.65958031 |
| LINC01002 | -1.967031255 | GCA | -1.65958031 |
| LINC01151 | 4.088686164 | ODC1 | 2.228006613 |
| EPB41L4A-AS1 | 1.764574685 | RPL22 | 2.440581436 |
| SNHG6 | 2.864593326 | RPL22 | 2.440581436 |
| SNHG6 | 2.864593326 | GADD45A | 2.121492934 |
| AC093495.4 | -2.061520107 | WLS | -2.214228214 |
| RP11-321E2.4 | -2.040412365 | WLS | -2.214228214 |
| CTD-2313J17.5 | -2.818185192 | WLS | -2.214228214 |
| RP5-827C21.4 | 2.18767431 | RPF1 | 2.012340527 |
| SNHG6 | 2.864593326 | RPF1 | 2.012340527 |
| TRIM52-AS1 | 2.337811404 | RPF1 | 2.012340527 |
| SNHG22 | 2.908531389 | RPF1 | 2.012340527 |
| SNHG6 | 2.864593326 | SYF2 | 1.767090016 |
| SCARNA9 | 2.697257584 | SYF2 | 1.767090016 |
| EPB41L4A-AS1 | 1.764574685 | RPS25 | 1.925663926 |
| SNHG6 | 2.864593326 | RPS25 | 1.925663926 |
| RP5-827C21.4 | 2.18767431 | HMGN3 | 1.545429228 |
| SNHG6 | 2.864593326 | HMGN3 | 1.545429228 |
| TRIM52-AS1 | 2.337811404 | HMGN3 | 1.545429228 |
| RP11-290F5.2 | -2.567649204 | TRPM6 | -1.835918624 |
| LINC01359 | -2.371602714 | TRPM6 | -1.835918624 |
| LINC01513 | -3.711658406 | TRPM6 | -1.835918624 |
| RP11-321E2.4 | -2.040412365 | TRPM6 | -1.835918624 |
| RP11-414H23.3 | -2.418471674 | TRPM6 | -1.835918624 |
| RP11-875O11.3 | -3.045134873 | TRPM6 | -1.835918624 |
| RP11-68I3.11 | -2.095759421 | TRPM6 | -1.835918624 |
| RP11-619I22.1 | -3.476162195 | TRPM6 | -1.835918624 |
| AL133245.2 | -1.880465661 | YIPF4 | -1.555309856 |
| RP11-249C24.10 | 2.991734053 | CD274 | 1.613297545 |
| LINC01001 | -2.273765643 | ARAP3 | -2.241682196 |
| RP11-25K21.6 | -2.749251115 | ARAP3 | -2.241682196 |
| RP11-482G13.1 | -2.287077989 | ARAP3 | -2.241682196 |
| AC093495.4 | -2.061520107 | APAF1 | -1.740965647 |
| RP11-59D5__B.2 | -1.66686715 | APAF1 | -1.740965647 |
| RP11-529H2.2 | -3.977727445 | APAF1 | -1.740965647 |
| RP11-68I3.11 | -2.095759421 | APAF1 | -1.740965647 |
| RP11-619I22.1 | -3.476162195 | APAF1 | -1.740965647 |
| RP4-535B20.4 | -3.840159331 | APAF1 | -1.740965647 |
| DGCR11 | -1.972880324 | APAF1 | -1.740965647 |
| RP11-640L9.1 | -2.817921017 | APAF1 | -1.740965647 |
| DGCR12 | -2.230457715 | APAF1 | -1.740965647 |
| CTB-152G17.6 | -1.969668991 | PDLIM2 | -1.854003622 |
| TRIM52-AS1 | 2.337811404 | COIL | 1.510450415 |
| TRIM52-AS1 | 2.337811404 | POLR3GL | 2.11275477 |
| EPB41L4A-AS1 | 1.764574685 | RPL21 | 1.662435723 |
| SNHG6 | 2.864593326 | RPL21 | 1.662435723 |
| EPB41L4A-AS1 | 1.764574685 | RPL5 | 1.576278487 |
| SNHG6 | 2.864593326 | RPL5 | 1.576278487 |
| TRIM52-AS1 | 2.337811404 | C19orf43 | 1.7949308 |
| SNHG6 | 2.864593326 | PFDN5 | 1.635966856 |
| CTA-29F11.1 | 3.969693116 | PFDN5 | 1.635966856 |
| RP11-338C15.3 | 1.860532589 | BATF3 | 1.519207685 |
| SNHG22 | 2.908531389 | SNRPC | 1.656786645 |
| LINC01272 | -1.638723871 | MBOAT7 | -1.873336961 |
| ARHGAP26-IT1 | -1.589528202 | MBOAT7 | -1.873336961 |
| RP11-63N8.3 | -3.294590971 | MBOAT7 | -1.873336961 |
| LINC01002 | -1.967031255 | MBOAT7 | -1.873336961 |
| RP11-482G13.1 | -2.287077989 | MBOAT7 | -1.873336961 |
| EPB41L4A-AS1 | 1.764574685 | RPL23 | 2.313298085 |
| SNHG6 | 2.864593326 | RPL23 | 2.313298085 |
| EPB41L4A-AS1 | 1.764574685 | SNRPD2 | 1.801828068 |
| SNHG6 | 2.864593326 | SNRPD2 | 1.801828068 |
| TRIM52-AS1 | 2.337811404 | SNRPD2 | 1.801828068 |
| TRIM52-AS1 | 2.337811404 | SNRPB2 | 1.625221645 |
| SCARNA9 | 2.697257584 | COX6B1 | 2.340345819 |
| SNHG22 | 2.908531389 | COX6B1 | 2.340345819 |
| SNHG22 | 2.908531389 | PRDX5 | 2.06035739 |
| AC004906.3 | -4.944081627 | PRR12 | -1.508528228 |
| AC009133.12 | -1.723329048 | PRR12 | -1.508528228 |
| AC005329.7 | -2.144322006 | PRR12 | -1.508528228 |
| RP11-849H4.4 | -4.799792287 | PRR12 | -1.508528228 |
| AC009133.15 | -4.621261441 | PRR12 | -1.508528228 |
| LA16c-358B7.3 | -6.399909599 | PRR12 | -1.508528228 |
| AC005944.2 | -7.516561983 | PRR12 | -1.508528228 |
| CTD-3193O13.13 | -4.852551595 | PRR12 | -1.508528228 |
| AC005785.2 | -2.363404517 | PRR12 | -1.508528228 |
| LLNLR-284B4.1 | -6.420632191 | PRR12 | -1.508528228 |
| CTB-58E17.9 | -5.809887496 | PRR12 | -1.508528228 |
| CTBP1-AS | -6.439383626 | PRR12 | -1.508528228 |
| EPB41L4A-AS1 | 1.764574685 | SBDS | 1.774347151 |
| SNHG6 | 2.864593326 | SBDS | 1.774347151 |
| SNHG9 | 2.582897194 | SBDS | 1.774347151 |
| RP11-529H2.2 | -3.977727445 | PLEKHG3 | -1.893446167 |
| RP11-68I3.11 | -2.095759421 | PLEKHG3 | -1.893446167 |
| RP11-619I22.1 | -3.476162195 | PLEKHG3 | -1.893446167 |
| RP11-552F3.4 | -2.218861354 | KDM4B | -1.833872303 |
| CTB-152G17.6 | -1.969668991 | KDM4B | -1.833872303 |
| RP11-290F5.2 | -2.567649204 | HIP1 | -1.603291368 |
| RP11-321E2.4 | -2.040412365 | HIP1 | -1.603291368 |
| RP11-875O11.3 | -3.045134873 | HIP1 | -1.603291368 |
| RP11-619I22.1 | -3.476162195 | HIP1 | -1.603291368 |
| AC093495.4 | -2.061520107 | LRFN1 | -2.438501669 |
| CTA-29F11.1 | 3.969693116 | ATF4 | 1.524425495 |
| LINC01002 | -1.967031255 | ALDH1A2 | -2.183602345 |
| EPB41L4A-AS1 | 1.764574685 | NGDN | 1.62418012 |
| SNHG6 | 2.864593326 | NGDN | 1.62418012 |
| TRIM52-AS1 | 2.337811404 | NGDN | 1.62418012 |
| LINC01272 | -1.638723871 | SEC14L1 | -1.670664191 |
| ARHGAP26-IT1 | -1.589528202 | SEC14L1 | -1.670664191 |
| RP11-63N8.3 | -3.294590971 | SEC14L1 | -1.670664191 |
| LINC01002 | -1.967031255 | SEC14L1 | -1.670664191 |
| RP11-482G13.1 | -2.287077989 | SEC14L1 | -1.670664191 |
| DHRSX-IT1 | -3.652291839 | CBFA2T3 | -2.725324866 |
| EPB41L4A-AS1 | 1.764574685 | RPL36 | 3.360109034 |
| SNHG6 | 2.864593326 | RPL36 | 3.360109034 |
| LINC01001 | -2.273765643 | ADGRE3 | -2.178338875 |
| RP11-482G13.1 | -2.287077989 | ADGRE3 | -2.178338875 |
| EPB41L4A-AS1 | 1.764574685 | RPL27 | 3.904643032 |
| SNHG6 | 2.864593326 | RPL27 | 3.904643032 |
| TRIM52-AS1 | 2.337811404 | RPL27 | 3.904643032 |
| SNHG9 | 2.582897194 | RPL27 | 3.904643032 |
| RP11-44F14.8 | -2.84244213 | AOC3 | -2.489673628 |
| RP11-44F14.2 | -2.253372035 | AOC2 | -1.780427877 |
| RP11-44F14.8 | -2.84244213 | AOC2 | -1.780427877 |
| RP11-25K21.6 | -2.749251115 | AOC2 | -1.780427877 |
| RP11-568N6.1 | 1.793391075 | RIN2 | 1.986099949 |
| RP5-827C21.4 | 2.18767431 | CCDC59 | 1.787185268 |
| SNHG6 | 2.864593326 | CCDC59 | 1.787185268 |
| TRIM52-AS1 | 2.337811404 | CCDC59 | 1.787185268 |
| AC093495.4 | -2.061520107 | LYVE1 | -3.126391035 |
| LINC01506 | -1.846796587 | LYVE1 | -3.126391035 |
| RP1-60N8.1 | -1.902862738 | LYVE1 | -3.126391035 |
| RP11-321E2.4 | -2.040412365 | LYVE1 | -3.126391035 |
| RP11-455F5.3 | -1.653267555 | LYVE1 | -3.126391035 |
| CTD-2313J17.5 | -2.818185192 | LYVE1 | -3.126391035 |
| RP11-619I22.1 | -3.476162195 | LYVE1 | -3.126391035 |
| RP11-455F5.6 | -3.416511699 | LYVE1 | -3.126391035 |
| RP11-569A11.1 | -2.900280345 | C14orf159 | -1.6121601 |
| AC093495.4 | -2.061520107 | C14orf159 | -1.6121601 |
| RP11-59D5__B.2 | -1.66686715 | C14orf159 | -1.6121601 |
| RP11-875O11.3 | -3.045134873 | C14orf159 | -1.6121601 |
| CTD-2313J17.5 | -2.818185192 | C14orf159 | -1.6121601 |
| RP11-580I16.2 | -1.799061931 | C14orf159 | -1.6121601 |
| RP11-619I22.1 | -3.476162195 | C14orf159 | -1.6121601 |
| RP4-535B20.4 | -3.840159331 | C14orf159 | -1.6121601 |
| RP11-455F5.6 | -3.416511699 | C14orf159 | -1.6121601 |
| RP11-640L9.1 | -2.817921017 | C14orf159 | -1.6121601 |
| EPB41L4A-AS1 | 1.764574685 | RPS15A | 3.561277223 |
| SNHG6 | 2.864593326 | RPS15A | 3.561277223 |
| SNHG9 | 2.582897194 | RPS15A | 3.561277223 |
| ARHGAP26-IT1 | -1.589528202 | PHC2 | -1.657296307 |
| RP11-63N8.3 | -3.294590971 | PHC2 | -1.657296307 |
| LINC01002 | -1.967031255 | PHC2 | -1.657296307 |
| RP11-290F5.2 | -2.567649204 | CCNJL | -2.157036615 |
| RP11-482G13.1 | -2.287077989 | CCNJL | -2.157036615 |
| AC093495.4 | -2.061520107 | AVIL | -2.383971293 |
| RP1-55C23.7 | -4.934589001 | AVIL | -2.383971293 |
| RP11-59D5__B.2 | -1.66686715 | AVIL | -2.383971293 |
| RP11-875O11.3 | -3.045134873 | AVIL | -2.383971293 |
| RP11-619I22.1 | -3.476162195 | AVIL | -2.383971293 |
| RP11-455F5.6 | -3.416511699 | AVIL | -2.383971293 |
| EPB41L4A-AS1 | 1.764574685 | HNRNPA1 | 1.774223051 |
| SNHG6 | 2.864593326 | HNRNPA1 | 1.774223051 |
| TRIM52-AS1 | 2.337811404 | HNRNPA1 | 1.774223051 |
| EPB41L4A-AS1 | 1.764574685 | LTV1 | 1.809412949 |
| SNHG6 | 2.864593326 | LTV1 | 1.809412949 |
| TRIM52-AS1 | 2.337811404 | LTV1 | 1.809412949 |
| ARHGAP26-IT1 | -1.589528202 | DYSF | -1.729307017 |
| SNHG6 | 2.864593326 | ATP6V1G1 | 1.729553889 |
| TRIM52-AS1 | 2.337811404 | ATP6V1G1 | 1.729553889 |
| CTA-29F11.1 | 3.969693116 | ATP6V1G1 | 1.729553889 |
| EPB41L4A-AS1 | 1.764574685 | RPL35 | 2.563612276 |
| SNHG6 | 2.864593326 | RPL35 | 2.563612276 |
| EPB41L4A-AS1 | 1.764574685 | RPS6 | 2.261431069 |
| SNHG6 | 2.864593326 | RPS6 | 2.261431069 |
| AC091878.1 | -1.986751235 | KIAA0319 | -1.830070385 |
| LINC01513 | -3.711658406 | KIAA0319 | -1.830070385 |
| RP11-875O11.3 | -3.045134873 | KIAA0319 | -1.830070385 |
| RP11-960L18.1 | -1.664004286 | KIAA0319 | -1.830070385 |
| RP11-624L4.1 | 2.173612088 | THBS1 | 2.609973004 |
| CTD-2033D15.2 | 2.889459651 | THBS1 | 2.609973004 |
| RP11-568N6.1 | 1.793391075 | DUSP5 | 1.718793404 |
| ARHGAP26-IT1 | -1.589528202 | ENTPD1 | -1.545016494 |
| RP11-429G19.3 | -1.994219503 | ENTPD1 | -1.545016494 |
| EPB41L4A-AS1 | 1.764574685 | RPS24 | 3.856583943 |
| SNHG6 | 2.864593326 | RPS24 | 3.856583943 |
| TRIM52-AS1 | 2.337811404 | RPS24 | 3.856583943 |
| TRIM52-AS1 | 2.337811404 | SSB | 1.817230107 |
| LINC01010 | 2.165729044 | PHLDA1 | 2.031871992 |
| CTD-2020K17.3 | -4.744792149 | SETD1B | -1.742376689 |
| RP5-827C21.4 | 2.18767431 | SRP14 | 1.987554715 |
| SNHG6 | 2.864593326 | SRP14 | 1.987554715 |
| TRIM52-AS1 | 2.337811404 | SRP14 | 1.987554715 |
| EPB41L4A-AS1 | 1.764574685 | RPS2 | 1.967973238 |
| SNHG6 | 2.864593326 | RPS2 | 1.967973238 |
| RP1-60N8.1 | -1.902862738 | DPEP3 | -1.971900546 |
| RP11-482G13.1 | -2.287077989 | DPEP3 | -1.971900546 |
| RP11-482G13.1 | -2.287077989 | SLC16A3 | -2.024100601 |
| RP11-619I22.1 | -3.476162195 | NLRP12 | -2.529249634 |
| CTD-3022G6.1 | -3.295125539 | NLRP12 | -2.529249634 |
| LA16c-380H5.5 | -3.446980855 | NLRP12 | -2.529249634 |
| CD81-AS1 | -2.319590468 | FAM71E1 | -2.825627793 |
| EPB41L4A-AS1 | 1.764574685 | RPS11 | 3.458291234 |
| RP5-827C21.4 | 2.18767431 | RPS11 | 3.458291234 |
| SNHG6 | 2.864593326 | RPS11 | 3.458291234 |
| TRIM52-AS1 | 2.337811404 | RPS11 | 3.458291234 |
| SNHG9 | 2.582897194 | RPS11 | 3.458291234 |
| EPB41L4A-AS1 | 1.764574685 | RPL13A | 1.673183437 |
| SNHG6 | 2.864593326 | RPL13A | 1.673183437 |
| EPB41L4A-AS1 | 1.764574685 | RPL11 | 3.306859785 |
| SNHG6 | 2.864593326 | RPL11 | 3.306859785 |
| SNHG9 | 2.582897194 | RPL11 | 3.306859785 |
| SNHG6 | 2.864593326 | UFC1 | 2.032997454 |
| TRIM52-AS1 | 2.337811404 | UFC1 | 2.032997454 |
| EPB41L4A-AS1 | 1.764574685 | RPS27A | 2.155815876 |
| SNHG6 | 2.864593326 | RPS27A | 2.155815876 |
| EPB41L4A-AS1 | 1.764574685 | RPL32 | 2.144204797 |
| SNHG6 | 2.864593326 | RPL32 | 2.144204797 |
| CTA-293F17.1 | 2.399523702 | PLA1A | 1.707094284 |
| RP11-849H4.4 | -4.799792287 | SLC26A1 | -2.87996579 |
| RP11-290F5.2 | -2.567649204 | ROPN1L | -1.951871475 |
| LINC01359 | -2.371602714 | ROPN1L | -1.951871475 |
| AC093495.4 | -2.061520107 | ROPN1L | -1.951871475 |
| LINC01506 | -1.846796587 | ROPN1L | -1.951871475 |
| RP1-60N8.1 | -1.902862738 | ROPN1L | -1.951871475 |
| RP11-59D5__B.2 | -1.66686715 | ROPN1L | -1.951871475 |
| LINC01513 | -3.711658406 | ROPN1L | -1.951871475 |
| RP11-321E2.4 | -2.040412365 | ROPN1L | -1.951871475 |
| RP11-414H23.3 | -2.418471674 | ROPN1L | -1.951871475 |
| RP11-875O11.3 | -3.045134873 | ROPN1L | -1.951871475 |
| RP11-256L6.3 | -1.696848309 | ROPN1L | -1.951871475 |
| RP11-619I22.1 | -3.476162195 | ROPN1L | -1.951871475 |
| RP11-455F5.6 | -3.416511699 | ROPN1L | -1.951871475 |
| EPB41L4A-AS1 | 1.764574685 | RPL37 | 3.047511952 |
| SNHG6 | 2.864593326 | RPL37 | 3.047511952 |
| TRIM52-AS1 | 2.337811404 | RPL37 | 3.047511952 |
| SNHG6 | 2.864593326 | BTF3 | 1.662805224 |
| RP11-321E2.4 | -2.040412365 | CREB5 | -2.529888891 |
| RP11-875O11.3 | -3.045134873 | CREB5 | -2.529888891 |
| RP11-619I22.1 | -3.476162195 | CREB5 | -2.529888891 |
| DGCR11 | -1.972880324 | SLC12A9 | -1.591024181 |
| EPB41L4A-AS1 | 1.764574685 | RPL7 | 1.656902139 |
| SNHG6 | 2.864593326 | RPL7 | 1.656902139 |
| RP5-827C21.4 | 2.18767431 | POLR2K | 1.693117155 |
| TRIM52-AS1 | 2.337811404 | POLR2K | 1.693117155 |
| SNHG6 | 2.864593326 | NDUFB9 | 2.033132041 |
| TRIM52-AS1 | 2.337811404 | NDUFB9 | 2.033132041 |
| EPB41L4A-AS1 | 1.764574685 | RPL7A | 1.570784553 |
| SNHG6 | 2.864593326 | RPL7A | 1.570784553 |
| RP11-274B21.10 | -2.34955842 | NOTCH1 | -2.067023654 |
| RP11-482G13.1 | -2.287077989 | NOTCH1 | -2.067023654 |
| SNHG6 | 2.864593326 | FAU | 2.08203673 |
| SCARNA9 | 2.697257584 | FAU | 2.08203673 |
| SNHG9 | 2.582897194 | FAU | 2.08203673 |
| CTA-29F11.1 | 3.969693116 | FAU | 2.08203673 |
| RP5-827C21.4 | 2.18767431 | CWC15 | 1.662249136 |
| SNHG6 | 2.864593326 | CWC15 | 1.662249136 |
| TRIM52-AS1 | 2.337811404 | CWC15 | 1.662249136 |
| LINC01272 | -1.638723871 | GLT1D1 | -1.721840145 |
| ARHGAP26-IT1 | -1.589528202 | GLT1D1 | -1.721840145 |
| RP5-968J1.1 | -1.609094214 | GLT1D1 | -1.721840145 |
| RP11-63N8.3 | -3.294590971 | GLT1D1 | -1.721840145 |
| LINC01002 | -1.967031255 | GLT1D1 | -1.721840145 |
| RP11-482G13.1 | -2.287077989 | GLT1D1 | -1.721840145 |
| RP11-529H2.2 | -3.977727445 | ARL11 | -2.374919762 |
| LA16c-380H5.5 | -3.446980855 | ARL11 | -2.374919762 |
| DGCR11 | -1.972880324 | ARL11 | -2.374919762 |
| MYCBP2-AS1 | -3.749444004 | FARP1 | -3.995620063 |
| LINC01272 | -1.638723871 | EPHB1 | -1.90122437 |
| LINC01506 | -1.846796587 | EPHB1 | -1.90122437 |
| LINC01002 | -1.967031255 | EPHB1 | -1.90122437 |
| SNHG6 | 2.864593326 | UQCRB | 2.481891679 |
| TRIM52-AS1 | 2.337811404 | UQCRB | 2.481891679 |
| EPB41L4A-AS1 | 1.764574685 | RPL30 | 2.318789554 |
| SNHG6 | 2.864593326 | RPL30 | 2.318789554 |
| SNHG9 | 2.582897194 | RPL30 | 2.318789554 |
| EPB41L4A-AS1 | 1.764574685 | EEF1A1 | 1.633649725 |
| SNHG6 | 2.864593326 | EEF1A1 | 1.633649725 |
| ARHGAP26-IT1 | -1.589528202 | KCNJ15 | -2.466932017 |
| RP11-63N8.3 | -3.294590971 | KCNJ15 | -2.466932017 |
| RP11-321E2.4 | -2.040412365 | KCNJ15 | -2.466932017 |
| LINC01002 | -1.967031255 | KCNJ15 | -2.466932017 |
| EPB41L4A-AS1 | 1.764574685 | RPL8 | 2.12062283 |
| SNHG6 | 2.864593326 | RPL8 | 2.12062283 |
| TRIM52-AS1 | 2.337811404 | RPL8 | 2.12062283 |
| RP11-44F14.2 | -2.253372035 | FBXL13 | -1.51311266 |
| SMARCA5-AS1 | -2.541712513 | GRIN2C | -4.841426817 |
| AC005944.2 | -7.516561983 | GRIN2C | -4.841426817 |
| RP11-290F5.2 | -2.567649204 | ACOX1 | -1.535420448 |
| RP1-55C23.7 | -4.934589001 | ACOX1 | -1.535420448 |
| RP11-59D5__B.2 | -1.66686715 | ACOX1 | -1.535420448 |
| RP11-875O11.3 | -3.045134873 | ACOX1 | -1.535420448 |
| RP11-529H2.2 | -3.977727445 | ACOX1 | -1.535420448 |
| RP11-68I3.11 | -2.095759421 | ACOX1 | -1.535420448 |
| RP11-619I22.1 | -3.476162195 | ACOX1 | -1.535420448 |
| RP11-25K21.6 | -2.749251115 | ACOX1 | -1.535420448 |
| RP11-455F5.6 | -3.416511699 | ACOX1 | -1.535420448 |
| LINC01272 | -1.638723871 | ALPL | -1.858447387 |
| LINC01506 | -1.846796587 | FCGR3B | -2.413446871 |
| RP11-249C24.10 | 2.991734053 | FAM71A | 1.965073403 |
| LINC01270 | -2.029860137 | RNF149 | -1.629984878 |
| ARHGAP26-IT1 | -1.589528202 | RNF149 | -1.629984878 |
| RP11-63N8.3 | -3.294590971 | RNF149 | -1.629984878 |
| AC093495.4 | -2.061520107 | TGFA | -2.454878499 |
| RP11-59D5__B.2 | -1.66686715 | TGFA | -2.454878499 |
| RP11-321E2.4 | -2.040412365 | TGFA | -2.454878499 |
| RP11-875O11.3 | -3.045134873 | TGFA | -2.454878499 |
| RP11-619I22.1 | -3.476162195 | TGFA | -2.454878499 |
| LA16c-380H5.5 | -3.446980855 | TGFA | -2.454878499 |
| RP11-455F5.6 | -3.416511699 | TGFA | -2.454878499 |
| AC093495.4 | -2.061520107 | CXCR1 | -3.276812524 |
| RP11-59D5__B.2 | -1.66686715 | CXCR1 | -3.276812524 |
| RP11-321E2.4 | -2.040412365 | CXCR1 | -3.276812524 |
| RP11-875O11.3 | -3.045134873 | CXCR1 | -3.276812524 |
| RP11-619I22.1 | -3.476162195 | CXCR1 | -3.276812524 |
| LINC01272 | -1.638723871 | WDFY3 | -1.695564755 |
| ARHGAP26-IT1 | -1.589528202 | WDFY3 | -1.695564755 |
| RP11-63N8.3 | -3.294590971 | WDFY3 | -1.695564755 |
| LINC01002 | -1.967031255 | WDFY3 | -1.695564755 |
| HRAT92 | 3.31677198 | PF4 | 3.341387223 |
| EPB41L4A-AS1 | 1.764574685 | AIMP1 | 1.516569705 |
| SNHG6 | 2.864593326 | AIMP1 | 1.516569705 |
| SNHG22 | 2.908531389 | HMGB2 | 2.109247245 |
| RP11-21C4.1 | 2.123169958 | RANBP3L | 1.848881536 |
| RP11-569A11.1 | -2.900280345 | F2RL1 | -2.604081434 |
| LINC01359 | -2.371602714 | F2RL1 | -2.604081434 |
| RP11-59D5__B.2 | -1.66686715 | F2RL1 | -2.604081434 |
| RP11-290F5.1 | -2.675904236 | F2RL1 | -2.604081434 |
| RP11-875O11.3 | -3.045134873 | F2RL1 | -2.604081434 |
| RP11-44F14.2 | -2.253372035 | F2RL1 | -2.604081434 |
| RP11-619I22.1 | -3.476162195 | F2RL1 | -2.604081434 |
| LA16c-380H5.5 | -3.446980855 | F2RL1 | -2.604081434 |
| RP4-535B20.4 | -3.840159331 | F2RL1 | -2.604081434 |
| RP11-25K21.6 | -2.749251115 | F2RL1 | -2.604081434 |
| RP11-455F5.6 | -3.416511699 | F2RL1 | -2.604081434 |
| EPB41L4A-AS1 | 1.764574685 | NSA2 | 2.27973958 |
| SNHG6 | 2.864593326 | NSA2 | 2.27973958 |
| TRIM52-AS1 | 2.337811404 | NSA2 | 2.27973958 |
| EPB41L4A-AS1 | 1.764574685 | RPS14 | 1.561013231 |
| SNHG6 | 2.864593326 | RPS14 | 1.561013231 |
| EPB41L4A-AS1 | 1.764574685 | RP9 | 1.882642296 |
| TRIM52-AS1 | 2.337811404 | RP9 | 1.882642296 |
| SNHG6 | 2.864593326 | RPL36AL | 3.513420795 |
| SCARNA9 | 2.697257584 | RPL36AL | 3.513420795 |
| SNHG22 | 2.908531389 | RPL36AL | 3.513420795 |
| RP11-408H1.3 | 1.944314838 | ZNF503 | 1.623283939 |
| RP11-249C24.10 | 2.991734053 | ZNF503 | 1.623283939 |
| TRIM52-AS1 | 2.337811404 | PSMC3 | 1.729700253 |
| SNHG22 | 2.908531389 | PSMC3 | 1.729700253 |
| EPB41L4A-AS1 | 1.764574685 | RPL27A | 2.407714303 |
| SNHG6 | 2.864593326 | RPL27A | 2.407714303 |
| TRIM52-AS1 | 2.337811404 | RPL27A | 2.407714303 |
| LINC01272 | -1.638723871 | STX3 | -1.504193814 |
| ARHGAP26-IT1 | -1.589528202 | STX3 | -1.504193814 |
| RP5-968J1.1 | -1.609094214 | STX3 | -1.504193814 |
| LINC01002 | -1.967031255 | STX3 | -1.504193814 |
| RP11-568N6.1 | 1.793391075 | C15orf48 | 3.133082856 |
| SNHG6 | 2.864593326 | ATP5L | 2.424687376 |
| TRIM52-AS1 | 2.337811404 | ATP5L | 2.424687376 |
| CTD-2013N24.2 | -3.112018693 | JSRP1 | -4.215074323 |
| PXN-AS1 | -2.276520444 | JSRP1 | -4.215074323 |
| RP5-1142A6.5 | -5.121273913 | JSRP1 | -4.215074323 |
| CTD-2231E14.8 | -5.249237907 | JSRP1 | -4.215074323 |
| RP11-290D2.6 | -6.532102297 | JSRP1 | -4.215074323 |
| LINC01359 | -2.371602714 | ATG16L2 | -1.885895874 |
| AC093495.4 | -2.061520107 | ATG16L2 | -1.885895874 |
| LINC01001 | -2.273765643 | ATG16L2 | -1.885895874 |
| RP11-59D5__B.2 | -1.66686715 | ATG16L2 | -1.885895874 |
| LINC01513 | -3.711658406 | ATG16L2 | -1.885895874 |
| RP11-875O11.3 | -3.045134873 | ATG16L2 | -1.885895874 |
| RP11-68I3.11 | -2.095759421 | ATG16L2 | -1.885895874 |
| RP11-619I22.1 | -3.476162195 | ATG16L2 | -1.885895874 |
| RP11-25K21.6 | -2.749251115 | ATG16L2 | -1.885895874 |
| RP11-455F5.6 | -3.416511699 | ATG16L2 | -1.885895874 |
| RP11-290F5.2 | -2.567649204 | TRANK1 | -1.606329737 |
| RP11-619I22.1 | -3.476162195 | TRANK1 | -1.606329737 |
| RP11-640L9.1 | -2.817921017 | TRANK1 | -1.606329737 |
| RP5-827C21.4 | 2.18767431 | HIST1H1E | 3.310788928 |
| TRIM52-AS1 | 2.337811404 | HIST1H1E | 3.310788928 |
| SNHG22 | 2.908531389 | HIST1H1E | 3.310788928 |
| WDR11-AS1 | 2.176752521 | SDPR | 2.408607763 |
| ITGA9-AS1 | 2.312932706 | SDPR | 2.408607763 |
| AP001189.4 | 2.800967044 | SDPR | 2.408607763 |
| SNHG6 | 2.864593326 | NDUFS5 | 3.515804528 |
| SNHG22 | 2.908531389 | ATP5I | 2.210175503 |
| DGCR11 | -1.972880324 | RGS14 | -1.616508996 |
| RP11-139H15.7 | -2.056467655 | RGS14 | -1.616508996 |
| RP11-408H1.3 | 1.944314838 | CXCL10 | 2.940882881 |
| RP11-439A17.10 | 1.60291371 | CXCL10 | 2.940882881 |
| RP11-249C24.10 | 2.991734053 | CXCL10 | 2.940882881 |
| RP11-480C16.1 | 1.873803983 | CXCL10 | 2.940882881 |
| RP11-408H1.3 | 1.944314838 | CXCL11 | 4.450548888 |
| RP11-439A17.10 | 1.60291371 | CXCL11 | 4.450548888 |
| RP11-249C24.10 | 2.991734053 | CXCL11 | 4.450548888 |
| RP11-480C16.1 | 1.873803983 | CXCL11 | 4.450548888 |
| RP5-827C21.4 | 2.18767431 | FAM103A1 | 1.54999478 |
| TRIM52-AS1 | 2.337811404 | FAM103A1 | 1.54999478 |
| SNHG22 | 2.908531389 | FAM103A1 | 1.54999478 |
| RP11-290F5.2 | -2.567649204 | TMEM154 | -1.600712925 |
| UBR5-AS1 | -1.690629634 | TMEM154 | -1.600712925 |
| RP11-321E2.4 | -2.040412365 | TMEM154 | -1.600712925 |
| RP11-875O11.3 | -3.045134873 | TMEM154 | -1.600712925 |
| RP11-256L6.3 | -1.696848309 | TMEM154 | -1.600712925 |
| RP11-619I22.1 | -3.476162195 | TMEM154 | -1.600712925 |
| RP11-25K21.6 | -2.749251115 | TMEM154 | -1.600712925 |
| TRIM52-AS1 | 2.337811404 | PA2G4 | 1.501224618 |
| RP1-60N8.1 | -1.902862738 | GPR27 | -1.646692296 |
| SNHG6 | 2.864593326 | TBCA | 1.843313545 |
| TRIM52-AS1 | 2.337811404 | TBCA | 1.843313545 |
| LINC01272 | -1.638723871 | RASGRP4 | -1.624480557 |
| LINC01001 | -2.273765643 | RASGRP4 | -1.624480557 |
| ARHGAP26-IT1 | -1.589528202 | RASGRP4 | -1.624480557 |
| RP5-968J1.1 | -1.609094214 | RASGRP4 | -1.624480557 |
| RP11-63N8.3 | -3.294590971 | RASGRP4 | -1.624480557 |
| LINC01002 | -1.967031255 | RASGRP4 | -1.624480557 |
| RP11-482G13.1 | -2.287077989 | RASGRP4 | -1.624480557 |
| RP11-849H4.4 | -4.799792287 | SLFNL1 | -2.32493306 |
| AC009133.15 | -4.621261441 | SLFNL1 | -2.32493306 |
| AC027601.1 | -3.205018383 | SLFNL1 | -2.32493306 |
| CTB-31O20.4 | -5.912842662 | SLFNL1 | -2.32493306 |
| AC005944.2 | -7.516561983 | SLFNL1 | -2.32493306 |
| LLNLR-284B4.1 | -6.420632191 | SLFNL1 | -2.32493306 |
| CTBP1-AS | -6.439383626 | SLFNL1 | -2.32493306 |
| LINC01513 | -3.711658406 | PDZD3 | -1.952142086 |
| C5orf66 | -6.333510852 | SNX32 | -5.319211527 |
| CTD-2020K17.3 | -4.744792149 | SNX32 | -5.319211527 |
| AC002401.1 | -4.787580451 | SNX32 | -5.319211527 |
| PXN-AS1 | -2.276520444 | SNX32 | -5.319211527 |
| RP5-940J5.9 | -6.018537982 | SNX32 | -5.319211527 |
| EPB41L4A-AS1 | 1.764574685 | RPL38 | 2.921543956 |
| SNHG6 | 2.864593326 | RPL38 | 2.921543956 |
| AC093495.4 | -2.061520107 | HSPA6 | -2.764800241 |
| RP11-59D5__B.2 | -1.66686715 | HSPA6 | -2.764800241 |
| LINC01513 | -3.711658406 | HSPA6 | -2.764800241 |
| RP11-321E2.4 | -2.040412365 | HSPA6 | -2.764800241 |
| RP11-875O11.3 | -3.045134873 | HSPA6 | -2.764800241 |
| RP11-619I22.1 | -3.476162195 | HSPA6 | -2.764800241 |
| LA16c-380H5.5 | -3.446980855 | HSPA6 | -2.764800241 |
| RP11-455F5.6 | -3.416511699 | HSPA6 | -2.764800241 |
| RP5-827C21.4 | 2.18767431 | TRMT112 | 2.047591444 |
| SNHG6 | 2.864593326 | TRMT112 | 2.047591444 |
| SCARNA9 | 2.697257584 | TRMT112 | 2.047591444 |
| SNHG22 | 2.908531389 | TRMT112 | 2.047591444 |
| AC091878.1 | -1.986751235 | MAB21L3 | -2.488463426 |
| RP11-290F5.2 | -2.567649204 | TNFRSF10C | -2.580503196 |
| LINC01359 | -2.371602714 | TNFRSF10C | -2.580503196 |
| RP3-393E18.2 | -2.365002687 | TNFRSF10C | -2.580503196 |
| RP11-290F5.1 | -2.675904236 | TNFRSF10C | -2.580503196 |
| RP11-321E2.4 | -2.040412365 | TNFRSF10C | -2.580503196 |
| RP11-414H23.3 | -2.418471674 | TNFRSF10C | -2.580503196 |
| RP11-256L6.3 | -1.696848309 | TNFRSF10C | -2.580503196 |
| RP11-619I22.1 | -3.476162195 | TNFRSF10C | -2.580503196 |
| RP11-556I13.2 | -2.610749876 | SULT1B1 | -1.843442763 |
| LINC01127 | -2.504251634 | SULT1B1 | -1.843442763 |
| RP11-482G13.1 | -2.287077989 | SLC19A1 | -2.928948437 |
| RP11-569A11.1 | -2.900280345 | TIGD3 | -2.041754201 |
| RP11-455F5.3 | -1.653267555 | TIGD3 | -2.041754201 |
| RP11-7F17.3 | -2.481000237 | PHOSPHO1 | -1.863343746 |
| AC093495.4 | -2.061520107 | CEP19 | -2.060902674 |
| RP11-59D5__B.2 | -1.66686715 | CEP19 | -2.060902674 |
| LINC01513 | -3.711658406 | CEP19 | -2.060902674 |
| RP11-321E2.4 | -2.040412365 | CEP19 | -2.060902674 |
| RP11-875O11.3 | -3.045134873 | CEP19 | -2.060902674 |
| CTD-2313J17.5 | -2.818185192 | CEP19 | -2.060902674 |
| RP11-619I22.1 | -3.476162195 | CEP19 | -2.060902674 |
| RP4-535B20.4 | -3.840159331 | CEP19 | -2.060902674 |
| RP11-455F5.6 | -3.416511699 | CEP19 | -2.060902674 |
| RP5-968J1.1 | -1.609094214 | TLR6 | -1.805656116 |
| RP11-290F5.1 | -2.675904236 | TLR6 | -1.805656116 |
| RP11-875O11.3 | -3.045134873 | TLR6 | -1.805656116 |
| RP11-44F14.2 | -2.253372035 | TLR6 | -1.805656116 |
| RP11-619I22.1 | -3.476162195 | TLR6 | -1.805656116 |
| LA16c-380H5.5 | -3.446980855 | TLR6 | -1.805656116 |
| RP11-25K21.6 | -2.749251115 | TLR6 | -1.805656116 |
| RP11-455F5.6 | -3.416511699 | TLR6 | -1.805656116 |
| EPB41L4A-AS1 | 1.764574685 | RPL4 | 1.755061602 |
| SNHG6 | 2.864593326 | RPL4 | 1.755061602 |
| RP11-290F5.2 | -2.567649204 | KY | -5.378964568 |
| RP1-60N8.1 | -1.902862738 | KY | -5.378964568 |
| RP11-290F5.1 | -2.675904236 | KY | -5.378964568 |
| RP11-619I22.1 | -3.476162195 | KY | -5.378964568 |
| RP11-455F5.6 | -3.416511699 | KY | -5.378964568 |
| RP11-875O11.3 | -3.045134873 | SLC22A1 | -1.960227788 |
| SNHG6 | 2.864593326 | LINC00116 | 3.056152831 |
| TRIM52-AS1 | 2.337811404 | LINC00116 | 3.056152831 |
| LINC00612 | -4.203395519 | A2M | -1.809132685 |
| RP11-290L1.2 | 2.306772709 | TUBB6 | 1.983076701 |
| SCARNA9 | 2.697257584 | COX8A | 2.859567919 |
| EPB41L4A-AS1 | 1.764574685 | RPLP2 | 1.876770258 |
| CTA-29F11.1 | 3.969693116 | SVBP | 1.945146789 |
| SNHG6 | 2.864593326 | RPS27 | 4.175167118 |
| TRIM52-AS1 | 2.337811404 | RPS27 | 4.175167118 |
| CTA-293F17.1 | 2.399523702 | MSC | 2.266821594 |
| LA16c-380H5.5 | -3.446980855 | FUT7 | -2.383976835 |
| RP11-290F5.2 | -2.567649204 | CXCR2 | -2.475802965 |
| AC093495.4 | -2.061520107 | CXCR2 | -2.475802965 |
| LINC01506 | -1.846796587 | CXCR2 | -2.475802965 |
| RP11-59D5__B.2 | -1.66686715 | CXCR2 | -2.475802965 |
| UBR5-AS1 | -1.690629634 | CXCR2 | -2.475802965 |
| RP11-321E2.4 | -2.040412365 | CXCR2 | -2.475802965 |
| RP11-455F5.3 | -1.653267555 | CXCR2 | -2.475802965 |
| RP11-875O11.3 | -3.045134873 | CXCR2 | -2.475802965 |
| RP11-256L6.3 | -1.696848309 | CXCR2 | -2.475802965 |
| RP11-619I22.1 | -3.476162195 | CXCR2 | -2.475802965 |
| RP11-290F5.2 | -2.567649204 | ST20 | -1.99202994 |
| RP11-290F5.1 | -2.675904236 | ST20 | -1.99202994 |
| RP11-455F5.3 | -1.653267555 | ST20 | -1.99202994 |
| RP11-44F14.2 | -2.253372035 | ST20 | -1.99202994 |
| RP11-619I22.1 | -3.476162195 | ST20 | -1.99202994 |
| RP11-25K21.6 | -2.749251115 | ST20 | -1.99202994 |
| EPB41L4A-AS1 | 1.764574685 | NPM1 | 1.751341257 |
| SNHG6 | 2.864593326 | NPM1 | 1.751341257 |
| LINC01359 | -2.371602714 | FRAT2 | -1.88062456 |
| AC093495.4 | -2.061520107 | FRAT2 | -1.88062456 |
| RP11-59D5__B.2 | -1.66686715 | FRAT2 | -1.88062456 |
| LINC01513 | -3.711658406 | FRAT2 | -1.88062456 |
| RP11-529H2.2 | -3.977727445 | FRAT2 | -1.88062456 |
| RP11-619I22.1 | -3.476162195 | FRAT2 | -1.88062456 |
| LA16c-380H5.5 | -3.446980855 | FRAT2 | -1.88062456 |
| RP11-290F5.2 | -2.567649204 | P2RY13 | -2.849032644 |
| LINC01359 | -2.371602714 | P2RY13 | -2.849032644 |
| RP11-59D5__B.2 | -1.66686715 | P2RY13 | -2.849032644 |
| LINC01513 | -3.711658406 | P2RY13 | -2.849032644 |
| RP11-290F5.1 | -2.675904236 | P2RY13 | -2.849032644 |
| RP11-875O11.3 | -3.045134873 | P2RY13 | -2.849032644 |
| RP11-529H2.2 | -3.977727445 | P2RY13 | -2.849032644 |
| RP11-68I3.11 | -2.095759421 | P2RY13 | -2.849032644 |
| RP11-619I22.1 | -3.476162195 | P2RY13 | -2.849032644 |
| LA16c-380H5.5 | -3.446980855 | P2RY13 | -2.849032644 |
| RP4-535B20.4 | -3.840159331 | P2RY13 | -2.849032644 |
| RP11-455F5.6 | -3.416511699 | P2RY13 | -2.849032644 |
| RP11-640L9.1 | -2.817921017 | P2RY13 | -2.849032644 |
| RP11-290F5.1 | -2.675904236 | OR52K2 | -3.097417005 |
| RP11-619I22.1 | -3.476162195 | OR52K2 | -3.097417005 |
| EPB41L4A-AS1 | 1.764574685 | SNRPE | 1.683089462 |
| RP5-968J1.1 | -1.609094214 | CHST15 | -1.62353917 |
| EPB41L4A-AS1 | 1.764574685 | RPS17 | 1.747974412 |
| SNHG6 | 2.864593326 | RPS17 | 1.747974412 |
| RP11-54A4.2 | -1.833630204 | ADGRG3 | -1.873012795 |
| CTD-2530H12.2 | -1.807187624 | ADGRG3 | -1.873012795 |
| EPB41L4A-AS1 | 1.764574685 | RPL35A | 2.3904641 |
| SNHG6 | 2.864593326 | RPL35A | 2.3904641 |
| TRIM52-AS1 | 2.337811404 | RPL35A | 2.3904641 |
| SNHG22 | 2.908531389 | UQCR10 | 1.594787816 |
| RP5-827C21.4 | 2.18767431 | HIST2H2AC | 2.072884694 |
| SNHG22 | 2.908531389 | HIST2H2AC | 2.072884694 |
| RP5-827C21.4 | 2.18767431 | HIST2H2AB | 2.244521845 |
| SNHG22 | 2.908531389 | HIST2H2AB | 2.244521845 |
| RP5-827C21.4 | 2.18767431 | NDUFA12 | 1.919123185 |
| SNHG22 | 2.908531389 | NDUFA12 | 1.919123185 |
| SRGAP2-AS1 | 2.465963975 | PRR16 | 2.51399095 |
| RP11-338C15.3 | 1.860532589 | PRR16 | 2.51399095 |
| RP11-568N6.1 | 1.793391075 | PRR16 | 2.51399095 |
| CTA-29F11.1 | 3.969693116 | NDUFA6 | 2.168892728 |
| EPB41L4A-AS1 | 1.764574685 | NSMCE3 | 2.094252098 |
| SNHG6 | 2.864593326 | NSMCE3 | 2.094252098 |
| TRIM52-AS1 | 2.337811404 | NSMCE3 | 2.094252098 |
| UBR5-AS1 | -1.690629634 | KIAA0825 | -1.520636294 |
| RP11-875O11.3 | -3.045134873 | KIAA0825 | -1.520636294 |
| RP11-25K21.6 | -2.749251115 | KIAA0825 | -1.520636294 |
| U73166.2 | -2.627557899 | C7orf61 | -2.124832155 |
| LINC01506 | -1.846796587 | CYP4F12 | -2.317395401 |
| RP11-455F5.3 | -1.653267555 | CYP4F12 | -2.317395401 |
| RP11-274B21.10 | -2.34955842 | RXRA | -1.873195856 |
| EPB41L4A-AS1 | 1.764574685 | RPS23 | 2.352127641 |
| SNHG6 | 2.864593326 | RPS23 | 2.352127641 |
| RP11-25K21.6 | -2.749251115 | CYP4F3 | -1.988939654 |
| RP5-1142A6.2 | -3.776570831 | ZACN | -4.274197168 |
| BIRC6-AS1 | -5.288097288 | ZACN | -4.274197168 |
| AC009133.12 | -1.723329048 | ZACN | -4.274197168 |
| RP11-849H4.4 | -4.799792287 | ZACN | -4.274197168 |
| AP006621.8 | -6.292200789 | ZACN | -4.274197168 |
| RP11-317G6.1 | -5.892834112 | ZACN | -4.274197168 |
| AC009133.15 | -4.621261441 | ZACN | -4.274197168 |
| AC027601.1 | -3.205018383 | ZACN | -4.274197168 |
| LA16c-358B7.3 | -6.399909599 | ZACN | -4.274197168 |
| LA16c-390E6.5 | -5.626883218 | ZACN | -4.274197168 |
| RP11-498C9.3 | -5.3947311 | ZACN | -4.274197168 |
| RP11-452I5.2 | -4.527015005 | ZACN | -4.274197168 |
| CTB-31O20.4 | -5.912842662 | ZACN | -4.274197168 |
| AC005944.2 | -7.516561983 | ZACN | -4.274197168 |
| CTD-3193O13.13 | -4.852551595 | ZACN | -4.274197168 |
| CTD-2231E14.8 | -5.249237907 | ZACN | -4.274197168 |
| RP11-290D2.6 | -6.532102297 | ZACN | -4.274197168 |
| LLNLR-284B4.1 | -6.420632191 | ZACN | -4.274197168 |
| CTB-58E17.9 | -5.809887496 | ZACN | -4.274197168 |
| RP11-12J10.4 | -2.235703045 | ZACN | -4.274197168 |
| LLNLR-307A6.1 | -4.622395882 | ZACN | -4.274197168 |
| CTBP1-AS | -6.439383626 | ZACN | -4.274197168 |
| SNHG6 | 2.864593326 | PTMA | 2.57579334 |
| TRIM52-AS1 | 2.337811404 | PTMA | 2.57579334 |
| AC093495.4 | -2.061520107 | RP11-723O4.6 | -2.391135753 |
| RP5-827C21.4 | 2.18767431 | HIST1H1C | 2.994628044 |
| TRIM52-AS1 | 2.337811404 | HIST1H1C | 2.994628044 |
| LINC01272 | -1.638723871 | C3orf62 | -1.54584218 |
| ARHGAP26-IT1 | -1.589528202 | C3orf62 | -1.54584218 |
| RP11-63N8.3 | -3.294590971 | C3orf62 | -1.54584218 |
| LINC01002 | -1.967031255 | C3orf62 | -1.54584218 |
| RP11-482G13.1 | -2.287077989 | C3orf62 | -1.54584218 |
| RP11-249C24.10 | 2.991734053 | FAM72B | 2.681385077 |
| EPB41L4A-AS1 | 1.764574685 | RPL14 | 2.282562425 |
| SNHG6 | 2.864593326 | RPL14 | 2.282562425 |
| RP11-290F5.2 | -2.567649204 | LRRK2 | -1.961838124 |
| RP11-290F5.1 | -2.675904236 | LRRK2 | -1.961838124 |
| RP11-321E2.4 | -2.040412365 | LRRK2 | -1.961838124 |
| RP11-414H23.3 | -2.418471674 | LRRK2 | -1.961838124 |
| RP11-875O11.3 | -3.045134873 | LRRK2 | -1.961838124 |
| RP11-256L6.3 | -1.696848309 | LRRK2 | -1.961838124 |
| RP11-619I22.1 | -3.476162195 | LRRK2 | -1.961838124 |
| RP11-25K21.6 | -2.749251115 | LRRK2 | -1.961838124 |
| RP11-455F5.6 | -3.416511699 | LRRK2 | -1.961838124 |
| EPB41L4A-AS1 | 1.764574685 | NACA | 1.99124171 |
| SNHG6 | 2.864593326 | NACA | 1.99124171 |
| RP5-968J1.1 | -1.609094214 | MME | -2.056113816 |
| UBR5-AS1 | -1.690629634 | MME | -2.056113816 |
| RP11-44F14.2 | -2.253372035 | MME | -2.056113816 |
| RP11-25K21.6 | -2.749251115 | MME | -2.056113816 |
| SRGAP2-AS1 | 2.465963975 | FAM72A | 2.678738319 |
| RP11-249C24.10 | 2.991734053 | FAM72A | 2.678738319 |
| LINC01272 | -1.638723871 | TECPR2 | -1.783839512 |
| ARHGAP26-IT1 | -1.589528202 | TECPR2 | -1.783839512 |
| RP5-968J1.1 | -1.609094214 | TECPR2 | -1.783839512 |
| RP11-63N8.3 | -3.294590971 | TECPR2 | -1.783839512 |
| LINC01002 | -1.967031255 | TECPR2 | -1.783839512 |
| RP11-482G13.1 | -2.287077989 | TECPR2 | -1.783839512 |
| RP5-827C21.4 | 2.18767431 | HIST1H4C | 2.335394942 |
| SNHG22 | 2.908531389 | HIST1H4C | 2.335394942 |
| SNHG6 | 2.864593326 | RPL37A | 3.064014698 |
| TRIM52-AS1 | 2.337811404 | RPL37A | 3.064014698 |
| RP11-290F5.2 | -2.567649204 | FAM212B | -2.530629219 |
| RP11-569A11.1 | -2.900280345 | FAM212B | -2.530629219 |
| LINC01359 | -2.371602714 | FAM212B | -2.530629219 |
| AC093495.4 | -2.061520107 | FAM212B | -2.530629219 |
| RP11-59D5__B.2 | -1.66686715 | FAM212B | -2.530629219 |
| RP3-393E18.2 | -2.365002687 | FAM212B | -2.530629219 |
| LINC01513 | -3.711658406 | FAM212B | -2.530629219 |
| RP11-290F5.1 | -2.675904236 | FAM212B | -2.530629219 |
| RP11-321E2.4 | -2.040412365 | FAM212B | -2.530629219 |
| RP11-414H23.3 | -2.418471674 | FAM212B | -2.530629219 |
| RP11-875O11.3 | -3.045134873 | FAM212B | -2.530629219 |
| RP11-529H2.2 | -3.977727445 | FAM212B | -2.530629219 |
| RP11-619I22.1 | -3.476162195 | FAM212B | -2.530629219 |
| LA16c-380H5.5 | -3.446980855 | FAM212B | -2.530629219 |
| RP4-535B20.4 | -3.840159331 | FAM212B | -2.530629219 |
| RP11-455F5.6 | -3.416511699 | FAM212B | -2.530629219 |
| SNHG22 | 2.908531389 | HIST1H2BK | 2.394908148 |
| EPB41L4A-AS1 | 1.764574685 | RPL12 | 3.002948965 |
| SNHG6 | 2.864593326 | RPL12 | 3.002948965 |
| WDR11-AS1 | 2.176752521 | DNM3 | 1.912159855 |
| AP001189.4 | 2.800967044 | DNM3 | 1.912159855 |
| RP11-568N6.1 | 1.793391075 | C1orf122 | 1.550788003 |
| EPB41L4A-AS1 | 1.764574685 | RPL23A | 3.126682703 |
| SNHG6 | 2.864593326 | RPL23A | 3.126682703 |
| TRIM52-AS1 | 2.337811404 | RPL23A | 3.126682703 |
| SNHG9 | 2.582897194 | RPL23A | 3.126682703 |
| RP11-290F5.2 | -2.567649204 | MSRB1 | -2.79246965 |
| AP001434.2 | -3.767592809 | MSRB1 | -2.79246965 |
| RP11-875O11.3 | -3.045134873 | MSRB1 | -2.79246965 |
| RP11-619I22.1 | -3.476162195 | MSRB1 | -2.79246965 |
| RP11-25K21.6 | -2.749251115 | MSRB1 | -2.79246965 |
| RP11-455F5.6 | -3.416511699 | MSRB1 | -2.79246965 |
| LINC01002 | -1.967031255 | MSRB1 | -2.79246965 |
| RP11-482G13.1 | -2.287077989 | MSRB1 | -2.79246965 |
| EPB41L4A-AS1 | 1.764574685 | RPL10A | 1.71082343 |
| SNHG6 | 2.864593326 | RPL10A | 1.71082343 |
| SNHG6 | 2.864593326 | MT-CO3 | 3.025011387 |
| CTA-29F11.1 | 3.969693116 | MT-CO3 | 3.025011387 |
| LINC00989 | 2.301431884 | MFAP3L | 1.892943419 |
| AC093495.4 | -2.061520107 | NHSL2 | -2.301888988 |
| RP11-59D5__B.2 | -1.66686715 | NHSL2 | -2.301888988 |
| CTD-2313J17.5 | -2.818185192 | NHSL2 | -2.301888988 |
| RP11-619I22.1 | -3.476162195 | NHSL2 | -2.301888988 |
| RP4-535B20.4 | -3.840159331 | NHSL2 | -2.301888988 |
| RP11-640L9.1 | -2.817921017 | NHSL2 | -2.301888988 |
| EPB41L4A-AS1 | 1.764574685 | GNB2L1 | 2.044983305 |
| SNHG6 | 2.864593326 | GNB2L1 | 2.044983305 |
| TRIM52-AS1 | 2.337811404 | GNB2L1 | 2.044983305 |
| SNHG9 | 2.582897194 | GNB2L1 | 2.044983305 |
| CTD-2020K17.3 | -4.744792149 | CCDC85C | -1.839179283 |
| RP11-182J23.1 | -2.008648376 | PLIN5 | -2.587526355 |
| FAM157C | -2.283461999 | PLIN5 | -2.587526355 |
| RP11-482G13.1 | -2.287077989 | PLIN5 | -2.587526355 |
| RP11-408H1.3 | 1.944314838 | FAM72D | 2.018101137 |
| RP11-249C24.10 | 2.991734053 | FAM72D | 2.018101137 |
| AP006621.8 | -6.292200789 | C1orf167 | -3.030317017 |
| LA16c-358B7.3 | -6.399909599 | C1orf167 | -3.030317017 |
| RP11-498C9.3 | -5.3947311 | C1orf167 | -3.030317017 |
| AP006621.8 | -6.292200789 | LINGO3 | -4.394989144 |
| RP5-827C21.4 | 2.18767431 | UBA52 | 2.589789736 |
| SNHG6 | 2.864593326 | UBA52 | 2.589789736 |
| TRIM52-AS1 | 2.337811404 | UBA52 | 2.589789736 |
| SCARNA9 | 2.697257584 | UBA52 | 2.589789736 |
| SNHG9 | 2.582897194 | UBA52 | 2.589789736 |
| SNHG6 | 2.864593326 | NOL7 | 1.725604236 |
| EPB41L4A-AS1 | 1.764574685 | RPL41 | 2.992470049 |
| SNHG6 | 2.864593326 | RPL41 | 2.992470049 |
| SNHG9 | 2.582897194 | RPL41 | 2.992470049 |
| EPB41L4A-AS1 | 1.764574685 | RPS18 | 2.498629523 |
| SNHG6 | 2.864593326 | RPS18 | 2.498629523 |
| RP5-827C21.4 | 2.18767431 | TMA7 | 1.650664343 |
| SNHG6 | 2.864593326 | TMA7 | 1.650664343 |
| TRIM52-AS1 | 2.337811404 | TMA7 | 1.650664343 |
| SNHG22 | 2.908531389 | TMA7 | 1.650664343 |
| RP5-827C21.4 | 2.18767431 | HIST1H2BN | 2.699926624 |
| SNHG22 | 2.908531389 | HIST1H2BN | 2.699926624 |
| LINC01272 | -1.638723871 | NFAM1 | -1.521131428 |
| LINC01001 | -2.273765643 | NFAM1 | -1.521131428 |
| ARHGAP26-IT1 | -1.589528202 | NFAM1 | -1.521131428 |
| RP5-968J1.1 | -1.609094214 | NFAM1 | -1.521131428 |
| RP11-63N8.3 | -3.294590971 | NFAM1 | -1.521131428 |
| LINC01002 | -1.967031255 | NFAM1 | -1.521131428 |
| RP11-482G13.1 | -2.287077989 | NFAM1 | -1.521131428 |
| RP5-827C21.4 | 2.18767431 | MIF | 3.811961209 |
| SNHG6 | 2.864593326 | MIF | 3.811961209 |
| TRIM52-AS1 | 2.337811404 | MIF | 3.811961209 |
| LINC01359 | -2.371602714 | PTTG2 | -1.836378546 |
| LINC01513 | -3.711658406 | PTTG2 | -1.836378546 |
| RP11-875O11.3 | -3.045134873 | PTTG2 | -1.836378546 |
| RP11-619I22.1 | -3.476162195 | PTTG2 | -1.836378546 |
| LA16c-380H5.5 | -3.446980855 | PTTG2 | -1.836378546 |
| RP5-968J1.1 | -1.609094214 | MGAM | -1.599540407 |
| RP11-529H2.2 | -3.977727445 | C6orf229 | -3.005162698 |
| RP11-640L9.1 | -2.817921017 | C6orf229 | -3.005162698 |
| DGCR12 | -2.230457715 | C6orf229 | -3.005162698 |
| AP001434.2 | -3.767592809 | CTB-50L17.14 | -3.03249441 |
| RP5-827C21.4 | 2.18767431 | HIST1H2AK | 2.820567186 |
| SNHG22 | 2.908531389 | HIST1H2AK | 2.820567186 |
| RP5-827C21.4 | 2.18767431 | HIST1H3A | 1.596181341 |
| RP5-827C21.4 | 2.18767431 | HIST1H4E | 3.857207982 |
| SNHG6 | 2.864593326 | HIST1H4E | 3.857207982 |
| TRIM52-AS1 | 2.337811404 | HIST1H4E | 3.857207982 |
| SNHG22 | 2.908531389 | HIST1H4E | 3.857207982 |
| RP5-827C21.4 | 2.18767431 | HIST1H2AE | 2.816061827 |
| SNHG22 | 2.908531389 | HIST1H2AE | 2.816061827 |
| SNHG22 | 2.908531389 | HIST1H2BF | 2.29192449 |
| RP4-742C19.13 | -6.346685252 | FP325331.1 | -3.928482078 |
| RP11-849H4.4 | -4.799792287 | AC009065.4 | -5.614967474 |
| RP11-867G23.4 | -5.16668441 | AC009065.4 | -5.614967474 |
| RP11-106M3.3 | -4.791958665 | AC009065.4 | -5.614967474 |
| CTD-2033A16.3 | -2.879467759 | AC009065.4 | -5.614967474 |
| RP11-498C9.3 | -5.3947311 | AC009065.4 | -5.614967474 |
| CTB-31O20.4 | -5.912842662 | AC009065.4 | -5.614967474 |
| AC005944.2 | -7.516561983 | AC009065.4 | -5.614967474 |
| CTBP1-AS | -6.439383626 | AC009065.4 | -5.614967474 |
| CTD-2020K17.3 | -4.744792149 | AP000769.1 | -10.85374807 |
| AC002401.1 | -4.787580451 | AP000769.1 | -10.85374807 |
| PXN-AS1 | -2.276520444 | AP000769.1 | -10.85374807 |
| RP1-56K13.3 | -6.900777664 | AP000769.1 | -10.85374807 |

## Table S5. The lncRNA targets in the LTPP/RP group

| **Lnc_Name** | **Lnc_log2FC(LTPP/RP)** | **Gene_Name** | **Gene_log2FC(LTPP/RP)** |
| --- | --- | --- | --- |
| LINC01272 | 2.970869212 | MMP25 | 3.729914109 |
| LINC01001 | 2.595275407 | MMP25 | 3.729914109 |
| RP5-968J1.1 | 3.407652425 | MMP25 | 3.729914109 |
| RP11-34P13.13 | 2.829181741 | MMP25 | 3.729914109 |
| RP11-875O11.3 | 4.013157441 | MMP25 | 3.729914109 |
| RP11-25K21.6 | 2.76934248 | MMP25 | 3.729914109 |
| RP11-81A1.6 | 1.759373228 | MMP25 | 3.729914109 |
| LINC01002 | 2.871347896 | MMP25 | 3.729914109 |
| RP11-482G13.1 | 2.681553651 | MMP25 | 3.729914109 |
| RP11-256L6.3 | 4.089504337 | ABHD5 | 1.839799606 |
| RP11-81A1.6 | 1.759373228 | ABHD5 | 1.839799606 |
| SPACA6 | 3.245939503 | SEMA3B | 3.026656502 |
| RP11-10J21.4 | 2.837409284 | SLC45A4 | 2.672404549 |
| RP11-81A1.6 | 1.759373228 | SLC45A4 | 2.672404549 |
| LINC01002 | 2.871347896 | SLC45A4 | 2.672404549 |
| RP11-482G13.1 | 2.681553651 | SLC45A4 | 2.672404549 |
| RP5-968J1.1 | 3.407652425 | DAPK2 | 2.509585751 |
| RP11-44F14.2 | 2.453885592 | DAPK2 | 2.509585751 |
| RP11-44F14.8 | 2.459485146 | DAPK2 | 2.509585751 |
| RP11-619I22.1 | 2.508888219 | DAPK2 | 2.509585751 |
| RP11-25K21.6 | 2.76934248 | DAPK2 | 2.509585751 |
| RP11-81A1.6 | 1.759373228 | DAPK2 | 2.509585751 |
| KB-1208A12.3 | -1.632756276 | NRIP2 | -1.716656492 |
| AC005944.2 | -1.571335159 | NRIP2 | -1.716656492 |
| LLNLR-284B4.1 | -1.693631408 | NRIP2 | -1.716656492 |
| RP11-153M7.5 | 4.52870836 | MXD1 | 3.090733569 |
| SPACA6 | 3.245939503 | DGAT2 | 4.035192887 |
| CTD-2530H12.2 | 3.721723258 | DGAT2 | 4.035192887 |
| RP11-290F5.2 | 2.728772492 | FAR2 | 1.500345779 |
| RP11-321E2.4 | 5.833782645 | FAR2 | 1.500345779 |
| RP11-619I22.1 | 2.508888219 | FAR2 | 1.500345779 |
| SPACA6 | 3.245939503 | SBNO2 | 1.505270211 |
| NEAT1 | 2.088068811 | SBNO2 | 1.505270211 |
| SPACA6 | 3.245939503 | BCL3 | 1.570294437 |
| LINC01002 | 2.871347896 | ST6GALNAC2 | 2.738163501 |
| RP11-482G13.1 | 2.681553651 | ST6GALNAC2 | 2.738163501 |
| RP11-290F5.2 | 2.728772492 | MRVI1 | 3.091470489 |
| LINC01359 | 2.490379801 | MRVI1 | 3.091470489 |
| RP11-321E2.4 | 5.833782645 | MRVI1 | 3.091470489 |
| RP11-414H23.3 | 4.982428435 | MRVI1 | 3.091470489 |
| RP11-619I22.1 | 2.508888219 | MRVI1 | 3.091470489 |
| LINC01272 | 2.970869212 | ALPK1 | 2.680410172 |
| RP11-380G5.2 | 3.24768833 | ALPK1 | 2.680410172 |
| LINC01506 | 4.30358908 | ALPK1 | 2.680410172 |
| LINC01272 | 2.970869212 | FRY | 1.501336272 |
| RP5-968J1.1 | 3.407652425 | FRY | 1.501336272 |
| RP11-875O11.3 | 4.013157441 | FRY | 1.501336272 |
| RP11-44F14.2 | 2.453885592 | FRY | 1.501336272 |
| RP11-25K21.6 | 2.76934248 | FRY | 1.501336272 |
| RP11-81A1.6 | 1.759373228 | FRY | 1.501336272 |
| RP4-647C14.3 | 2.087851208 | PICALM | 1.502143007 |
| RP11-321E2.4 | 5.833782645 | HAL | 2.417203894 |
| RP11-875O11.3 | 4.013157441 | HAL | 2.417203894 |
| RP11-256L6.3 | 4.089504337 | HAL | 2.417203894 |
| RP11-619I22.1 | 2.508888219 | HAL | 2.417203894 |
| RP11-81A1.6 | 1.759373228 | HAL | 2.417203894 |
| RP11-342M3.5 | 2.052316418 | CD82 | 1.962957865 |
| CTB-41I6.1 | 2.338928111 | PPP1R15A | 2.250932207 |
| LINC01272 | 2.970869212 | PGS1 | 1.555178145 |
| LINC01001 | 2.595275407 | PGS1 | 1.555178145 |
| ARHGAP26-IT1 | 2.137446203 | PGS1 | 1.555178145 |
| RP11-482G13.1 | 2.681553651 | PGS1 | 1.555178145 |
| RP11-153M7.5 | 4.52870836 | SRPK1 | 1.659373538 |
| RP11-568N6.1 | 1.999368955 | GADD45B | 1.527218701 |
| AP000355.2 | 3.35116668 | UPB1 | 3.090504975 |
| NEAT1 | 2.088068811 | CYTH4 | 1.797514981 |
| SPACA6 | 3.245939503 | PACSIN2 | 1.61392979 |
| LINC01272 | 2.970869212 | NCF4 | 2.527390661 |
| RP5-968J1.1 | 3.407652425 | NCF4 | 2.527390661 |
| RP11-256L6.3 | 4.089504337 | NCF4 | 2.527390661 |
| RP11-81A1.6 | 1.759373228 | NCF4 | 2.527390661 |
| LINC01002 | 2.871347896 | NCF4 | 2.527390661 |
| PCBP1-AS1 | 2.035458499 | CSF2RB | 1.897924774 |
| RP11-63N8.3 | 7.503029032 | CSF2RB | 1.897924774 |
| RP11-81A1.6 | 1.759373228 | CSF2RB | 1.897924774 |
| LINC01002 | 2.871347896 | CSF2RB | 1.897924774 |
| RP11-482G13.1 | 2.681553651 | CSF2RB | 1.897924774 |
| RP11-380G5.2 | 3.24768833 | ITPK1 | 1.624102942 |
| RP11-63N8.3 | 7.503029032 | ITPK1 | 1.624102942 |
| LINC01506 | 4.30358908 | ITPK1 | 1.624102942 |
| LINC00683 | 3.819404404 | ITPK1 | 1.624102942 |
| SPACA6 | 3.245939503 | SUSD6 | 1.509119903 |
| RP11-651L5.2 | 2.553017732 | SUSD6 | 1.509119903 |
| CTB-41I6.1 | 2.338928111 | NFKBIA | 2.563494995 |
| RP11-290F5.2 | 2.728772492 | RNF24 | 2.318160068 |
| LINC01359 | 2.490379801 | RNF24 | 2.318160068 |
| RP11-59D5__B.2 | 1.577814919 | RNF24 | 2.318160068 |
| LINC01513 | 3.961249217 | RNF24 | 2.318160068 |
| RP11-321E2.4 | 5.833782645 | RNF24 | 2.318160068 |
| RP11-414H23.3 | 4.982428435 | RNF24 | 2.318160068 |
| RP11-875O11.3 | 4.013157441 | RNF24 | 2.318160068 |
| RP11-619I22.1 | 2.508888219 | RNF24 | 2.318160068 |
| LA16c-380H5.5 | 2.873231121 | RNF24 | 2.318160068 |
| RP11-25K21.6 | 2.76934248 | RNF24 | 2.318160068 |
| RP11-455F5.6 | 2.421000003 | RNF24 | 2.318160068 |
| RP11-81A1.6 | 1.759373228 | RNF24 | 2.318160068 |
| RP11-408H1.3 | 2.132557425 | IRG1 | 4.769289752 |
| SMG7-AS1 | 1.621875385 | IRG1 | 4.769289752 |
| RP11-439A17.10 | 2.017936573 | IRG1 | 4.769289752 |
| RP11-434D11.4 | 3.200876885 | IRG1 | 4.769289752 |
| RP11-44K6.2 | 3.459824073 | IRG1 | 4.769289752 |
| RP11-480C16.1 | 2.105414359 | IRG1 | 4.769289752 |
| RP11-290F5.2 | 2.728772492 | CPPED1 | 2.241046922 |
| LINC01272 | 2.970869212 | CPPED1 | 2.241046922 |
| RP11-380G5.2 | 3.24768833 | CPPED1 | 2.241046922 |
| RP11-63N8.3 | 7.503029032 | CPPED1 | 2.241046922 |
| LINC01506 | 4.30358908 | CPPED1 | 2.241046922 |
| RP1-60N8.1 | 2.119294428 | CPPED1 | 2.241046922 |
| RP11-321E2.4 | 5.833782645 | CPPED1 | 2.241046922 |
| RP11-414H23.3 | 4.982428435 | CPPED1 | 2.241046922 |
| RP11-792A8.4 | 1.799510462 | CPPED1 | 2.241046922 |
| RP11-81A1.6 | 1.759373228 | CPPED1 | 2.241046922 |
| RP11-380G5.2 | 3.24768833 | DENND3 | 1.717611385 |
| RP5-968J1.1 | 3.407652425 | DENND3 | 1.717611385 |
| RP11-81A1.6 | 1.759373228 | DENND3 | 1.717611385 |
| RP11-482G13.1 | 2.681553651 | DENND3 | 1.717611385 |
| RP11-467L13.7 | 4.421343198 | SIGLEC5 | 2.874292269 |
| RP11-290F5.2 | 2.728772492 | MEGF9 | 1.599280742 |
| RP1-55C23.7 | 1.95663376 | MEGF9 | 1.599280742 |
| RP11-59D5__B.2 | 1.577814919 | MEGF9 | 1.599280742 |
| RP11-290F5.1 | 2.750305669 | MEGF9 | 1.599280742 |
| RP11-321E2.4 | 5.833782645 | MEGF9 | 1.599280742 |
| RP11-875O11.3 | 4.013157441 | MEGF9 | 1.599280742 |
| RP11-619I22.1 | 2.508888219 | MEGF9 | 1.599280742 |
| RP11-25K21.6 | 2.76934248 | MEGF9 | 1.599280742 |
| RP11-455F5.6 | 2.421000003 | MEGF9 | 1.599280742 |
| RP11-455F5.3 | 3.717449337 | MANSC1 | 4.69049132 |
| RP11-44F14.2 | 2.453885592 | MANSC1 | 4.69049132 |
| RP11-25K21.6 | 2.76934248 | MANSC1 | 4.69049132 |
| RP11-81A1.6 | 1.759373228 | MANSC1 | 4.69049132 |
| RP11-59D5__B.2 | 1.577814919 | MAK | 2.965297622 |
| RP11-321E2.4 | 5.833782645 | MAK | 2.965297622 |
| RP11-875O11.3 | 4.013157441 | MAK | 2.965297622 |
| RP11-256L6.3 | 4.089504337 | MAK | 2.965297622 |
| RP11-619I22.1 | 2.508888219 | MAK | 2.965297622 |
| LINC01270 | 5.527234914 | SLC26A8 | 4.036881847 |
| RP11-321E2.4 | 5.833782645 | SLC26A8 | 4.036881847 |
| LINC00936 | 3.079364244 | CD83 | 2.53386734 |
| RP11-63N8.3 | 7.503029032 | TREML2 | 3.042547787 |
| LINC01272 | 2.970869212 | VNN2 | 2.953787798 |
| RP11-321E2.4 | 5.833782645 | VNN2 | 2.953787798 |
| RP11-414H23.3 | 4.982428435 | VNN2 | 2.953787798 |
| RP11-256L6.3 | 4.089504337 | VNN2 | 2.953787798 |
| RP5-906C1.1 | 1.736652403 | VNN2 | 2.953787798 |
| RP11-59D5__B.2 | 1.577814919 | BTNL8 | 7.055917876 |
| RP11-321E2.4 | 5.833782645 | BTNL8 | 7.055917876 |
| RP11-455F5.3 | 3.717449337 | BTNL8 | 7.055917876 |
| RP11-290F5.2 | 2.728772492 | PFKFB4 | 1.946175748 |
| LINC01359 | 2.490379801 | PFKFB4 | 1.946175748 |
| RP11-59D5__B.2 | 1.577814919 | PFKFB4 | 1.946175748 |
| RP11-290F5.1 | 2.750305669 | PFKFB4 | 1.946175748 |
| RP11-875O11.3 | 4.013157441 | PFKFB4 | 1.946175748 |
| RP11-619I22.1 | 2.508888219 | PFKFB4 | 1.946175748 |
| LA16c-380H5.5 | 2.873231121 | PFKFB4 | 1.946175748 |
| RP11-25K21.6 | 2.76934248 | PFKFB4 | 1.946175748 |
| RP11-455F5.6 | 2.421000003 | PFKFB4 | 1.946175748 |
| RP11-81A1.6 | 1.759373228 | PFKFB4 | 1.946175748 |
| RP11-25K21.6 | 2.76934248 | ABTB1 | 1.519770358 |
| RP11-81A1.6 | 1.759373228 | ABTB1 | 1.519770358 |
| RP11-482G13.1 | 2.681553651 | ABTB1 | 1.519770358 |
| LINC01001 | 2.595275407 | KIAA1257 | 4.519538165 |
| RP11-81A1.6 | 1.759373228 | KIAA1257 | 4.519538165 |
| SMG7-AS1 | 1.621875385 | IFIH1 | 1.703188649 |
| RP13-314C10.5 | 3.987752743 | IFIH1 | 1.703188649 |
| RP11-434D11.4 | 3.200876885 | IFIH1 | 1.703188649 |
| AP000640.2 | 2.140723508 | IFIH1 | 1.703188649 |
| RP5-968J1.1 | 3.407652425 | GCA | 2.883013447 |
| RP11-875O11.3 | 4.013157441 | GCA | 2.883013447 |
| RP11-25K21.6 | 2.76934248 | GCA | 2.883013447 |
| LINC01002 | 2.871347896 | GCA | 2.883013447 |
| RP11-439L18.1 | 1.899777727 | RNF19B | 2.079590631 |
| RP11-321E2.4 | 5.833782645 | WLS | 2.694955444 |
| CTD-2313J17.5 | 1.846373622 | WLS | 2.694955444 |
| RP11-408H1.3 | 2.132557425 | GBP1 | 2.891751704 |
| SMG7-AS1 | 1.621875385 | GBP1 | 2.891751704 |
| RP13-314C10.5 | 3.987752743 | GBP1 | 2.891751704 |
| RP11-439A17.10 | 2.017936573 | GBP1 | 2.891751704 |
| RP11-434D11.4 | 3.200876885 | GBP1 | 2.891751704 |
| RP11-44K6.2 | 3.459824073 | GBP1 | 2.891751704 |
| RP11-480C16.1 | 2.105414359 | GBP1 | 2.891751704 |
| RP11-290F5.2 | 2.728772492 | TRPM6 | 2.193899874 |
| LINC01359 | 2.490379801 | TRPM6 | 2.193899874 |
| LINC01513 | 3.961249217 | TRPM6 | 2.193899874 |
| RP11-321E2.4 | 5.833782645 | TRPM6 | 2.193899874 |
| RP11-414H23.3 | 4.982428435 | TRPM6 | 2.193899874 |
| RP11-875O11.3 | 4.013157441 | TRPM6 | 2.193899874 |
| RP11-619I22.1 | 2.508888219 | TRPM6 | 2.193899874 |
| LINC01272 | 2.970869212 | HSDL2 | 1.884696497 |
| RP5-968J1.1 | 3.407652425 | HSDL2 | 1.884696497 |
| RP11-256L6.3 | 4.089504337 | HSDL2 | 1.884696497 |
| RP11-408H1.3 | 2.132557425 | IFIT3 | 3.9181574 |
| SMG7-AS1 | 1.621875385 | IFIT3 | 3.9181574 |
| RP11-439A17.10 | 2.017936573 | IFIT3 | 3.9181574 |
| RP11-434D11.4 | 3.200876885 | IFIT3 | 3.9181574 |
| RP11-44K6.2 | 3.459824073 | IFIT3 | 3.9181574 |
| RP11-480C16.1 | 2.105414359 | IFIT3 | 3.9181574 |
| RP11-408H1.3 | 2.132557425 | IFIT2 | 3.91707519 |
| SMG7-AS1 | 1.621875385 | IFIT2 | 3.91707519 |
| RP11-439A17.10 | 2.017936573 | IFIT2 | 3.91707519 |
| RP11-434D11.4 | 3.200876885 | IFIT2 | 3.91707519 |
| RP11-44K6.2 | 3.459824073 | IFIT2 | 3.91707519 |
| RP11-480C16.1 | 2.105414359 | IFIT2 | 3.91707519 |
| RP11-434D11.4 | 3.200876885 | CD274 | 1.825266795 |
| RP11-249C24.10 | 3.829348738 | CD274 | 1.825266795 |
| LINC01001 | 2.595275407 | ARAP3 | 2.278420484 |
| RP11-25K21.6 | 2.76934248 | ARAP3 | 2.278420484 |
| RP11-81A1.6 | 1.759373228 | ARAP3 | 2.278420484 |
| RP11-482G13.1 | 2.681553651 | ARAP3 | 2.278420484 |
| RP11-34F20.7 | 1.63688279 | TEX14 | 2.360347371 |
| RP11-439L18.1 | 1.899777727 | BATF3 | 2.433223883 |
| RP11-338C15.3 | 1.904273617 | BATF3 | 2.433223883 |
| PCBP1-AS1 | 2.035458499 | MBOAT7 | 2.448785032 |
| LINC01272 | 2.970869212 | MBOAT7 | 2.448785032 |
| ARHGAP26-IT1 | 2.137446203 | MBOAT7 | 2.448785032 |
| RP11-63N8.3 | 7.503029032 | MBOAT7 | 2.448785032 |
| LINC01002 | 2.871347896 | MBOAT7 | 2.448785032 |
| RP11-482G13.1 | 2.681553651 | MBOAT7 | 2.448785032 |
| AP000355.2 | 3.35116668 | IL1B | 2.823745615 |
| RP11-290F5.2 | 2.728772492 | HIP1 | 1.950257054 |
| RP11-380G5.2 | 3.24768833 | HIP1 | 1.950257054 |
| RP11-321E2.4 | 5.833782645 | HIP1 | 1.950257054 |
| RP11-875O11.3 | 4.013157441 | HIP1 | 1.950257054 |
| RP11-619I22.1 | 2.508888219 | HIP1 | 1.950257054 |
| LINC01002 | 2.871347896 | ALDH1A2 | 3.791878465 |
| LINC01272 | 2.970869212 | SEC14L1 | 2.033476254 |
| RP11-380G5.2 | 3.24768833 | SEC14L1 | 2.033476254 |
| ARHGAP26-IT1 | 2.137446203 | SEC14L1 | 2.033476254 |
| RP11-63N8.3 | 7.503029032 | SEC14L1 | 2.033476254 |
| RP11-81A1.6 | 1.759373228 | SEC14L1 | 2.033476254 |
| LINC01002 | 2.871347896 | SEC14L1 | 2.033476254 |
| RP11-482G13.1 | 2.681553651 | SEC14L1 | 2.033476254 |
| RP11-408H1.3 | 2.132557425 | IDO1 | 3.60688068 |
| SMG7-AS1 | 1.621875385 | IDO1 | 3.60688068 |
| RP11-439A17.10 | 2.017936573 | IDO1 | 3.60688068 |
| RP11-434D11.4 | 3.200876885 | IDO1 | 3.60688068 |
| RP11-44K6.2 | 3.459824073 | IDO1 | 3.60688068 |
| RP11-480C16.1 | 2.105414359 | IDO1 | 3.60688068 |
| LINC01001 | 2.595275407 | ADGRE3 | 3.600660754 |
| RP11-81A1.6 | 1.759373228 | ADGRE3 | 3.600660754 |
| RP11-482G13.1 | 2.681553651 | ADGRE3 | 3.600660754 |
| LINC01272 | 2.970869212 | SLC6A6 | 1.981496191 |
| RP5-968J1.1 | 3.407652425 | SLC6A6 | 1.981496191 |
| RP1-102E24.8 | 2.852738496 | SLC6A6 | 1.981496191 |
| RP4-647C14.3 | 2.087851208 | SLC6A6 | 1.981496191 |
| RP5-906C1.1 | 1.736652403 | SLC6A6 | 1.981496191 |
| RP11-44F14.8 | 2.459485146 | AOC3 | 3.5095305 |
| RP11-44F14.2 | 2.453885592 | AOC2 | 2.371300425 |
| RP11-44F14.8 | 2.459485146 | AOC2 | 2.371300425 |
| RP11-25K21.6 | 2.76934248 | AOC2 | 2.371300425 |
| RP11-439L18.1 | 1.899777727 | GCH1 | 1.686128027 |
| NEAT1 | 2.088068811 | PTPRE | 1.609836666 |
| CTD-2530H12.2 | 3.721723258 | KDM6B | 1.854761365 |
| RP11-568N6.1 | 1.999368955 | RIN2 | 2.11667828 |
| LINC01506 | 4.30358908 | LYVE1 | 4.827332472 |
| RP1-60N8.1 | 2.119294428 | LYVE1 | 4.827332472 |
| RP11-321E2.4 | 5.833782645 | LYVE1 | 4.827332472 |
| RP11-455F5.3 | 3.717449337 | LYVE1 | 4.827332472 |
| CTD-2313J17.5 | 1.846373622 | LYVE1 | 4.827332472 |
| RP11-619I22.1 | 2.508888219 | LYVE1 | 4.827332472 |
| RP11-455F5.6 | 2.421000003 | LYVE1 | 4.827332472 |
| RP4-647C14.3 | 2.087851208 | NUMB | 1.855702671 |
| RP11-519G16.3 | 2.432286026 | IRAK2 | 2.449126867 |
| AC012363.4 | 2.269184289 | RSAD2 | 2.950066492 |
| RP11-326C3.13 | 3.75979327 | RSAD2 | 2.950066492 |
| ARHGAP26-IT1 | 2.137446203 | PHC2 | 1.957213161 |
| RP11-63N8.3 | 7.503029032 | PHC2 | 1.957213161 |
| LINC01002 | 2.871347896 | PHC2 | 1.957213161 |
| RP1-102E24.8 | 2.852738496 | DHX34 | 2.224348336 |
| RP11-290F5.2 | 2.728772492 | CCNJL | 3.27715249 |
| RP11-81A1.6 | 1.759373228 | CCNJL | 3.27715249 |
| RP11-482G13.1 | 2.681553651 | CCNJL | 3.27715249 |
| RP1-55C23.7 | 1.95663376 | AVIL | 1.691108331 |
| RP11-59D5__B.2 | 1.577814919 | AVIL | 1.691108331 |
| RP11-875O11.3 | 4.013157441 | AVIL | 1.691108331 |
| RP11-619I22.1 | 2.508888219 | AVIL | 1.691108331 |
| RP11-455F5.6 | 2.421000003 | AVIL | 1.691108331 |
| ARHGAP26-IT1 | 2.137446203 | DYSF | 2.892061466 |
| RP11-290F5.2 | 2.728772492 | EGLN1 | 1.718865142 |
| LINC01359 | 2.490379801 | EGLN1 | 1.718865142 |
| RP1-60N8.1 | 2.119294428 | EGLN1 | 1.718865142 |
| RP11-59D5__B.2 | 1.577814919 | EGLN1 | 1.718865142 |
| LINC01513 | 3.961249217 | EGLN1 | 1.718865142 |
| RP11-321E2.4 | 5.833782645 | EGLN1 | 1.718865142 |
| RP11-414H23.3 | 4.982428435 | EGLN1 | 1.718865142 |
| RP11-875O11.3 | 4.013157441 | EGLN1 | 1.718865142 |
| RP11-256L6.3 | 4.089504337 | EGLN1 | 1.718865142 |
| RP11-619I22.1 | 2.508888219 | EGLN1 | 1.718865142 |
| RP11-455F5.6 | 2.421000003 | EGLN1 | 1.718865142 |
| RP11-81A1.6 | 1.759373228 | EGLN1 | 1.718865142 |
| LINC01272 | 2.970869212 | FAM129A | 2.447681151 |
| RP5-968J1.1 | 3.407652425 | FAM129A | 2.447681151 |
| RP11-10J21.4 | 2.837409284 | FAM129A | 2.447681151 |
| RP5-906C1.1 | 1.736652403 | FAM129A | 2.447681151 |
| LINC01002 | 2.871347896 | FAM129A | 2.447681151 |
| AP000355.2 | 3.35116668 | DRAM1 | 2.481050916 |
| KCNJ2-AS1 | 3.943652024 | DRAM1 | 2.481050916 |
| AC091878.1 | 4.178466194 | KIAA0319 | 3.373681857 |
| LINC01513 | 3.961249217 | KIAA0319 | 3.373681857 |
| RP11-875O11.3 | 4.013157441 | KIAA0319 | 3.373681857 |
| ARHGAP26-IT1 | 2.137446203 | ENTPD1 | 1.932951513 |
| RP11-429G19.3 | 2.204216233 | ENTPD1 | 1.932951513 |
| LINC01010 | 3.205609565 | PHLDA1 | 1.766917725 |
| MIR222HG | 2.929980978 | GPR84 | 2.930205288 |
| LINC01506 | 4.30358908 | REM2 | 1.915459419 |
| UBR5-AS1 | 1.601469591 | REM2 | 1.915459419 |
| RP11-321E2.4 | 5.833782645 | REM2 | 1.915459419 |
| RP11-81A1.6 | 1.759373228 | REM2 | 1.915459419 |
| NEAT1 | 2.088068811 | TLE3 | 2.045397365 |
| CFAP58-AS1 | 4.556231766 | BCL2A1 | 4.790470199 |
| CTD-2530H12.2 | 3.721723258 | ABHD2 | 1.590782197 |
| RP1-60N8.1 | 2.119294428 | DPEP3 | 2.025489772 |
| RP11-81A1.6 | 1.759373228 | DPEP3 | 2.025489772 |
| RP11-482G13.1 | 2.681553651 | DPEP3 | 2.025489772 |
| RP11-482G13.1 | 2.681553651 | SLC16A3 | 1.810669324 |
| RP11-619I22.1 | 2.508888219 | NLRP12 | 1.551449897 |
| CTD-3022G6.1 | 1.920576849 | NLRP12 | 1.551449897 |
| LA16c-380H5.5 | 2.873231121 | NLRP12 | 1.551449897 |
| CD81-AS1 | -2.11503209 | FAM71E1 | -2.218330359 |
| LINC01272 | 2.970869212 | MBOAT2 | 1.903945426 |
| RP11-380G5.2 | 3.24768833 | MBOAT2 | 1.903945426 |
| RP5-906C1.1 | 1.736652403 | MBOAT2 | 1.903945426 |
| RP11-81A1.6 | 1.759373228 | MBOAT2 | 1.903945426 |
| LINC01002 | 2.871347896 | MBOAT2 | 1.903945426 |
| RP11-153M7.5 | 4.52870836 | RALB | 1.715189044 |
| CTB-41I6.1 | 2.338928111 | NFKBIZ | 2.317376822 |
| CTA-293F17.1 | 2.745123603 | PLA1A | 2.699810627 |
| RP11-290F5.2 | 2.728772492 | ROPN1L | 3.330601711 |
| RP11-380G5.2 | 3.24768833 | ROPN1L | 3.330601711 |
| LINC01359 | 2.490379801 | ROPN1L | 3.330601711 |
| LINC01506 | 4.30358908 | ROPN1L | 3.330601711 |
| RP1-60N8.1 | 2.119294428 | ROPN1L | 3.330601711 |
| RP11-59D5__B.2 | 1.577814919 | ROPN1L | 3.330601711 |
| LINC01513 | 3.961249217 | ROPN1L | 3.330601711 |
| RP11-321E2.4 | 5.833782645 | ROPN1L | 3.330601711 |
| RP11-414H23.3 | 4.982428435 | ROPN1L | 3.330601711 |
| RP11-875O11.3 | 4.013157441 | ROPN1L | 3.330601711 |
| RP11-256L6.3 | 4.089504337 | ROPN1L | 3.330601711 |
| RP11-619I22.1 | 2.508888219 | ROPN1L | 3.330601711 |
| RP11-455F5.6 | 2.421000003 | ROPN1L | 3.330601711 |
| RP11-81A1.6 | 1.759373228 | ROPN1L | 3.330601711 |
| ARHGAP26-IT1 | 2.137446203 | ARHGAP26 | 1.538141985 |
| RP11-321E2.4 | 5.833782645 | CREB5 | 2.824466027 |
| RP11-875O11.3 | 4.013157441 | CREB5 | 2.824466027 |
| RP11-619I22.1 | 2.508888219 | CREB5 | 2.824466027 |
| RP5-906C1.1 | 1.736652403 | SLC25A37 | 3.354393625 |
| ARHGAP26-IT1 | 2.137446203 | DOCK5 | 2.763910307 |
| LINC01002 | 2.871347896 | DOCK5 | 2.763910307 |
| RP11-482G13.1 | 2.681553651 | NR6A1 | 2.093672726 |
| RP11-274B21.10 | 1.589418879 | NOTCH1 | 1.537544582 |
| RP11-81A1.6 | 1.759373228 | NOTCH1 | 1.537544582 |
| RP11-482G13.1 | 2.681553651 | NOTCH1 | 1.537544582 |
| SPACA6 | 3.245939503 | ADAM8 | 1.773987701 |
| CTD-2530H12.2 | 3.721723258 | ADAM8 | 1.773987701 |
| RP11-351I24.1 | 3.345387839 | ACSL1 | 3.92117258 |
| LINC01272 | 2.970869212 | GLT1D1 | 3.030204561 |
| RP11-380G5.2 | 3.24768833 | GLT1D1 | 3.030204561 |
| ARHGAP26-IT1 | 2.137446203 | GLT1D1 | 3.030204561 |
| RP5-968J1.1 | 3.407652425 | GLT1D1 | 3.030204561 |
| RP11-63N8.3 | 7.503029032 | GLT1D1 | 3.030204561 |
| RP5-906C1.1 | 1.736652403 | GLT1D1 | 3.030204561 |
| RP11-81A1.6 | 1.759373228 | GLT1D1 | 3.030204561 |
| LINC01002 | 2.871347896 | GLT1D1 | 3.030204561 |
| RP11-482G13.1 | 2.681553651 | GLT1D1 | 3.030204561 |
| NEAT1 | 2.088068811 | FAM49B | 1.571698625 |
| AC012363.4 | 2.269184289 | GBP5 | 2.189595153 |
| RP13-314C10.5 | 3.987752743 | GBP5 | 2.189595153 |
| RP11-434D11.4 | 3.200876885 | GBP5 | 2.189595153 |
| LINC01272 | 2.970869212 | EPHB1 | 4.670762659 |
| RP11-380G5.2 | 3.24768833 | EPHB1 | 4.670762659 |
| LINC01506 | 4.30358908 | EPHB1 | 4.670762659 |
| LINC01002 | 2.871347896 | EPHB1 | 4.670762659 |
| RP11-380G5.2 | 3.24768833 | KCNJ15 | 7.094080523 |
| ARHGAP26-IT1 | 2.137446203 | KCNJ15 | 7.094080523 |
| RP11-63N8.3 | 7.503029032 | KCNJ15 | 7.094080523 |
| RP11-321E2.4 | 5.833782645 | KCNJ15 | 7.094080523 |
| LINC01002 | 2.871347896 | KCNJ15 | 7.094080523 |
| AC091878.1 | 4.178466194 | GALNT14 | 3.155904689 |
| RP11-44F14.2 | 2.453885592 | FBXL13 | 2.927217982 |
| LINC01272 | 2.970869212 | ALPL | 7.590432373 |
| KLF3-AS1 | -1.601962028 | MXRA8 | -2.008849429 |
| CFAP58-AS1 | 4.556231766 | GBP2 | 2.470117777 |
| RP11-380G5.2 | 3.24768833 | FCGR3B | 8.058109236 |
| LINC01506 | 4.30358908 | FCGR3B | 8.058109236 |
| RP11-249C24.10 | 3.829348738 | FAM71A | 3.198590866 |
| LINC01270 | 5.527234914 | RNF149 | 1.731362055 |
| ARHGAP26-IT1 | 2.137446203 | RNF149 | 1.731362055 |
| RP11-63N8.3 | 7.503029032 | RNF149 | 1.731362055 |
| RP11-59D5__B.2 | 1.577814919 | TGFA | 2.768276932 |
| RP11-321E2.4 | 5.833782645 | TGFA | 2.768276932 |
| RP11-875O11.3 | 4.013157441 | TGFA | 2.768276932 |
| RP11-619I22.1 | 2.508888219 | TGFA | 2.768276932 |
| LA16c-380H5.5 | 2.873231121 | TGFA | 2.768276932 |
| RP11-455F5.6 | 2.421000003 | TGFA | 2.768276932 |
| RP11-59D5__B.2 | 1.577814919 | CXCR1 | 5.372775601 |
| RP11-321E2.4 | 5.833782645 | CXCR1 | 5.372775601 |
| RP11-875O11.3 | 4.013157441 | CXCR1 | 5.372775601 |
| RP11-619I22.1 | 2.508888219 | CXCR1 | 5.372775601 |
| LINC01506 | 4.30358908 | MNDA | 2.391314764 |
| RP11-256L6.3 | 4.089504337 | MNDA | 2.391314764 |
| LINC01272 | 2.970869212 | WDFY3 | 2.508755728 |
| ARHGAP26-IT1 | 2.137446203 | WDFY3 | 2.508755728 |
| RP11-63N8.3 | 7.503029032 | WDFY3 | 2.508755728 |
| LINC01002 | 2.871347896 | WDFY3 | 2.508755728 |
| ARHGAP26-IT1 | 2.137446203 | RBM47 | 2.395088256 |
| RP4-647C14.3 | 2.087851208 | RBM47 | 2.395088256 |
| HRAT92 | -1.945763175 | PF4 | -1.549521724 |
| RP11-321E2.4 | 5.833782645 | ARFIP1 | 1.620732344 |
| RP11-875O11.3 | 4.013157441 | ARFIP1 | 1.620732344 |
| SMG7-AS1 | 1.621875385 | RANBP3L | 2.176110872 |
| RP11-21C4.1 | 1.972971028 | RANBP3L | 2.176110872 |
| RP11-569A11.1 | 1.960953633 | F2RL1 | 3.09195379 |
| LINC01359 | 2.490379801 | F2RL1 | 3.09195379 |
| RP11-59D5__B.2 | 1.577814919 | F2RL1 | 3.09195379 |
| RP11-290F5.1 | 2.750305669 | F2RL1 | 3.09195379 |
| RP11-875O11.3 | 4.013157441 | F2RL1 | 3.09195379 |
| RP11-44F14.2 | 2.453885592 | F2RL1 | 3.09195379 |
| RP11-619I22.1 | 2.508888219 | F2RL1 | 3.09195379 |
| LA16c-380H5.5 | 2.873231121 | F2RL1 | 3.09195379 |
| RP4-535B20.4 | 2.526351518 | F2RL1 | 3.09195379 |
| RP11-25K21.6 | 2.76934248 | F2RL1 | 3.09195379 |
| RP11-455F5.6 | 2.421000003 | F2RL1 | 3.09195379 |
| RP11-81A1.6 | 1.759373228 | F2RL1 | 3.09195379 |
| LINC01272 | 2.970869212 | STX3 | 2.83149089 |
| RP11-380G5.2 | 3.24768833 | STX3 | 2.83149089 |
| ARHGAP26-IT1 | 2.137446203 | STX3 | 2.83149089 |
| RP5-968J1.1 | 3.407652425 | STX3 | 2.83149089 |
| LINC01002 | 2.871347896 | STX3 | 2.83149089 |
| RP11-568N6.1 | 1.999368955 | C15orf48 | 2.650042039 |
| ADAMTSL4-AS1 | 2.643346202 | C15orf39 | 1.707211601 |
| PCBP1-AS1 | 2.035458499 | PLIN4 | 4.322305319 |
| SPACA6 | 3.245939503 | PLIN4 | 4.322305319 |
| WDR11-AS1 | -1.558084769 | SDPR | -1.68280989 |
| AP001189.4 | -2.058389114 | SDPR | -1.68280989 |
| RP11-380G5.2 | 3.24768833 | XPO6 | 1.916644893 |
| ARHGAP26-IT1 | 2.137446203 | XPO6 | 1.916644893 |
| RP11-63N8.3 | 7.503029032 | XPO6 | 1.916644893 |
| RP5-906C1.1 | 1.736652403 | XPO6 | 1.916644893 |
| RP11-81A1.6 | 1.759373228 | XPO6 | 1.916644893 |
| LINC01002 | 2.871347896 | XPO6 | 1.916644893 |
| RP11-408H1.3 | 2.132557425 | CXCL10 | 3.216219575 |
| SMG7-AS1 | 1.621875385 | CXCL10 | 3.216219575 |
| RP11-439A17.10 | 2.017936573 | CXCL10 | 3.216219575 |
| RP11-434D11.4 | 3.200876885 | CXCL10 | 3.216219575 |
| RP11-44K6.2 | 3.459824073 | CXCL10 | 3.216219575 |
| RP11-249C24.10 | 3.829348738 | CXCL10 | 3.216219575 |
| RP11-480C16.1 | 2.105414359 | CXCL10 | 3.216219575 |
| LINC01272 | 2.970869212 | REPS2 | 2.542966393 |
| RP5-968J1.1 | 3.407652425 | REPS2 | 2.542966393 |
| UBR5-AS1 | 1.601469591 | REPS2 | 2.542966393 |
| RP11-875O11.3 | 4.013157441 | REPS2 | 2.542966393 |
| RP5-906C1.1 | 1.736652403 | REPS2 | 2.542966393 |
| LINC01002 | 2.871347896 | REPS2 | 2.542966393 |
| RP11-290F5.2 | 2.728772492 | TMEM154 | 2.211020226 |
| RP11-380G5.2 | 3.24768833 | TMEM154 | 2.211020226 |
| UBR5-AS1 | 1.601469591 | TMEM154 | 2.211020226 |
| RP11-321E2.4 | 5.833782645 | TMEM154 | 2.211020226 |
| RP11-875O11.3 | 4.013157441 | TMEM154 | 2.211020226 |
| RP11-256L6.3 | 4.089504337 | TMEM154 | 2.211020226 |
| RP11-619I22.1 | 2.508888219 | TMEM154 | 2.211020226 |
| RP11-25K21.6 | 2.76934248 | TMEM154 | 2.211020226 |
| LINC01272 | 2.970869212 | USP32 | 1.782152576 |
| RP11-380G5.2 | 3.24768833 | USP32 | 1.782152576 |
| ARHGAP26-IT1 | 2.137446203 | USP32 | 1.782152576 |
| RP11-10J21.4 | 2.837409284 | USP32 | 1.782152576 |
| RP5-906C1.1 | 1.736652403 | USP32 | 1.782152576 |
| LINC01002 | 2.871347896 | USP32 | 1.782152576 |
| RP1-60N8.1 | 2.119294428 | GPR27 | 2.265469673 |
| RP11-81A1.6 | 1.759373228 | GPR27 | 2.265469673 |
| RP11-290F5.2 | 2.728772492 | TSEN34 | 1.534334958 |
| RP11-59D5__B.2 | 1.577814919 | TSEN34 | 1.534334958 |
| RP11-321E2.4 | 5.833782645 | TSEN34 | 1.534334958 |
| RP11-414H23.3 | 4.982428435 | TSEN34 | 1.534334958 |
| RP11-875O11.3 | 4.013157441 | TSEN34 | 1.534334958 |
| RP11-256L6.3 | 4.089504337 | TSEN34 | 1.534334958 |
| RP11-619I22.1 | 2.508888219 | TSEN34 | 1.534334958 |
| LA16c-380H5.5 | 2.873231121 | TSEN34 | 1.534334958 |
| RP11-25K21.6 | 2.76934248 | TSEN34 | 1.534334958 |
| RP11-81A1.6 | 1.759373228 | TSEN34 | 1.534334958 |
| LINC01272 | 2.970869212 | RASGRP4 | 2.17929539 |
| LINC01001 | 2.595275407 | RASGRP4 | 2.17929539 |
| ARHGAP26-IT1 | 2.137446203 | RASGRP4 | 2.17929539 |
| RP5-968J1.1 | 3.407652425 | RASGRP4 | 2.17929539 |
| RP11-63N8.3 | 7.503029032 | RASGRP4 | 2.17929539 |
| RP5-906C1.1 | 1.736652403 | RASGRP4 | 2.17929539 |
| RP11-81A1.6 | 1.759373228 | RASGRP4 | 2.17929539 |
| LINC01002 | 2.871347896 | RASGRP4 | 2.17929539 |
| RP11-482G13.1 | 2.681553651 | RASGRP4 | 2.17929539 |
| RP11-380G5.2 | 3.24768833 | PTEN | 1.943622442 |
| LINC01513 | 3.961249217 | PDZD3 | 1.534481861 |
| RP11-59D5__B.2 | 1.577814919 | HSPA6 | 3.21700658 |
| LINC01513 | 3.961249217 | HSPA6 | 3.21700658 |
| RP11-321E2.4 | 5.833782645 | HSPA6 | 3.21700658 |
| RP11-875O11.3 | 4.013157441 | HSPA6 | 3.21700658 |
| RP11-619I22.1 | 2.508888219 | HSPA6 | 3.21700658 |
| LA16c-380H5.5 | 2.873231121 | HSPA6 | 3.21700658 |
| RP11-455F5.6 | 2.421000003 | HSPA6 | 3.21700658 |
| AC091878.1 | 4.178466194 | MAB21L3 | 3.772234674 |
| RP1-102E24.8 | 2.852738496 | PPP1R3B | 2.462920251 |
| RP5-906C1.1 | 1.736652403 | PPP1R3B | 2.462920251 |
| SPACA6 | 3.245939503 | MAP3K11 | 1.52626031 |
| LINC01136 | 3.525823875 | MAP3K11 | 1.52626031 |
| MIR22HG | 2.229990009 | TRIB1 | 3.4176928 |
| RP11-398A8.5 | 2.528651054 | KCNK7 | 2.984389434 |
| RP11-561B11.3 | 3.70775371 | THAP2 | 2.070495655 |
| RP11-290F5.2 | 2.728772492 | TNFRSF10C | 5.252756797 |
| RP11-380G5.2 | 3.24768833 | TNFRSF10C | 5.252756797 |
| LINC01359 | 2.490379801 | TNFRSF10C | 5.252756797 |
| RP3-393E18.2 | 2.168339529 | TNFRSF10C | 5.252756797 |
| RP11-290F5.1 | 2.750305669 | TNFRSF10C | 5.252756797 |
| RP11-321E2.4 | 5.833782645 | TNFRSF10C | 5.252756797 |
| RP11-414H23.3 | 4.982428435 | TNFRSF10C | 5.252756797 |
| RP11-256L6.3 | 4.089504337 | TNFRSF10C | 5.252756797 |
| RP11-619I22.1 | 2.508888219 | TNFRSF10C | 5.252756797 |
| RP11-81A1.6 | 1.759373228 | TNFRSF10C | 5.252756797 |
| RP11-556I13.2 | 2.868948148 | SULT1B1 | 1.762616793 |
| LINC01127 | 2.129490924 | SULT1B1 | 1.762616793 |
| RP11-81A1.6 | 1.759373228 | SLC19A1 | 2.353333309 |
| RP11-482G13.1 | 2.681553651 | SLC19A1 | 2.353333309 |
| RP11-569A11.1 | 1.960953633 | TIGD3 | 1.948226655 |
| RP11-455F5.3 | 3.717449337 | TIGD3 | 1.948226655 |
| RP11-81A1.6 | 1.759373228 | TIGD3 | 1.948226655 |
| RP11-7F17.3 | 5.035491078 | PHOSPHO1 | 3.651539719 |
| RP11-59D5__B.2 | 1.577814919 | CEP19 | 2.531890914 |
| LINC01513 | 3.961249217 | CEP19 | 2.531890914 |
| RP11-321E2.4 | 5.833782645 | CEP19 | 2.531890914 |
| RP11-875O11.3 | 4.013157441 | CEP19 | 2.531890914 |
| CTD-2313J17.5 | 1.846373622 | CEP19 | 2.531890914 |
| RP11-619I22.1 | 2.508888219 | CEP19 | 2.531890914 |
| RP4-535B20.4 | 2.526351518 | CEP19 | 2.531890914 |
| RP11-455F5.6 | 2.421000003 | CEP19 | 2.531890914 |
| LA16c-380H5.5 | 2.873231121 | TLR1 | 2.207949959 |
| RP5-968J1.1 | 3.407652425 | TLR6 | 2.25639959 |
| RP11-290F5.1 | 2.750305669 | TLR6 | 2.25639959 |
| RP11-875O11.3 | 4.013157441 | TLR6 | 2.25639959 |
| RP11-44F14.2 | 2.453885592 | TLR6 | 2.25639959 |
| RP11-619I22.1 | 2.508888219 | TLR6 | 2.25639959 |
| LA16c-380H5.5 | 2.873231121 | TLR6 | 2.25639959 |
| RP11-25K21.6 | 2.76934248 | TLR6 | 2.25639959 |
| RP11-455F5.6 | 2.421000003 | TLR6 | 2.25639959 |
| RP11-290F5.2 | 2.728772492 | KY | 4.281695569 |
| RP1-60N8.1 | 2.119294428 | KY | 4.281695569 |
| RP11-290F5.1 | 2.750305669 | KY | 4.281695569 |
| RP11-619I22.1 | 2.508888219 | KY | 4.281695569 |
| RP11-455F5.6 | 2.421000003 | KY | 4.281695569 |
| RP11-81A1.6 | 1.759373228 | KY | 4.281695569 |
| RP11-875O11.3 | 4.013157441 | SLC22A1 | 2.435372138 |
| RP11-81A1.6 | 1.759373228 | SLC22A1 | 2.435372138 |
| LINC00612 | -2.640333608 | A2M | -1.842528858 |
| LA16c-380H5.5 | 2.873231121 | FUT7 | 1.821111224 |
| RP11-290F5.2 | 2.728772492 | CXCR2 | 5.409647737 |
| LINC01506 | 4.30358908 | CXCR2 | 5.409647737 |
| RP11-59D5__B.2 | 1.577814919 | CXCR2 | 5.409647737 |
| UBR5-AS1 | 1.601469591 | CXCR2 | 5.409647737 |
| RP11-321E2.4 | 5.833782645 | CXCR2 | 5.409647737 |
| RP11-455F5.3 | 3.717449337 | CXCR2 | 5.409647737 |
| RP11-875O11.3 | 4.013157441 | CXCR2 | 5.409647737 |
| RP11-256L6.3 | 4.089504337 | CXCR2 | 5.409647737 |
| RP11-619I22.1 | 2.508888219 | CXCR2 | 5.409647737 |
| RP11-81A1.6 | 1.759373228 | CXCR2 | 5.409647737 |
| RP11-290F5.2 | 2.728772492 | ST20 | 2.639676046 |
| RP11-290F5.1 | 2.750305669 | ST20 | 2.639676046 |
| RP11-455F5.3 | 3.717449337 | ST20 | 2.639676046 |
| RP11-44F14.2 | 2.453885592 | ST20 | 2.639676046 |
| RP11-619I22.1 | 2.508888219 | ST20 | 2.639676046 |
| RP11-25K21.6 | 2.76934248 | ST20 | 2.639676046 |
| RP11-81A1.6 | 1.759373228 | ST20 | 2.639676046 |
| LINC01506 | 4.30358908 | LSMEM1 | 2.036019473 |
| RP11-290F5.2 | 2.728772492 | P2RY13 | 2.068593398 |
| LINC01359 | 2.490379801 | P2RY13 | 2.068593398 |
| RP11-59D5__B.2 | 1.577814919 | P2RY13 | 2.068593398 |
| LINC01513 | 3.961249217 | P2RY13 | 2.068593398 |
| RP11-290F5.1 | 2.750305669 | P2RY13 | 2.068593398 |
| RP11-875O11.3 | 4.013157441 | P2RY13 | 2.068593398 |
| RP11-619I22.1 | 2.508888219 | P2RY13 | 2.068593398 |
| LA16c-380H5.5 | 2.873231121 | P2RY13 | 2.068593398 |
| RP4-535B20.4 | 2.526351518 | P2RY13 | 2.068593398 |
| RP11-455F5.6 | 2.421000003 | P2RY13 | 2.068593398 |
| RP11-640L9.1 | 1.795291487 | P2RY13 | 2.068593398 |
| RP11-290F5.1 | 2.750305669 | OR52K2 | 2.215885978 |
| RP11-619I22.1 | 2.508888219 | OR52K2 | 2.215885978 |
| RP5-968J1.1 | 3.407652425 | CHST15 | 2.402367263 |
| PCBP1-AS1 | 2.035458499 | ADGRG3 | 4.349664097 |
| RP11-54A4.2 | 2.957030215 | ADGRG3 | 4.349664097 |
| CTD-2530H12.2 | 3.721723258 | ADGRG3 | 4.349664097 |
| RP11-44F14.2 | 2.453885592 | CEP63 | 1.521477207 |
| RP11-561P12.5 | 5.669433471 | MX2 | 1.830840007 |
| SRGAP2-AS1 | 1.544009117 | PRR16 | 1.604187552 |
| RP11-338C15.3 | 1.904273617 | PRR16 | 1.604187552 |
| RP11-568N6.1 | 1.999368955 | PRR16 | 1.604187552 |
| UBR5-AS1 | 1.601469591 | KIAA0825 | 1.933908273 |
| RP11-875O11.3 | 4.013157441 | KIAA0825 | 1.933908273 |
| RP11-25K21.6 | 2.76934248 | KIAA0825 | 1.933908273 |
| U73166.2 | 2.490598265 | C7orf61 | 2.301642193 |
| LINC01506 | 4.30358908 | CYP4F12 | 3.928457877 |
| RP11-455F5.3 | 3.717449337 | CYP4F12 | 3.928457877 |
| RP11-81A1.6 | 1.759373228 | CYP4F12 | 3.928457877 |
| CTB-61M7.2 | 3.382587578 | FCAR | 3.289731726 |
| RP11-25K21.6 | 2.76934248 | CYP4F3 | 5.359058871 |
| RP11-81A1.6 | 1.759373228 | CYP4F3 | 5.359058871 |
| LINC01272 | 2.970869212 | C3orf62 | 1.953186362 |
| ARHGAP26-IT1 | 2.137446203 | C3orf62 | 1.953186362 |
| RP11-63N8.3 | 7.503029032 | C3orf62 | 1.953186362 |
| RP11-81A1.6 | 1.759373228 | C3orf62 | 1.953186362 |
| LINC01002 | 2.871347896 | C3orf62 | 1.953186362 |
| RP11-482G13.1 | 2.681553651 | C3orf62 | 1.953186362 |
| RP11-249C24.10 | 3.829348738 | FAM72B | 1.842751291 |
| RP11-290F5.2 | 2.728772492 | LRRK2 | 2.6119394 |
| RP11-380G5.2 | 3.24768833 | LRRK2 | 2.6119394 |
| RP11-290F5.1 | 2.750305669 | LRRK2 | 2.6119394 |
| RP11-321E2.4 | 5.833782645 | LRRK2 | 2.6119394 |
| RP11-414H23.3 | 4.982428435 | LRRK2 | 2.6119394 |
| RP11-875O11.3 | 4.013157441 | LRRK2 | 2.6119394 |
| RP11-256L6.3 | 4.089504337 | LRRK2 | 2.6119394 |
| RP11-619I22.1 | 2.508888219 | LRRK2 | 2.6119394 |
| RP11-25K21.6 | 2.76934248 | LRRK2 | 2.6119394 |
| RP11-455F5.6 | 2.421000003 | LRRK2 | 2.6119394 |
| RP11-81A1.6 | 1.759373228 | LRRK2 | 2.6119394 |
| SPACA6 | 3.245939503 | LITAF | 1.626973145 |
| RP11-153M7.5 | 4.52870836 | LITAF | 1.626973145 |
| LINC01270 | 5.527234914 | IL1RAP | 2.827660765 |
| NEAT1 | 2.088068811 | STK40 | 1.727701663 |
| CTD-2530H12.2 | 3.721723258 | STK40 | 1.727701663 |
| RP5-968J1.1 | 3.407652425 | MME | 6.07344081 |
| UBR5-AS1 | 1.601469591 | MME | 6.07344081 |
| RP11-44F14.2 | 2.453885592 | MME | 6.07344081 |
| RP11-25K21.6 | 2.76934248 | MME | 6.07344081 |
| RP11-81A1.6 | 1.759373228 | MME | 6.07344081 |
| LINC01272 | 2.970869212 | TECPR2 | 2.738337172 |
| ARHGAP26-IT1 | 2.137446203 | TECPR2 | 2.738337172 |
| RP5-968J1.1 | 3.407652425 | TECPR2 | 2.738337172 |
| RP11-63N8.3 | 7.503029032 | TECPR2 | 2.738337172 |
| RP5-906C1.1 | 1.736652403 | TECPR2 | 2.738337172 |
| RP11-81A1.6 | 1.759373228 | TECPR2 | 2.738337172 |
| LINC01002 | 2.871347896 | TECPR2 | 2.738337172 |
| RP11-482G13.1 | 2.681553651 | TECPR2 | 2.738337172 |
| RP11-290F5.2 | 2.728772492 | IGF2R | 1.726291177 |
| ARHGAP26-IT1 | 2.137446203 | IGF2R | 1.726291177 |
| RP11-63N8.3 | 7.503029032 | IGF2R | 1.726291177 |
| LINC01506 | 4.30358908 | IGF2R | 1.726291177 |
| RP11-81A1.6 | 1.759373228 | IGF2R | 1.726291177 |
| LINC01002 | 2.871347896 | IGF2R | 1.726291177 |
| ARHGAP26-IT1 | 2.137446203 | SLC22A4 | 2.626335893 |
| RP5-906C1.1 | 1.736652403 | SLC22A4 | 2.626335893 |
| LINC01002 | 2.871347896 | SLC22A4 | 2.626335893 |
| RP11-408H1.3 | 2.132557425 | IL27 | 1.572968328 |
| RP11-434D11.4 | 3.200876885 | IL27 | 1.572968328 |
| RP11-44K6.2 | 3.459824073 | IL27 | 1.572968328 |
| RP11-480C16.1 | 2.105414359 | IL27 | 1.572968328 |
| RP11-63N8.3 | 7.503029032 | LRP10 | 1.500061315 |
| RP11-81A1.6 | 1.759373228 | LRP10 | 1.500061315 |
| RP11-290F5.2 | 2.728772492 | FAM212B | 2.755861813 |
| RP11-569A11.1 | 1.960953633 | FAM212B | 2.755861813 |
| LINC01359 | 2.490379801 | FAM212B | 2.755861813 |
| RP11-59D5__B.2 | 1.577814919 | FAM212B | 2.755861813 |
| RP3-393E18.2 | 2.168339529 | FAM212B | 2.755861813 |
| LINC01513 | 3.961249217 | FAM212B | 2.755861813 |
| RP11-290F5.1 | 2.750305669 | FAM212B | 2.755861813 |
| RP11-321E2.4 | 5.833782645 | FAM212B | 2.755861813 |
| RP11-414H23.3 | 4.982428435 | FAM212B | 2.755861813 |
| RP11-875O11.3 | 4.013157441 | FAM212B | 2.755861813 |
| RP11-619I22.1 | 2.508888219 | FAM212B | 2.755861813 |
| LA16c-380H5.5 | 2.873231121 | FAM212B | 2.755861813 |
| RP4-535B20.4 | 2.526351518 | FAM212B | 2.755861813 |
| RP11-455F5.6 | 2.421000003 | FAM212B | 2.755861813 |
| RP11-81A1.6 | 1.759373228 | FAM212B | 2.755861813 |
| RP11-455F5.3 | 3.717449337 | MPZL1 | 1.908665919 |
| RP11-875O11.3 | 4.013157441 | MPZL1 | 1.908665919 |
| RP11-81A1.6 | 1.759373228 | MPZL1 | 1.908665919 |
| RP11-290F5.2 | 2.728772492 | MSRB1 | 2.828009602 |
| RP11-380G5.2 | 3.24768833 | MSRB1 | 2.828009602 |
| AP001434.2 | 3.372630507 | MSRB1 | 2.828009602 |
| RP11-875O11.3 | 4.013157441 | MSRB1 | 2.828009602 |
| RP11-619I22.1 | 2.508888219 | MSRB1 | 2.828009602 |
| RP11-25K21.6 | 2.76934248 | MSRB1 | 2.828009602 |
| RP11-455F5.6 | 2.421000003 | MSRB1 | 2.828009602 |
| RP11-81A1.6 | 1.759373228 | MSRB1 | 2.828009602 |
| LINC01002 | 2.871347896 | MSRB1 | 2.828009602 |
| RP11-482G13.1 | 2.681553651 | MSRB1 | 2.828009602 |
| RP11-242C19.2 | 3.011775054 | GK | 2.954262082 |
| RP11-153M7.5 | 4.52870836 | GK | 2.954262082 |
| LINC01272 | 2.970869212 | CR1 | 1.97930237 |
| RP5-968J1.1 | 3.407652425 | CR1 | 1.97930237 |
| PCBP1-AS1 | 2.035458499 | ZDHHC18 | 2.498451789 |
| RP11-10J21.4 | 2.837409284 | ZDHHC18 | 2.498451789 |
| RP11-568N6.1 | 1.999368955 | CARD16 | 2.008394357 |
| RP11-182J23.1 | 2.87482339 | PLIN5 | 4.458726368 |
| FAM157C | 2.624311882 | PLIN5 | 4.458726368 |
| RP11-81A1.6 | 1.759373228 | PLIN5 | 4.458726368 |
| RP11-482G13.1 | 2.681553651 | PLIN5 | 4.458726368 |
| PCBP1-AS1 | 2.035458499 | C10orf105 | 3.10971181 |
| ARHGAP26-IT1 | 2.137446203 | UBXN2B | 1.691718342 |
| RP11-63N8.3 | 7.503029032 | UBXN2B | 1.691718342 |
| RP11-256L6.3 | 4.089504337 | LINC00694 | 2.766620523 |
| RP11-34F20.7 | 1.63688279 | TNF | 2.37597135 |
| LINC01272 | 2.970869212 | NFAM1 | 1.780430868 |
| LINC01001 | 2.595275407 | NFAM1 | 1.780430868 |
| ARHGAP26-IT1 | 2.137446203 | NFAM1 | 1.780430868 |
| RP5-968J1.1 | 3.407652425 | NFAM1 | 1.780430868 |
| RP11-63N8.3 | 7.503029032 | NFAM1 | 1.780430868 |
| RP11-81A1.6 | 1.759373228 | NFAM1 | 1.780430868 |
| LINC01002 | 2.871347896 | NFAM1 | 1.780430868 |
| RP11-482G13.1 | 2.681553651 | NFAM1 | 1.780430868 |
| LINC01359 | 2.490379801 | PTTG2 | 2.146983366 |
| LINC01513 | 3.961249217 | PTTG2 | 2.146983366 |
| RP11-875O11.3 | 4.013157441 | PTTG2 | 2.146983366 |
| RP11-619I22.1 | 2.508888219 | PTTG2 | 2.146983366 |
| LA16c-380H5.5 | 2.873231121 | PTTG2 | 2.146983366 |
| LLNLR-470E3.1 | 4.535980651 | SIGLEC14 | 5.946094566 |
| LINC01506 | 4.30358908 | AP5B1 | 1.668639017 |
| SMG7-AS1 | 1.621875385 | CARD17 | 2.751430924 |
| RP13-314C10.5 | 3.987752743 | CARD17 | 2.751430924 |
| AP000640.2 | 2.140723508 | CARD17 | 2.751430924 |
| RP5-968J1.1 | 3.407652425 | MGAM | 5.342974015 |
| AP001434.2 | 3.372630507 | CTB-50L17.14 | 4.465359603 |
| LINC01093 | 5.160001906 | CCL4 | 2.653972725 |
| KCNJ2-AS1 | 3.943652024 | CCL4 | 2.653972725 |

## Table S6. The 65 miRNAs targeting to 38 DEGs

| **miRNA** | **Classification** | **Reported COVID-19 related genes** | **Expression pattern** | **LTPP/HC** | **LTPP/RP** | **PMID or DOI** |
| --- | --- | --- | --- | --- | --- | --- |
| hsa-let-7c-5p | pro-inflammatory | √ |  | -0.930262 | -1.06329 | 10.1101/2021.06.08.21258565 |
| hsa-let-7b-3p | pro-inflammatory | √ |  | -0.540229 | -1.10518 | 29657261, 32489698 |
| hsa-miR-128-1-5p | anti-virus | √ | LHH | -1.24733 | -1.10285 | 34198800 |
| hsa-miR-450a-2-3p | anti-inflammatory | √ |  | 1.410609 | -1.13152 | 34456603 |
| hsa-miR-149-5p | anti-inflammatory | √ | LHH | -1.45126 | -1.56675 | 35286916, 32992681 |
| hsa-miR-122-5p | anti-inflammatory | √ |  | -4.26772 | -6.77748 | 34150006, 33801496 |
| hsa-miR-127-3p | anti-inflammatory | √ | LMH | -7.55402 | -3.56957 | 10.52155 |
| hsa-miR-23a-3p | anti-inflammatory | √ | LHH | -4.2418 | -4.82995 | 35064006 |
| hsa-miR-2355-5p | anti-inflammatory | √ |  | -3.96844 | -5.23949 | 33246355 |
| hsa-miR-338-5p |  | √ |  | 1.479783 | -1.07434 | 34935057 |
| hsa-miR-3605-3p |  | √ | LHH | -2.35543 | -2.11711 | 35464324 |
| hsa-miR-4781-3p |  | √ | LHH | -1.4174 | -1.08836 | 33246355 |
| hsa-miR-193a-5p |  | √ |  | 1.109646 | -2.20676 | 33246355 |
| hsa-miR-148a-3p |  | √ | LHH | -4.24711 | -3.44339 | 34935057 |
| hsa-miR-3150b-3p |  | √ |  | -1.59292 | -3.77561 | 35130828 |
| hsa-miR-424-3p | pro-inflammatory |  |  | 0.1887387 | -1.26118 | 26283876 |
| hsa-miR-143-3p | pro-inflammatory |  |  | -1.17443 | -2.39886 | 32597476 |
| hsa-miR-589-5p | pro-inflammatory |  | LHH | -2.27639 | -1.27829 | 32551232 |
| hsa-miR-129-5p | pro-inflammatory |  |  | -1.77466 | -4.32113 | 34778251 |
| hsa-miR-30e-3p | anti-virus |  |  | -0.974224 | -1.38255 | 32878461 |
| hsa-miR-147b-3p | anti-inflammatory |  | LHH | -1.45134 | -2.02377 | 34878835 |
| hsa-miR-197-3p | anti-inflammatory |  |  | -0.318501 | -1.08817 | 33436947 |
| hsa-miR-128-3p | anti-inflammatory |  | LMH | -3.282 | -1.33966 | 32830547 |
| hsa-miR-1298-5p | anti-inflammatory |  | LHH | -2.37755 | -2.30029 | 33469358 |
| hsa-miR-194-3p | anti-inflammatory |  |  | 1.464136 | -1.02997 | 10.1158/1538-7445.AM2016-1113 |
| hsa-miR-4796-3p | anti-inflammatory |  |  | -0.618963 | -1.38634 | 33045840 |
| hsa-miR-576-3p | anti-inflammatory |  | LHH | -2.08343 | -1.62672 | 25232931 |
| hsa-miR-7-5p | anti-inflammatory |  |  | -0.346646 | -1.10297 | 27203220 |
| hsa-miR-550a-3-5p | anti-inflammatory |  |  | -0.381999 | -1.70035 | 25293367 |
| hsa-miR-1288-3p |  |  | LMH | -4.81824 | -2.23941 |  |
| hsa-miR-27a-5p |  |  |  | -0.557483 | -1.10276 |  |
| hsa-miR-433-3p |  |  | LMH | -5.7873 | -3.05068 |  |
| hsa-miR-4677-5p |  |  | LHH | -2.63213 | -2.02422 |  |
| hsa-miR-548o-3p |  |  | LMH | -3.6433 | -2.25645 |  |
| hsa-miR-552-3p |  |  | LMH | -4.09065 | -2.24953 |  |
| hsa-miR-6516-3p |  |  | LHH | -1.88445 | -2.14585 |  |
| hsa-miR-6806-3p |  |  | LMH | -2.9824 | -1.71487 |  |
| hsa-miR-6818-3p |  |  | LHH | -2.68639 | -2.16324 |  |
| hsa-miR-129-2-3p |  |  |  | -1.038802 | -3.43128 |  |
| hsa-miR-151a-3p |  |  | LMH | -6.39439 | -2.16527 |  |
| hsa-miR-23a-5p |  |  | LHH | -2.02848 | -1.75602 |  |
| hsa-miR-24-2-5p |  |  | LHH | -1.71886 | -1.40758 |  |
| hsa-miR-3614-5p |  |  |  | 1.122898 | -2.02722 |  |
| hsa-miR-3913-5p |  |  | LMH | -3.20616 | -1.55947 |  |
| hsa-miR-425-5p |  |  | LMH | -2.28772 | -1.20565 |  |
| hsa-miR-455-5p |  |  | LHH | -1.76716 | -1.54091 |  |
| hsa-miR-4664-3p |  |  | LMH | -5.53493 | -3.29599 |  |
| hsa-miR-4796-5p |  |  | LHH | -1.87554 | -1.46873 |  |
| hsa-miR-499a-5p |  |  | LHH | -2.37169 | -2.01809 |  |
| hsa-miR-509-3-5p |  |  | LHH | -3.85488 | -3.65709 |  |
| hsa-miR-516b-5p |  |  |  | -1.997767 | -5.33705 |  |
| hsa-miR-517b-3p |  |  |  | -0.962218 | -2.88573 |  |
| hsa-miR-542-3p |  |  |  | -0.054795 | -2.85975 |  |
| hsa-miR-548av-3p |  |  | LMH | -2.7796 | -1.34114 |  |
| hsa-miR-6502-5p |  |  | LHH | -1.29331 | -1.47636 |  |
| hsa-miR-6514-5p |  |  |  | -0.66543 | -1.12007 |  |
| hsa-miR-659-5p |  |  | LMH | -4.46118 | -2.7881 |  |
| hsa-miR-6802-3p |  |  | LHH | -1.41856 | -1.08874 |  |
| hsa-miR-3191-3p |  |  | LMH | -3.11946 | -1.71569 |  |
| hsa-miR-2115-5p |  |  |  | 2.4171329 | -3.326936 |  |
| hsa-miR-6503-3p |  |  | LHH | -2.428916 | -1.980449 |  |
| hsa-miR-365a-5p |  |  | LHH | -2.733208 | -2.750971 |  |
| hsa-miR-3158-3p |  |  | LMH | -5.624209 | -3.858296 |  |
| hsa-miR-192-5p |  |  | LHH | -1.905083 | -1.780492 |  |
| hsa-miR-511-5p |  |  | LMH | -3.50332 | -2.174842 |  |

*LMH: the expressions in LTPPs, RPs and HCs show continuously upregulation from low to middle then to high; LHH: LTPPs, RPs and HCs show low, high, and high expression levels, respectively.

##

## Figure Legends

**Figure S1**. **RNA expression profiles among LTPP, RP and HC groups.** Venn diagram (left) and boxplot (right) show the gene number and abundance for all expressed RNAs (a), mRNAs (b), and lncRNAs (c), and miRNAs (d), respectively. Differences between groups were estimated using Kruskal-Wallis test.

**Figure S2. Expression profiles of the DEmRNAs, DElncRNAs and DEmiRNAs among LTPP, RP and HC groups.** Volcano plots (left) and heatmaps (right) showing the expression patterns of DEmRNAs (a), DElncRNAs (b) and DEmiRNAs (c) among the three groups (log2FC >= 1.5). The top10 significantly up-regulated and down-regulated DEmRNAs, DElncRNAs, and DEmiRNAs are shown in the volcano plots. The colored bars above the heatmaps represent the LTPP (Red), RP (Orange) and HC (Green) samples. The color key indicates the scaled expression levels of the DEmRNAs, DElncRNAs and DEmiRNAs among the three groups.

**Figure S3. Immune cell composition in the PBMCs predicted by the CIBERSORT algorithm from acute infection COVID-19 patients, LTPPs, RPs and HCs using transcriptomic data.** Bar plot showing the percentage of immune cell types in PBMCs. Different colors represent different types of immune cells.

**Figure S4. The interaction network of the screened 38 DEGs and 65 corresponding miRNAs.** Shades of green and red indicate down- or up-regulated genes and corresponding miRNAs in LTPP/RP group, respectively. The mRNAs and miRNAs are shown as circles and V-shape, respectively.

**Figure S5. Dual-luciferase reporter assays for the validation of miRNA-mRNA pairs.** (a) The miRNA binding sites in the 3’UTR of putative targeting mRNAs and the mutated 3’UTR are shown. (b) The luciferase activity was detected in HEK293T cells co-transfected with miRNA mimics or miR-NC (negative control mimic) and 3’UTR-WT (wild type) or MT (mutant) constructs. Empty vector was transfected as a negative control. Firefly luciferase activity was normalized against Renilla luciferase activity and relative luciferase activity of miRNA mimic transfected wells was compared to miR-NC. The data were presented as the means ± SD (n = 3). Unpaired two tailed t-test was used.
